# Geographic disparities in gastrointestinal oncology research: a focus on trial availability in Italy

**DOI:** 10.1093/oncolo/oyaf011

**Published:** 2025-03-27

**Authors:** Maurizio Polignano, Nicola Carella, Ornella Rotolo, Natalino Vena, Vincenza Lorusso, Giuseppe Dalfino, Gianluigi Giannelli

**Affiliations:** National Institute of Gastroenterology - IRCCS “Saverio de Bellis,” 70013 Castellana Grotte, Italy; National Institute of Gastroenterology - IRCCS “Saverio de Bellis,” 70013 Castellana Grotte, Italy; National Institute of Gastroenterology - IRCCS “Saverio de Bellis,” 70013 Castellana Grotte, Italy; National Institute of Gastroenterology - IRCCS “Saverio de Bellis,” 70013 Castellana Grotte, Italy; National Institute of Gastroenterology - IRCCS “Saverio de Bellis,” 70013 Castellana Grotte, Italy; National Institute of Gastroenterology - IRCCS “Saverio de Bellis,” 70013 Castellana Grotte, Italy; National Institute of Gastroenterology - IRCCS “Saverio de Bellis,” 70013 Castellana Grotte, Italy

**Keywords:** oncology, inequity of access, clinical trials, gastrointestinal cancers, North–South divide

## Abstract

**Background:**

Gastrointestinal cancers pose a significant global health burden, bearing as they do high incidence and mortality rates. Clinical trials in oncology offer numerous advantages as helping to develop new treatments and improve existing ones, leading to better patient outcomes, providing patients with access to cutting-edge therapies that might not otherwise be available and enhancing our understanding of cancer biology.

**Methods:**

We retrospectively reviewed active interventional clinical trials in Italy in the field of gastrointestinal neoplasms in the period March 1, 2020 and March 1, 2024, by a search on the “clincaltrials.gov” database. The search yielded 103 studies active in Italy. For each study, the Centers in Italy at which they are active were extracted. Studies resulted active in a total of 630 locations.

**Results:**

The data analysis, by a kernel smoothing for probability density estimation, reveals a pronounced clustering of trials in Northern and Central Italy, while the Southern regions and islands exhibit lower trial availability, highlighting disparities in patient access. The mean number of clinical trials per 100 000 inhabitants was calculated. We found that Northern regions show a much higher concentration compared with the Southern regions and islands (North-east 0.92 CTs/100 000 inhabitants vs Islands 0.53 CTs/100 000 inhabitants).

**Conclusions:**

The uneven distribution does not only limit treatment options for patients in less accessible areas but also raises concerns about the representativeness of trial data. This study underscores the need for targeted strategies to enhance trial accessibility, including decentralized trial models and national databases, to ensure equitable patient participation across Italy.

**Implications for Practice**

The effectiveness of clinical research is based on the ability to enroll patients within a short-time frame and to evaluate the results found based on a sample as representative as possible of the National population. The availability of very up-to-date data on the distribution of clinical trials in Italy on GI tumors can help Promoters to identify areas that guarantee satisfactory enrollment as well as patients in identifying a Center at which to access experimental treatments.

## Introduction

Gastrointestinal cancer includes malignant conditions of the gastrointestinal tract (GI tract) and accessory organs of digestion, the esophagus, stomach, biliary system, pancreas, small intestine, large intestine, rectum, and anus. The symptoms relate to the organ affected and can include obstruction (leading to difficulty in swallowing or defecating), abnormal bleeding, or other associated problems. The diagnosis often requires endoscopy, followed by biopsy of suspicious tissue. The treatment depends on the location of the tumor, as well as the type of cancer cell and whether it has invaded other tissues or spread elsewhere. These factors also determine the prognosis.

Overall, the GI tract and the accessory organs of digestion (pancreas, liver, gall bladder) are responsible for more cancers and more deaths from cancer than any other system in the body.^1,2^ There is a significant geographic variation in the rates of different gastrointestinal cancers.^3^ There were an estimated 4.8 million new cases of GI cancers and 3.4 million related deaths, worldwide, in 2018. Gastrointestinal cancers account for 26% of the global cancer incidence and 35% of all cancer-related deaths.^4^

Developing a new pharmaceutical product is a long and complex process with a high risk both of failure and of missing the goal to successfully develop an effective and safe medication. Analysis across all therapeutic areas indicates that the development of a new pharmaceutical product, from target identification through to marketing authorization, takes about 12 years, and often significantly longer.^5^

Clinical trials have a profound economic impact on the National Healthcare Service (NHS), for example E. Walter et al. demonstrated that, in Austria, €116 22 million spent in Interventional CTs (ICTs) generated a total value added of €144 million, €74 million in direct investment, in 2018. Each year, a medical treatment value of €100 million was financed through 463 ICTs, with an average medical treatment value of €37.068 per recruited patient. This accounts for a significant 0.3% of annual current health-expenditures.^6^ In Italy, in the period 2017-2020, of a total of almost €319 million detected as direct investment, more than €623 million were measured as indirect investment (Averted Cost), with a leverage effect of 2.95. This indicates that for every euro invested by sponsoring companies in clinical trials, the NHS receives an overall benefit of almost €3.^7^ This kind of analysis was also performed in an Italian IRCCS assessing active trials in non oncology (IBDs CT) at an Italian center with comparable results.^8^

Clinical trials in oncology offer numerous advantages. They help to develop new treatments and improve existing ones, leading to better patient outcomes. Trials provide patients with access to cutting-edge therapies that might not otherwise be available. They also enhance our understanding of cancer biology, paving the way for future research. Additionally, clinical trials contribute to establishing new standards of care and can offer hope to patients with limited treatment options. Clinical trials are the obligatory tool not only for the approval of new therapies (EU Reg. 5/2019) but also for early access to experimental drugs (DM September 07, 2017), for off-label use (L 94/98) and in general for access to drugs not supplied by the NHS (L 326/03). Promoting quality trials, which are also fully representative of regional differences in the population, is certainly a challenge for the regulatory authorities and a goal to achieve quality scientific research.^9^

In the last few years, we have seen many eagerly awaited advances in gastrointestinal oncology. This fast-evolving landscape is reflected in new drug authorizations in therapy. More attention is also paid not only to novel treatment approaches but also to data on the molecular characteristics of GI cancers and their classification, translational research, and precision oncology. Although knowledge in the field is growing, there is still a major clinical need for tools to make a better prognosis.

Italy is a country marked by severe structural and economic contrasts across different areas: Italian regions differ greatly in terms of demographic patterns, economic performance, well-being, and institutional quality.^10^ Median age is also clearly different among the regions. For these reasons, it is crucial to ensure the widest possible access to patients in order to allow the trials to collect as representative as possible a sample of the Italian population. The introduction in 1999 (D.lgs June 19, 1999 n.229) of the regionalization of the NHS has somewhat aggravated this situation. The Italian NHS, like that in many other European countries, is a tax-funded system whose provision is mostly decentralized by region, offering patients the freedom to choose healthcare providers. It includes 20 autonomous Regional Health Services, allowing patients to receive healthcare free of charge in either public or licensed private health structures across the country.^11^ In particular, the NHS ensures healthcare services for citizens registered with the local healthcare agencies in their own region of residence. Nevertheless, citizens have the right to receive healthcare services in facilities located in other regions, a possibility that gives rise to interregional healthcare mobility.

In Italy, the decision where to initiate new studies is left to the Sponsor and no governmental activities (or bodies responsible for clinical trials) have ever been instituted to promote the carrying out of trials in as uniform a manner as possible throughout the country. Moreover, the risk of failure of enrollment targets due to excessive local concentrations of competitive trials should not be forgotten. In addition, the unavailability of clinical trials in large areas of the country contributes to health mobility, becoming an additional cost for patients and caregivers. Several studies have highlighted issues related to the accessibility of trials,^12^ but no works have specifically analyzed the situation in Italy.

## Materials and Methods

### Trials data

We retrospectively reviewed active interventional clinical trials in Italy in the field of gastrointestinal neoplasms in the period March 01, 2020 to March 01, 2024. The search was conducted using the “clinicaltrials.gov” database. First, all trials in oncology in the reference period were searched in the database, the only requirement being the inclusion of at least one active center in Italy, by filtering with words such as (neoplasm, cancer, tumor, metastatic, etc.). The search yielded 962 trials (Supplementary Table S1). Specifically, only interventional trials were selected as they offer treatments and therapies that are not available in normal clinical practice due to their nature. From this first selection, trials were retrieved which specifically study gastrointestinal neoplastic diseases, by applying a simple filter to the dataset and reviewing all the extracted trials; this yielded 103 studies active in Italy (Supplementary Table S2). These trials cover the main gastrointestinal oncological diseases. For each study, the Centers in Italy at which they are active were extracted. Studies resulted active in a total of 630 locations, and clearly each Center can have several different studies active at the same time (Supplementary Table S3). For each location (*n* = 67), Center name, City, Region and postal code (postcode, CAP) were identified

### Demographic analysis of the Italian context

To compare the distribution of CTs across the country with the resident population data, we collected population data from the Italian Statistics Agency, ISTAT,^13^ which provides certified data on various Italian socio-economic parameters, and these data are free to access. In particular, we extracted data on resident population, regional land area, and population density from the public dataset and subsequently aggregated them into the 5 regional Italian regions (north-west, north-east, center, south, and islands). From the data thus analyzed, we were able to determine the average ratio between CTs and resident population, the parameter chosen to evaluate the differences in accessibility to the CTs under investigation in this study.

### Analysis of the distribution of clinical trials across the territory

We divided the Italian territory into 5 macro-areas denoted as north-east (Emilia-Romagna, Friuli-Venezia Giulia, Trentino-Alto Adige, and Veneto regions), north-west (Liguria, Lombardy, Piedmont, and Valle d’Aosta regions), Center (Lazio, Marche, Tuscany, Umbria, and Abruzzo regions), South (Abruzzo, Basilicata, Calabria, Campania, Molise, and Apulia regions), and Islands (Sicily and Sardinia regions). This classification was made according to the national standard geographical areas definition. We calculated the level of territorial coverage of the trials using the commercial application (see Statistical analysis). For each center, the population living within 10, 50 and 100 km, respectively, was determined (Supplementary Table S6). The presence of areas of the country with a higher concentration of trials (calculated both in number and as the trial/resident population ratio) was also determined. The presence of clusters and the level of territorial spread were the parameters assessed to determine the accessibility of trials for the population. The levels of overlap measured on the basis of the areas of influence are shown by generating heat maps (measurement of cells 70 miles, cell width 1.70 miles), with the quartic density method.

### Statistical analysis

We used the software Maptitude v. 2024.6040 Caliper Corporation, to make a kernel density estimation, an application of kernel smoothing for probability density estimation, to estimate the probability density function of a random variable based on kernels as weights. Kernel density estimation is a nonparametric model used for estimating probability distributions.

All the mean values are expressed with SD values. The calculations were conducted in Microsoft Excel (Version 16.89.1) for MacOS and R version 4.4.1 (2024-06-15) on Windows 11 OS.

## Results

From 962 interventional trials (Supplementary Table S1) extracted from the database (clinicaltrials.gov), we identified 103 studies in gastroenterological oncology, by registration number on the database (Supplementary Table S2). The clinical trials identified, in the field of therapies for GI cancers, were grouped by type of tumors under study (Table 1); 67 Clinical Centers performing CT were identified for each Clinical Center the number of active CTs was determinated (Supplementary Table S3). Trials were grouped by study phase (1-4) (Supplementary Table S4) and typology of promoter (Supplementary Table S5)

**Table 1.** List of gastrointestinal pathologies treated in the CTs under review with the indication of new cases in 2020. Data on new cases in 2020 (latest available) were collected from the AIOM report.

Pathology	Number	New cases in 2020*
Biliary tract cancer	8	5400
Colorectal cancer	57	48 100
Gastric-esophageal cancer	13	14 700
Hepatocellular carcinoma	10	12 100
Pancreatic cancer	9	14 500
Various GI cancer	6	-
**Total**	103	

The CT’s locations were classified by region and grouped according to their geographical distribution in the 5 Italian economic areas (north-west, north-east, center, south, islands) as shown in Table 2. A total of 630 locations was found at which it is possible to be enrolled in clinical trials for GI oncological pathologies.

**Table 2.** Number of active trials grouped according to their geographical distribution in the 5 Italian areas

Area	Region	N. of CT	
North-West	Liguria	14	
	Lombardy	179	
	Piedmont	29	
	Total	222	35.24%
North-East	Emilia Romagna	63	
	Friuli Venezia Giulia	9	
	Veneto	73	
	Total	145	23.02%
Center	Abruzzo	1	
	Lazio	46	
	Marches	10	
	Tuscany	72	
	Umbria	1	
	Total	130	20.63%
South	Basilicata	4	
	Calabria	3	
	Campania	59	
	Apulia	39	
	Total	105	16.67%
Islands	Sardinia	11	
	Sicily	17	
	Total	28	4.44%
Total		630	

**Figure 1.**
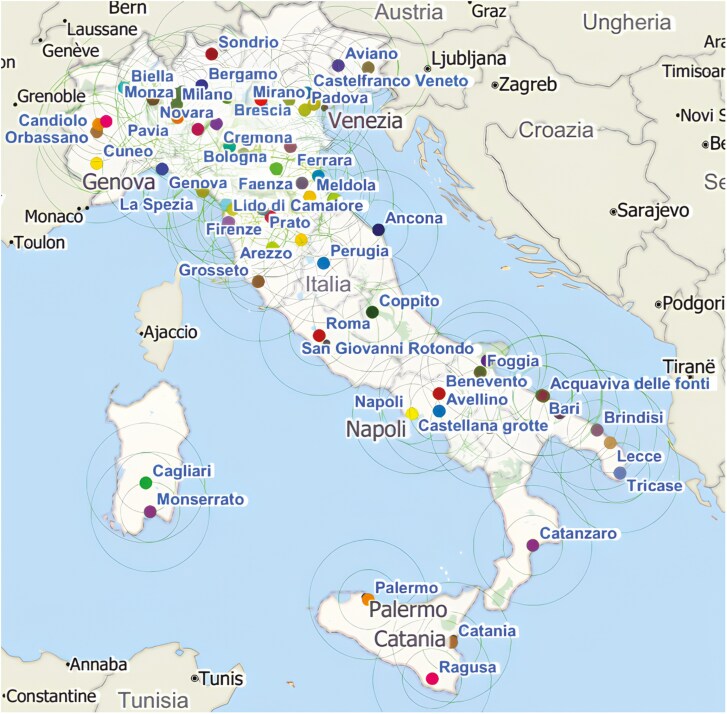
Distribution of clinical centers involved in CT in gastroenterological oncology. Areas of influence at 10, 50, and 100 km from the center are drawn for each Clinical Center.

The Clinical Centers thus identified have been indicated on a map to show the national layout of all mapped Centers, and their areas of influence, as represented by circles with a radius of 10, 50 and 100 km, respectively (Figure 1).

As is evident, there is a strong overlap between the radii in the northeast, northwest, and central areas, while in the south and islands, the circles are much less overlapping.

**Figure 2.**
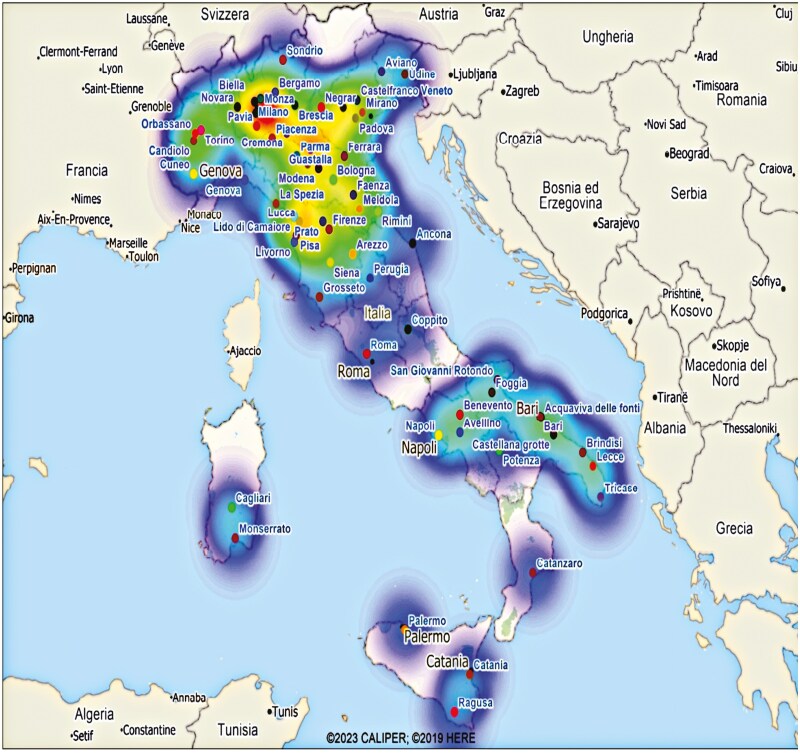
A visualization of the spatial distribution and overlap intensity among clinical center areas. Lower values indicate sparse overlap, while higher values represent greater overlap.

This phenomenon is most visible with the density graphs (red indicates a greater overlap due to the presence of a greater number of active trials, while light blue or no color indicates little or no overlap) (Figure 2). First, clusters were identified, that is, areas in the territory with a high number of clinical trials in the same area.

Having mapped the data thus collected, we calculated the average population within 10, 50 and 100 km, per geographic area, of the trial site (*n* = 67) using software (dataset is in Supplementary Table S4). The results are shown in Table 3.

**Table 3.** Mean population and SD at 10, 50, and 100 km from the Clinical Centers, grouped per geographic area.

	10 km	SD	50 km	SD	100 km	SD
North-east	218 685	218 013	2 138 043	374 693	6 428 345	845 509
North-west	380 967	418 854	2 633 651	2 045 446	6 621 940	3 323 239
Center	157 327	122 553	1 256 403	885 104	3 786 959	1 355 421
South	257 434	447 861	1 520 262	1 346 193	3 127 296	1 896 624
Islands	266 580	179 952	876 886	1 390 136	1 945 728	2 049 375

To compare the number of clinical trials detected during the observation period with the population and the regional area, we collected demographic data (ISTAT, year 2022) by Region and compared it with the number of CTs. The data collected are tabulated in Table 4.

**Table 4.** Mean population and SD at 10, 50, and 100 km from Clinical Centers, grouped per geographic area.

Region	Geographic area	Population	Area	Density of population	Number of CT
		N. of inhabitants	km²	Inhabitants/km²	CT
Veneto	North-East	4 851 972	18 351 49	264	73
Emilia-Romagna	North-East	4 455 188	22 501 82	198	63
Trentino-Alto Adige	North-East	1 082 116	13 605 97	80	0
Friuli Vnenezia Giulia	North-East	1 195 792	7 936 83	151	9
Lombarardy	North-West	10 020 528	23 862 87	420	179
Piedmont	North-West	4 252 581	25 391 67	167	29
Liguria	North-West	1 508 847	5 417 71	279	14
Valle D’Aosta	North-West	123 018	3 258 61	38	0
Lazio	Center	5 720 272	17 236 49	332	46
Tuscany	Center	3 664 798	22 985 01	159	72
Marches	Center	1 484 427	9 344 54	159	10
Umbria	Center	854 378	8 463 97	101	1
Campania	South	5 590 076	13 667 85	409	59
Apulia	South	3 890 250	19 541 03	199	39
Calabria	South	1 838 150	15 212 65	121	3
Abruzzo	South	1 269 963	10 828 89	117	1
Basilicata	South	533 636	10 071 59	53	4
Molise	South	289 413	4 459 80	65	0
Sicily	Islands	4 794 512	25 824 33	186	17
Sardinia	Islands	1 569 832	24 106 30	65	11

To make this phenomenon more numerically evident, the number of active trials per 100 000 inhabitants was also determined and reported, as mean value, for north-west, north-east, Center, South and Islands, corresponding to 0.85 (SD 0.67), 0.92 (SD 0.70), 0.89 (SD 0.78), 0.51 (SD 0.48), and 0.53 (SD 0.24), respectively (see Tables 4 and 5).

**Table 5.** Coverage of the CT population in gastroenterological oncology. Accessibility is expressed as mean number of trials per 100 000 inhabitants for each of the 5 Italian geographical areas.

	Mean value	SD
*North-west*	0.85	0.67
*North-east*	0.92	0.70
*Center*	0.89	0.78
*South*	0.51	0.48
*Islands*	0.53	0.24

## Discussion

Major cancers of the GI tract affect 78 000 men and women in Italy every year. Specifically, the means are 43 700 cases of colorectal cancer; 14 500 stomach cancer; 14 300 pancreatic cancer, and 5400 cholangiocarcinoma. These are all diseases that are still too often diagnosed late. This forces medical specialists to have to try to treat neoplasms at an advanced stage because few effective and quality-of-life-friendly therapies are available for patients at this stage. For colorectal cancer alone, more than 8700 cases per year are detected when they have already developed metastases. In the case of stomach cancer, only 7% of malignancies are diagnosed in the early stages.^14^

The strong clustering of the distribution of trials is evident, with a much greater presence of trials in the areas of Central-Northern Italy (see Figure 2). In particular, a strong clustering of CTs (*n* = 630) is evident in the north-east and north-west areas of Italy, equal, overall, to 58.26% of all the trials detected (*n* = 367 active trials), followed by Central Italy (20.63%, *n* = 130), mainly concentrated in the Rome area. The South and the Islands together only slightly exceed 20% of the detected CTs (*n* = 133). In particular, the islands have only 28 active CTs (4.44% of the total).

To relate the number of CTs to a demographic parameter, we calculated the number of CTs/100 000 inhabitants, from data extracted from the ISTAT database. The results confirm the substantially higher presence of CTs in the regions of central and northern Italy; the CTs/population ratio in the north-east (0.92 CTs/100 000 inhabitants) is 73% higher than the lowest value determined (Islands: 0.53 CTs/100 000 inhabitants).

We have, for the first time in a research work, calculated the average population per geographical area resident within a radius of 10, 50, and 100 km from each Clinical Center (Supplementary Table S6). As is evident, there is a profound difference between the various geographical areas. Within 100 km of each Clinical Center, an average of 6 621 940 inhabitants are to be found in the north-west (highest figure), while only 1 945 728 inhabitants live on the islands (lowest figure surveyed). Obviously, this translates into a much lower probability for a patient living in the major Italian islands (Sicily and Sardinia) to find a clinical trial in which to participate, compared with the much higher probability for a patient living in the north-east of Italy. Although the European Clinical Trials Regulation (Reg. 536/2014) allows for the reimbursement of out-of-pocket expenses incurred, this does not consider incidental expenses for accompanying persons, lost working days, and the time required for organizing frequent trips to the trial center. No data are available on the patients enrolled in each trial and the relative distance from the Clinical Center, but it is quite clear that the chances of treatment for a patient living in the north and in insular Italy cannot be considered in any way comparable.

In Italy, unfortunately, “health literacy” is still very poor and obtaining, processing and understanding health information or accessing health services in an informed manner is still difficult for many people. It is also difficult to get information on clinical trials, to know where to look for them and to think about being selected as a participant.^15^ All this is still too strongly limited to selected segments of the population, usually the better educated and of higher socio-economic status.^16^

As is evident, the unavailability of a Clinical Center at an acceptable distance for the patient to be studied is a barrier to enrollment. This situation essentially has 2 effects: first, the possibility of access to treatment, that is often life-saving, only if the patient is resident in certain specific geographical areas, caused by the lack of true population coverage.^17^

Second, research has demonstrated that many groups that are underrepresented and excluded in clinical research can have distinct disease presentations or health circumstances that affect how they will respond to an investigational drug or therapy.^18-20^ Such differences contribute to variable therapeutic responses and necessitate targeted efficacy and safety evaluations.

Thus, lack of equal representation in clinical trials has consequences on health outcomes and may contribute to persistent health disparities.^21^ These barriers damage the credibility of clinical trials and end up generating evidence that is unverified in the real world.

Decentralized Clinical Trials (DCTs) may well make trial participation broadly accessible, less burdensome, and more attractive. It is becoming more and more evident that inclusivity propelled by DCTs will also have to overcome broader industry trends that consistently make trial participation more difficult for all participants.^22^

The reasons for this phenomenon are certainly many. The regionalization of the NHS and the presence within the regional decision-makers of a greater or lesser “sensitivity” to the topic of promoting clinical trials is more than evident. Also, an important role is played by KOLs often active in Centers of great national importance which tend to attract most of clinical trials and by Promoters and CROs which tend to “risk” less by focusing on better known Centers.

Substantial improvements will require considerable changes in culture in educational and professional practice, research, and regulatory communities, and will require major increases in public, corporate, and philanthropic funding.

## Conclusion

An analysis of the data in Italy shows that it appears to be, at least in the field of clinical research, a “differentiated speed” Country. The equity of accessibility to Clinical trials is an avenue of excellence for the evaluation of new drug therapies in the treatment of gastrointestinal cancer diseases. The participation of patients’ representative of different geographical areas is of paramount importance for obtaining reliable data on the efficacy of therapies. The excessive clustering of clinical trials in Italy and the marked national differences in terms of accessibility are detrimental to patients, who are often forced to travel long distances to reach the Clinical Centers and, overall, to the attractiveness of our country in the field of interventional clinical trials, mainly due to an objective difficulty in reaching the enrollment targets set by the promoters. This phenomenon is partly due to the availability, in the various territories, of Clinical Centers suitable for carrying out CTs according to the standards required by the Good Clinical Practices, probably linked to the economic capacity of the territories, but also to the choices of Promoters and CROs, who are often “unwilling” to invest in the activation of Clinical Centers that are unknown to them. The lack of a National network that directs patients with conditions compatible with the clinical trial inclusion criteria to the nearest Clinical Center, as well as the lack of a National database of active clinical trials that can be used by patients associations, do not help to foster a uniform spread throughout the country and indeed, only exacerbate the inequality revealed by the data analysis in access to CT.

The uneven distribution of clinical trials across the territory also represents a missed development opportunity for certain areas of the country that cannot benefit from the advantages, including economic ones, of conducting CT.

Certainly, much can be done to reverse the trend toward clustering CTs, mainly by raising awareness among Clinical Centers and Sponsors about the importance of conducting interventional drug trials, as well as creating a regional ecosystem favorable to clinical research, and national laws that promote and simplify trial activation processes.

## Supplementary Material

oyaf011_suppl_Supplementary_Tables_S1

oyaf011_suppl_Supplementary_Tables_S2-S22

oyaf011_suppl_Supplementary_Tables_S3

oyaf011_suppl_Supplementary_Tables_S6

oyaf011_suppl_Supplementary_Tables_S5

oyaf011_suppl_Supplementary_Tables_4

## Data Availability

The authors confirm that the data supporting the findings of this study are available within the article and its supplementary materials.
