# Supplementary material for: Geographic disparities in gastrointestinal oncology research: a focus on trial availability in Italy
Source: Oncologist. 2025 Mar 27;30(3):oyaf011. doi: 10.1093/oncolo/oyaf011 (PMC11950913; doi:10.1093/oncolo/oyaf011)
Supplement: oyaf011_suppl_Supplementary_Tables_S1 [file oyaf011_suppl_supplementary_tables_s1.pdf]

STUDY REGISTRATION CODE	STUDY TITLE
NCT03462212	""Carboplatin-Paclitaxel-Bevacizumab vs Carbo-Pacli-Beva-Rucaparib vs Carbo-Pacli-Ruca
NCT03462212,	"Carboplatin-Paclitaxel-Bevacizumab vs Carbo-Pacli-Beva-Rucaparib vs Carbo-Pacli-Ruca, Selected According to HRD Status, in Patients With Advanced Ovarian, Primary Peritoneal and Fallopian Tube Cancer, Preceded by a Phase I Dose Escalation Study on Ruca-Beva Combination"
NCT03708900,	"Pharmacokinetic (PK), Pharmacodynamic (PD) and Tolerability of Osilodrostat in Pediatric Patients with Cushing's Disease"
NCT03804372,	"The Incidence of Hepatitis B in Diffuse Large B-Cell Lymphoma/Chronic Lymphoid Leukemia HBsAg-positive Treated With Rituximab, Chemotherapy and Tenofovir Alafenamide"
NCT03838744,	"Randomized Trial in Advanced, Metastatic or Unresectable Soft Tissue Sarcoma After Failure of Standard Treatments."
NCT04094610,	"A Study of Repotrectinib in Pediatric and Young Adult Subjects Harboring ALK, ROS1, OR NTRK1-3 Alterations"
NCT04152863,	"Efficacy, Safety, and Tolerability of Gebasaxturev (V937) Administered Intravenously or Intratumorally With Pembrolizumab (MK-3475) Versus Pembrolizumab Alone in Participants With Advanced/Metastatic Melanoma (V937-011)"
NCT04154189,	"A Study to Compare the Efficacy and Safety of Ifosfamide and Etoposide With or Without Lenvatinib in Children, Adolescents and Young Adults With Relapsed and Refractory Osteosarcoma"
NCT04168502,	"Gemtuzumab Chemotherapy MRD Levels; Glasdegib Post-transplant, Adult Untreated, de Novo, Fav Interm Risk AML"
NCT04181827,	"A Study Comparing JNJ-68284528, a CAR-T Therapy Directed Against B-cell Maturation Antigen (BCMA), Versus Pomalidomide, Bortezomib and Dexamethasone (PVd) or Daratumumab, Pomalidomide and Dexamethasone (DPd) in Participants With Relapsed and Lenalidomide-Refractory Multiple Myeloma"
NCT04187404,	"A Novel Therapeutic Vaccine (EO2401) in Metastatic Adrenocortical Carcinoma, or Malignant Pheochromocytoma/Paraganglioma"
NCT04191616,	"Study of Early Relapsed, Lenalidomide-refractory Subjects Eligible for Carfilzomib Triplet"
NCT04224493,	"A Study to Assess the Efficacy, Safety, Pharmacodynamics, and Pharmacokinetics of Tazemetostat in Combination With Lenalidomide Plus Rituximab Versus Placebo in Combination With Lenalidomide Plus Rituximab in Adult Patients at Least 18 Years of Age With Relapsed/Refractory Follicular Lymphoma."
NCT04238819,	"A Study of Abemaciclib (LY2835219) in Combination With Other Anti-Cancer Treatments in Children and Young Adult Participants With Solid Tumors, Including Neuroblastoma"
NCT04239014,	"A Study to Evaluate the Effectiveness and Tolerability of a Second Maintenance Treatment in Participants With Ovarian Cancer, Who Have Previously Received Polyadenosine 5'Diphosphoribose [Poly (ADP Ribose)] Polymerase Inhibitor (PARPi) Treatment."
NCT04240002,	"A Study of Gilteritinib (ASP2215) Combined With Chemotherapy in Children, Adolescents and Young Adults With FMS-like Tyrosine Kinase 3 (FLT3)/Internal Tandem Duplication (ITD) Positive Relapsed or Refractory Acute Myeloid Leukemia (AML)"
NCT04246047,	"Evaluation of Efficacy and Safety of Belantamab Mafodotin, Bortezomib and Dexamethasone Versus Daratumumab, Bortezomib and Dexamethasone in Participants With Relapsed/Refractory Multiple Myeloma"
NCT04251065,	"Daratumumab Plus Gemcitabine, Dexamethasone, Cisplatin in pt R/R CD38+ PTCL-NOS, AITL and TFH"
NCT04266301,	"Study of Efficacy and Safety of MBG453 in Combination With Azacitidine in Subjects With Intermediate, High or Very High Risk Myelodysplastic Syndrome (MDS) as Per IPSS-R, or Chronic Myelomonocytic Leukemia-2 (CMML-2)"
NCT04270409,	Isatuximab in Combination With Lenalidomide and Dexamethasone in High-risk Smoldering Multiple Myeloma
NCT04285567,	"A Study to Compare the Efficacy and Safety of a Combined Regimen of Venetoclax and Obinutuzumab Versus Fludarabine, Cyclophosphamide, and Rituximab (FCR)/ Bendamustine And Rituximab (BR) in FIT Patients With Previously Untreated Chronic Lymphocytic Leukemia (CLL) Without DEL (17P) or TP53 Mutation"
NCT04296890,	"A Study of Mirvetuximab Soravtansine in Platinum-Resistant, Advanced High-Grade Epithelial Ovarian, Primary Peritoneal, or Fallopian Tube Cancers With High Folate Receptor-Alpha Expression"
NCT04332822,	"A Randomized, Multicenter, Phase III Trial Comparing Treatment With R-mini-CHOP With R-mini-CHP + Polatuzumab Vedotin in Patients With Diffuse Large Cell B Cell Lymphoma"
NCT04375956,	"Study on Pembrolizumab in Recurrent, Platinum Resistant, CPS >1 Positive Ovarian, Fallopian Tube and Primary Peritoneal Cancer Patients"
NCT04404140,	"A Study Evaluating The Safety, Efficacy and Pharmacokinetics Of Ipatasertib In Combination With Atezolizumab And Docetaxel In Metastatic Castration-Resistant Prostate Cancer (mCRPC)."
NCT04408118,	"First Line Atezolizumab, Paclitaxel, and Bevacizumab (Avastin¬Æ) in mTNBC"
NCT04416984,	"Safety and Efficacy of ALLO-501A Anti-CD19 Allogeneic CAR T Cells in Adults With Relapsed/Refractory Large B Cell Lymphoma, Chronic Lymphocytic Leukemia and Small Lymphocytic Lymphoma (ALPHA2)"
NCT04449874,	"A Study to Evaluate the Safety, Pharmacokinetics, and Activity of GDC-6036 Alone or in Combination in Participants With Advanced or Metastatic Solid Tumors With a KRAS G12C Mutation"
NCT04459715,	"A Study of Xevinapant (Debio 1143) in Combination With Platinum-Based Chemotherapy and Standard Fractionation Intensity-Modulated Radiotherapy in Participants With Locally Advanced Squamous Cell Carcinoma of the Head and Neck, Suitable for Definitive Chemoradiotherapy (TrilynX)"
NCT04464798,	"A Dose Finding and Safety Study of CC-220, Alone and in Combination With an Anti-CD20 Monoclonal Antibody (mAb) in Subjects With Relapsed or Refractory Lymphomas"
NCT04485104,	"Assessment of Adjunctive Cannabidiol Oral Solution (GWP42003-P) in Children With Tuberous Sclerosis Complex (TSC), Dravet Syndrome (DS), or Lennox-Gastaut Syndrome (LGS) Who Experience Inadequately-controlled Seizures"
NCT04485260,	"An Open-Label, Multicenter, Phase 1b/2 Study of the Safety and Efficacy of KRT-232 Combined With Ruxolitinib in Patients With Primary Myelofibrosis (PMF), Post-Polycythemia Vera MF (Post-PV-MF), Or Post-Essential Thrombocythemia MF (Post ET-MF) Who Have a Suboptimal Response to Ruxolitinib"
NCT04494425,	"Study of Trastuzumab Deruxtecan (T-DXd) vs Investigator's Choice Chemotherapy in HER2-low, Hormone Receptor Positive, Metastatic Breast Cancer"
NCT04501614,	"A Study of Ponatinib With Chemotherapy in Children, Teenagers, and Adults With Philadelphia Chromosome-Positive Acute Lymphoblastic Leukemia"
NCT04502030,	"Double-blind, Randomized, Placebo-controlled, Prospective Phase III Study Evaluating Efficacy and Safety of Panzyga in Primary Infection Prophylaxis in Patients With Chronic Lymphocytic Leukemia (""PRO-SID"" Study)"
NCT04513925,	"A Study of Atezolizumab and Tiragolumab Compared With Durvalumab in Participants With Locally Advanced, Unresectable Stage III Non-Small Cell Lung Cancer (NSCLC)"
NCT04526106,	"REFOCUS: a First-in-Human Study of Highly Selective FGFR2 Inhibitor, RLY-4008, in Patients with ICC and Other Advanced Solid Tumors"
NCT04526899,	"Trial With BNT111 and Cemiplimab in Combination or as Single Agents in Patients With Anti-PD-1-refractory/Relapsed, Unresectable Stage III or IV Melanoma"
NCT04534205,	"A Clinical Trial Investigating the Safety, Tolerability, and Therapeutic Effects of BNT113 in Combination With Pembrolizumab Versus Pembrolizumab Alone for Patients With a Form of Head and Neck Cancer Positive for Human Papilloma Virus 16 and Expressing the Protein PD-L1"
NCT04538664,	"A Study of Combination Amivantamab and Carboplatin-Pemetrexed Therapy, Compared With Carboplatin-Pemetrexed, in Participants With Advanced or Metastatic Non-Small Cell Lung Cancer Characterized by Epidermal Growth Factor Receptor (EGFR) Exon 20 Insertions"
NCT04546009,	"A Study Evaluating the Efficacy and Safety of Giredestrant Combined With Palbociclib Compared With Letrozole Combined With Palbociclib in Participants With Estrogen Receptor-Positive, HER2-Negative Locally Advanced or Metastatic Breast Cancer (persevERA Breast Cancer)"
NCT04580121,	"A Dose Escalation and Expansion Study Evaluating the Safety, Tolerability, Pharmacokinetics, and Pharmacodynamics of RO7283420."
NCT04597125,	"Investigation of Radium-223 Dichloride (Xofigo), a Treatment That Gives Off Radiation That Helps Kill Cancer Cells, Compared to a Treatment That Inactivates Hormones (New Antihormonal Therapy, NAH) in Patients With Prostate Cancer That Has Spread to the Bone Getting Worse on or After Earlier NAH"
NCT04603183,	"ABemaciclib, ET ¬± paclItaxel in aGgressive HR+/HER2- MBC trIaL"
NCT04604067,	"Assessing a ctDNA and PET-oriented Therapy in Patients With DLBCL A Multicenter, Open-label, Phase II Trial."
NCT04604132,	"Derazantinib Alone or in Combination With Paclitaxel, Ramucirumab or Atezolizumab in Gastric Adenocarcinoma"
NCT04607668,	"Trilaciclib, a CDK 4/6 Inhibitor, in Patients Receiving FOLFOXIRI/Bevacizumab for Metastatic Colorectal Cancer (mCRC):"
NCT04620330,	"A Study of Avutometinib (VS-6766) + Defactinib in Recurrent KRAS G12V, Other KRAS and BRAF Non-Small Cell Lung Cancer"
NCT04624204,	"Placebo-controlled, Study of Concurrent Chemoradiation Therapy With Pembrolizumab Followed by Pembrolizumab and Olaparib in Newly Diagnosed Treatment-Na√Øve Limited-Stage Small Cell Lung Cancer (LS-SCLC) (MK 7339-013/KEYLYNK-013)"
NCT04629508,	"To Assess the Safety, Tolerability and Efficacy of Itacitinib Immediate Release Tablets in Participants With Primary or Secondary Myelofibrosis Who Have Received Prior Ruxolitinib and/or Fedratinib Monotherapy (LIMBER-213)"
NCT04640623,	"A Study of TAR-200 in Combination With Cetrelimab, TAR-200 Alone, or Cetrelimab Alone in Participants With Non-Muscle Invasive Bladder Cancer (NMIBC) Unresponsive to Intravesical Bacillus Calmette-Gu√©rin Who Are Ineligible for or Elected Not to Undergo Radical Cystectomy"
NCT04657991,	"A Clinical Trial of Three Study Medicines (Encorafenib, Binimetinib, and Pembrolizumab) in Patients With Advanced or Metastatic Melanoma"
NCT04669119,	"Effects of Bromelain and Boswellia Serrata Casperome With Centella Asiatica and Vitamins on Edema, Paresthesia and Postoperative Pain After Quadrantectomy With or Without Sentinel Lymph Node Biopsy."
NCT04669171,	"A Novel Vaccine (EO2463) as Monotherapy and in Combination, for Treatment of Patients With Indolent Non-Hodgkin Lymphoma"
NCT04677504,	"A Study of Atezolizumab With or Without Bevacizumab in Combination With Cisplatin Plus Gemcitabine in Patients With Untreated, Advanced Biliary Tract Cancer"
NCT04679064,	"Trial on NIraparib-TSR-042 (Dostarlimab) vs Physician's Choice CHEmotherapy in Recurrent, Ovarian, Fallopian Tube or Primary Peritoneal Cancer Patients Not Candidate for Platinum Retreatment"
NCT04686305,	"Phase Ib Study of the Safety of T-DXd and Immunotherapy Agents With and Without Chemotherapy in Advanced or Metastatic HER2+, Non-squamous NSCLC"

NCT04689347,	"5FU/LV, Irinotecan, Temozolomide and Bevacizumab for MGMT Silenced, Microsatellite Stable Metastatic Colorectal Cancer."
NCT04702425,	"VOB560-MIK665 Combination First in Human Trial in Patients With Hematological Malignancies (Relapsed/Refractory Non-Hodgkin Lymphoma, Relapsed/Refractory Acute Myeloid Leukemia, or Relapsed/Refractory Multiple Myeloma)"
NCT04702880,	"A Study of BMS-986012 in Combination With Carboplatin, Etoposide, and Nivolumab as First-line Therapy in Extensive-stage Small Cell Lung Cancer"
NCT04703192,	"Valemetostat Tosylate (DS-3201b), an Enhancer of Zeste Homolog (EZH) 1/2 Dual Inhibitor, for Relapsed/Refractory Peripheral T-Cell Lymphoma (VALENTINE-PTCL01)"
NCT04710628,	"Combination of Pembrolizumab and Lenvatinib, in Pre-treated Thymic CArcinoma paTIents"
NCT04718844,	"A Study Investigate the Safety, Tolerability, Pharmacokinetic, and Pharmacodynamic Response of SLN124 in Adults With Alpha/Beta-thalassaemia and Very Low- and Low-risk Myelodysplastic Syndrome"
NCT04722575,	"Combination or Sequence of Vemurafenib, Cobimetinib, and Atezolizumab in High-risk, Resectable Melanoma"
NCT04725188,	"Pembrolizumab/Vibostolimab Coformulation (MK-7684A) or Pembrolizumab/Vibostolimab Coformulation Plus Docetaxel Versus Docetaxel for Metastatic Non Small Cell Lung Cancer (NSCLC) With Progressive Disease After Platinum Doublet Chemotherapy and Immunotherapy (MK-7684A-002, KEYVIBE-002)"
NCT04729387,	"Alpelisib Plus Olaparib in Platinum-resistant/Refractory, High-grade Serous Ovarian Cancer, With no Germline BRCA Mutation Detected"
NCT04730349,	"A Study of Bempegaldesleukin (BEMPEG: NKTR-214) in Combination With Nivolumab in Children, Adolescents and Young Adults With Recurrent or Treatment-resistant Cancer"
NCT04736706,	"A Study of Pembrolizumab (MK-3475) in Combination With Belzutifan (MK-6482) and Lenvatinib (MK-7902), or Pembrolizumab/Quavonlimab (MK-1308A) in Combination With Lenvatinib, Versus Pembrolizumab and Lenvatinib, for Treatment of Advanced Clear Cell Renal Cell Carcinoma (MK-6482-012)"
NCT04758975,	"Venetoclax, Rituximab and Ibrutinib in TN Patients With CLL Undetectable Minimal Residual Disease (uMRD) in Treatment-na√Øve Patients With Chronic Lymphocytic Leukemia (CLL)"
NCT04763928,	"Trial in AML Secondary to MPNs Patients, Unfit for Intensive Chemotherapy, Investigating a Treatment Combination Including Decitabine and Venetoclax"
NCT04768972,	"FUSION: A Study to Evaluate the Efficacy, Safety, Pharmacokinetics and Pharmacodynamics of ION363 in Amyotrophic Lateral Sclerosis Participants With Fused in Sarcoma Mutations (FUS-ALS)"
NCT04774718,	"A Study Evaluating the Safety, Pharmacokinetics, and Efficacy of Alectinib in Pediatric Participants With ALK Fusion-Positive Solid or CNS Tumors"
NCT04784715,	"Trastuzumab Deruxtecan (T-DXd) With or Without Pertuzumab Versus Taxane, Trastuzumab and Pertuzumab in HER2-positive Metastatic Breast Cancer (DESTINY-Breast09)"
NCT04790903,	"A Study Evaluating the Safety, Efficacy and Pharmacokinetics of Venetoclax in Combination With Polatuzumab Vedotin Plus Rituximab (R) and Cyclophosphamide, Doxorubicin, Prednisone (CHP) in Participants With Untreated BCL-2 Immunohistochemistry (IHC)-Positive Diffuse Large B-Cell Lymphoma (DLBCL)"
NCT04809467,	"A Study Evaluating Safety, PK, and Efficacy of Tafasitamab and Parsaclisib in Participants With Relapsed/Refractory Non Hodgkin Lymphoma (R/R NHL) or Chronic Lymphocytic Leukemia (CLL)"
NCT04825990,	"Pembrolizumab and Olaparib in Recurrent/Metastatic, Platinum Resistant Nasopharyngeal Cancer"
NCT04833894,	"Evaluating the Pharmacokinetics, Pharmacodynamics, and Safety of Efgartigimod Administered Intravenously in Children With Generalized Myasthenia Gravis"
NCT04842604,	"Continuation Study of B1371019(NCT03416179) and B1371012(NCT02367456) Evaluating Azacitidine With Or Without Glasdegib In Patients With Previously Untreated AML, MDS or CMML"
NCT04868877,	"Phase 1/2 Study Evaluating MCLA-129, a Human Anti-EGFR, Anti-c-MET Bispecific Antibody, in Advanced NSCLC and Other Solid Tumors, Alone and in Combination"
NCT04875026,	"Frequency and Intensity of Local Reactions in Patients Treated With 4% 5-FU vs 4% 5-FU Associated With an Emollient Cream: a Randomised, Controlled Clinical Trial"
NCT04875975,	"A Study to Test the Efficacy, Safety, and Pharmacokinetics of Rozanolixizumab in Adult Study Participants With Leucine-Rich Glioma Inactivated 1 Autoimmune Encephalitis"
NCT04876313,	"An Open Label, Single-arm, Phase 2 Study of Neoadjuvant Nivolumab and Nab-paclitaxel Before Radical Cystectomy for Patients With Muscle-invasive Bladder Cancer (NURE-Combo)"
NCT04893785,	"A Trial Evaluating the Activity and Safety of Combination Between Cabozantinib and Temozolomide in Lung and GEP-NENS Progressive After Everolimus, Sunitinib or PRRT (CABOTEM)"
NCT04910568,	"A Study Evaluating the Safety, Pharmacokinetics, and Activity of Cevostamab in Participants With Relapsed or Refractory Multiple Myeloma"
NCT04915248,	"Study to Evaluate Combined Treatment of Daratumumab, Bortezomib and Dexamethasone in PBL Patients."
NCT04924075,	"Belzutifan/MK-6482 for the Treatment of Advanced Pheochromocytoma/Paraganglioma (PPGL), Pancreatic Neuroendocrine Tumor (pNET), Von Hippel-Lindau (VHL) Disease-Associated Tumors, Advanced Gastrointestinal Stromal Tumor (wt GIST), or Solid Tumors With HIF-2Œ± Related Genetic Alterations (MK-6482-015)"
NCT04924608,	"Efficacy and Safety of Selumetinib in Adults With NF1 Who Have Symptomatic, Inoperable Plexiform Neurofibromas"
NCT04960709,	"Treatment Combination of Durvalumab, Tremelimumab and Enfortumab Vedotin or Durvalumab and Enfortumab Vedotin in Patients With Muscle Invasive Bladder Cancer Ineligible to Cisplatin or Who Refuse Cisplatin"
NCT04961996,	"A Study Evaluating the Efficacy and Safety of Adjuvant Giredestrant Compared With Physician's Choice of Adjuvant Endocrine Monotherapy in Participants With Estrogen Receptor-Positive, HER2-Negative Early Breast Cancer (lidERA Breast Cancer)"
NCT04963270,	"A Study To Evaluate Efficacy, Safety, Pharmacokinetics, And Pharmacodynamics Of Satralizumab In Patients With Generalized Myasthenia Gravis"
NCT04975308,	"A Study of Imlunestrant, Investigator's Choice of Endocrine Therapy, and Imlunestrant Plus Abemaciclib in Participants With ER+, HER2- Advanced Breast Cancer"
NCT04975997,	"Open-label Study Comparing Iberdomide, Daratumumab and Dexamethasone (IberDd) Versus Daratumumab, Bortezomib, and Dexamethasone (DVd) in Participants With Relapsed or Refractory Multiple Myeloma (RRMM)"
NCT04999202,	"A Study to Learn How Safe the Study Drug BAY 2416964 (AhR Inhibitor) in Combination With the Treatment Pembrolizumab is, How This Combination Affects the Body, the Maximum Amount That Can be Given, How it Moves Into, Through and Out of the Body and Its Action Against Advanced Solid Cancers in Adults"
NCT05005442,	"A Study of Pembrolizumab/Vibostolimab (MK-7684A) in Relapsed/Refractory Hematological Malignancies (MK-7684A-004, KEYVIBE-004)"
NCT05011058,	"An Open-Label, Phase 2 Trial of Nanatinostat in Combination With Valganciclovir in Patients With Epstein-Barr Virus-Positive (EBV+) Relapsed/Refractory Lymphomas"
NCT05028348,	"A Study of Combination of Selinexor, Pomalidomide, and Dexamethasone (SPd) Versus Elotuzumab, Pomalidomide, and Dexamethasone (EloPd) in Subject With Previously Treated Multiple Myeloma"
NCT05038735,	"Study to Assess the Efficacy and Safety of Alpelisib Plus Fulvestrant in Participants With HR-postitive (HR+), HER2-negative, Advanced Breast Cancer After Treatment With a CDK4/6 Inhibitor and an Aromatase Inhibitor."
NCT05041257,	"Mirvetuximab Soravtansine Monotherapy in Platinum-Sensitive Epithelial, Peritoneal, and Fallopian Tube Cancers (PICCOLO)"
NCT05043090,	"Savolitinib Plus Durvalumab Versus Sunitinib and Durvalumab Monotherapy in MET-Driven, Unresectable and Locally Advanced or Metastatic PRCC"
NCT05048797,	"A Study to Investigate the Efficacy and Safety of Trastuzumab Deruxtecan as the First Treatment Option for Unresectable, Locally Advanced/Metastatic Non-Small Cell Lung Cancer With HER2 Mutations"
NCT05059327,	"Basimglurant (NOE-101) in Children, Adolescents, and Young Adults with TSC"
NCT05083169,	"A Study of Teclistamab in Combination With Daratumumab Subcutaneously (SC) (Tec-Dara) Versus Daratumumab SC, Pomalidomide, and Dexamethasone (DPd) or Daratumumab SC, Bortezomib, and Dexamethasone (DVd) in Participants With Relapsed or Refractory Multiple Myeloma"
NCT05091424,	"A Study Evaluating the Safety, Efficacy, and Pharmacokinetics of Mosunetuzumab and a Combined Regimen of Mosunetuzumab and Venetoclax in Participants With Relapsed or Refractory Chronic Lymphocytic Leukemia"
NCT05091567,	"A Phase III, Open-Label Study of Maintenance Lurbinectedin in Combination With Atezolizumab Compared With Atezolizumab in Participants With Extensive-Stage Small-Cell Lung Cancer"
NCT05104866,	"A Phase-3, Open-Label, Randomized Study of Dato-DXd Versus Investigator's Choice of Chemotherapy (ICC) in Participants With Inoperable or Metastatic HR-Positive, HER2-Negative Breast Cancer Who Have Been Treated With One or Two Prior Lines of Systemic Chemotherapy (TROPION-Breast01)"
NCT05113251,	"Trastuzumab Deruxtecan (T-DXd) Alone or in Sequence With THP, Versus Standard Treatment (ddAC-THP), in HER2-positive Early Breast Cancer"
NCT05123482,	"A Phase I/IIa Study of AZD8205 Given Alone or in Combination With Anticancer Drugs, in Participants With Advanced or Metastatic Solid Malignancies"
NCT05128630,	"Chemo-immunotherapy, Hypo-fractionated RT and Maintenance Immunotherapy for Stage III NSCLC."
NCT05136326,	"Preoperative Chemoradiotherapy With CApecitabine and Temozolomide in MGMT Silenced, MSS, Locally Advanced RecTal Cancer"
NCT05153330,	"Study of BMF-219, a Covalent Menin Inhibitor, in Adult Patients With AML, ALL (With KMT2A/MLL1r, NPM1 Mutations), DLBCL, MM, and CLL/SLL"
NCT05169515,	"A Study Evaluating the Safety, Pharmacokinetics, and Efficacy of Mosunetuzumab or Glofitamab in Combination With CC-220 and CC-99282 in Participants With B-Cell Non-Hodgkin Lymphoma"
NCT05170204,	"A Study Evaluating the Efficacy and Safety of Multiple Therapies in Cohorts of Participants With Locally Advanced, Unresectable, Stage III Non-Small Cell Lung Cancer (NSCLC)"
NCT05203913,	"Cisplatin, Nab-paclitaxel, Nivolumab With Radiotherapy After Resection of Non-Metastatic Muscle Invasive Bladder Cancer"
NCT05205330,	"CR6086/AGEN2034 Combination in Stage IV pMMR - MSS CRC, and Other Metastatic GI Cancers"
NCT05216432,	"First-in-Human Study of Mutant-selective PI3KŒ± Inhibitor, RLY-2608, As a Single Agent in Advanced Solid Tumor Patients and in Combination with Fulvestrant in Patients with Advanced Breast Cancer"
NCT05219513,	"A Study to Evaluate the Safety, Tolerability, Pharmacokinetics, and Efficacy of RO7443904 in Combination With Glofitamab in Participants With Relapsed/Refractory B-Cell Non-Hodgkin's Lymphoma"

NCT05224141,	"Pembrolizumab/Vibostolimab (MK-7684A) or Atezolizumab in Combination With Chemotherapy in First Line Treatment of Extensive-Stage Small Cell Lung Cancer (MK-7684A-008, KEYVIBE-008)"
NCT05226117,	"Sacituzumab Govitecan, Preceding Radical Cystectomy, in Treating Patients With Muscle-invasive Bladder Cancer"
NCT05233982,	"MITO 35a: Olaparib Maintenance Therapy in Newly Diagnosed BRCA Wild-type Advanced Ovarian, Fallopian Tube and Primitive Peritoneal Cancer"
NCT05255601,	"A Study to Evaluate the Safety, Tolerability, Drug Levels, and Preliminary Efficacy of Relatlimab Plus Nivolumab in Pediatric and Young Adults With Hodgkin and Non-Hodgkin Lymphoma"
NCT05257083,	"A Study of Daratumumab, Bortezomib, Lenalidomide and Dexamethasone (DVRd) Followed by Ciltacabtagene Autoleucel Versus Daratumumab, Bortezomib, Lenalidomide and Dexamethasone (DVRd) Followed by Autologous Stem Cell Transplant (ASCT) in Participants With Newly Diagnosed Multiple Myeloma"
NCT05257408,	"Relacorilant in Combination With Nab-Paclitaxel in Advanced, Platinum-Resistant, High-Grade Epithelial Ovarian, Primary Peritoneal, or Fallopian-Tube Cancer"
NCT05296798,	"A Study to Evaluate the Efficacy and Safety of Giredestrant in Combination With Phesgo (Pertuzumab, Trastuzumab, and Hyaluronidase-zzxf) Versus Phesgo in Participants With Locally Advanced or Metastatic Breast Cancer (heredERA Breast Cancer)"
NCT05299580,	"""Dabrafenib and Trametinib in Circulating Free DNA BRAFV600 Mutated Metastatic Melanoma Patients: a Prospective Phase II, Open Label, Multicentre Study - (Bioliquid TAILOR Study - BIOTAILOR)"""
NCT05306340,	"A Study Evaluating the Efficacy and Safety of Giredestrant Plus Everolimus Compared With the Physician's Choice of Endocrine Therapy Plus Everolimus in Participants With Estrogen Receptor-Positive, HER2-Negative, Locally Advanced or Metastatic Breast Cancer (evERA Breast Cancer)"
NCT05309668,	"Pharmacokinetics, Safety and Efficacy of the Selumetinib Granule Formulation in Children Aged ‚â•1 to <7 Years With NF1-related Symptomatic, Inoperable PN"
NCT05361395,	"First-Line Tarlatamab in Combination With Carboplatin, Etoposide, and PD-L1 Inhibitor in Subjects With Extensive Stage Small Cell Lung Cancer (ES-SCLC)"
NCT05364073,	"Study of Furmonertinib in Patients With Advanced or Metastatic Non-Small Cell Lung Cancer (NSCLC) With Activating, Including Uncommon, Epidermal Growth Factor Receptor (EGFR) or Human Epidermal Growth Factor Receptor 2 (HER2) Mutations"
NCT05364944,	"A Study to Assess the Pharmacokinetics, Pharmacodynamics, Safety, and Tolerability of Debio 4126 in Participants With Acromegaly or Functioning Gastroenteropancreatic Neuroendocrine Tumors (GEP-NETs)"
NCT05365581,	"A Study of ASP2138 Given by Itself or Given With Other Cancer Treatments in Adults With Stomach Cancer, Gastroesophageal Junction Cancer, or Pancreatic Cancer"
NCT05374512,	"A Study of Dato-DXd Versus Investigator's Choice Chemotherapy in Patients With Locally Recurrent Inoperable or Metastatic Triple-negative Breast Cancer, Who Are Not Candidates for PD-1/PD-L1 Inhibitor Therapy (TROPION-Breast02)"
NCT05382286,	"Study of Sacituzumab Govitecan-hziy and Pembrolizumab Versus Treatment of Physician's Choice and Pembrolizumab in Patients With Previously Untreated, Locally Advanced Inoperable or Metastatic Triple-Negative Breast Cancer"
NCT05386550,	"Phase III Xevinapant (Debio 1143) and Radiotherapy in Resected LA SCCHN, High Risk, Cisplatin-ineligible Participants (XRAY VISION)"
NCT05394142,	"A Clinical Trial to Evaluate the Efficacy, Tolerability, and Safety of a Fixed Dose Combination of Spironolactone, Pioglitazone & Metformin (SPIOMET) in Polycystic Ovary Syndrome (PCOS)"
NCT05406401,	"A Study of Zilovertamab Vedotin (MK-2140) in Combination With Cyclophosphamide, Doxorubicin, and Prednisone Plus Rituximab or Rituximab Biosimilar (Truxima) (R-CHP) in Participants With Diffuse Large B-Cell Lymphoma (DLBCL) (MK-2140-007)"
NCT05424380,	"A Phase 1, Open Label Study of Intravenous GSK3745417 to Evaluate Safety, Tolerability, Pharmacokinetics, Pharmacodynamics and Determine RP2D & Schedule in Participants With Relapsed or Refractory Myeloid Malignancies Including AML and HR MDS"
NCT05445778,	"Mirvetuximab Soravtansine With Bevacizumab Versus Bevacizumab as Maintenance in Platinum-sensitive Ovarian, Fallopian Tube, or Peritoneal Cancer (GLORIOSA)"
NCT05448365,	"Vitamin D, Epigallocatechin Gallate, D-chiro-inositol and Vitamin B6 in Uterine Fibroid"
NCT05495295,	"First-in-human Trial of PhOx430, a First-in-class Acetylglucosaminyltransferase V Inhibitor, in Advanced Solid Tumours"
NCT05519085,	"A Study to Evaluate Mezigdomide, Bortezomib and Dexamethasone (MEZIVd) Versus Pomalidomide, Bortezomib and Dexamethasone (PVd) in Participants With Relapsed or Refractory Multiple Myeloma (RRMM)"
NCT05535218,	"Pembrolizumab-Sacituzumab Govitecan Combination to Treat High-risk, Localized Bladder Cancer"
NCT05565378,	"A Platform Study of Novel Immunotherapy Combinations in Participants With Previously Untreated, Advanced/Metastatic Non-Small-Cell Lung Cancer"
NCT05568095,	"A Clinical Trial of a New Combination Treatment, Domvanalimab and Zimberelimab, Plus Chemotherapy, for People With an Upper Gastrointestinal Tract Cancer That Cannot be Removed With Surgery That Has Spread to Other Parts of the Body"
NCT05572515,	"A Study Comparing Teclistamab Monotherapy Versus Pomalidomide, Bortezomib, Dexamethasone (PVd) or Carfilzomib, Dexamethasone (Kd) in Participants With Relapsed or Refractory Multiple Myeloma"
NCT05578976,	"A Study to Evaluate Change in Disease Activity of Subcutaneous (SC) Epcoritamab Combined With Intravenous and Oral Rituximab, Cyclophosphamide, Doxorubicin Hydrochloride, Vincristine, and Prednisone (R-CHOP) or R-CHOP in Adult Participants With Newly Diagnosed Diffuse Large B-Cell Lymphoma (DLBCL)"
NCT05587296,	"A Study to Learn More About How Well Elinzanetant Works and How Safe it is Compared to Placebo for the Treatment of Hot Flashes Caused by Anti-cancer Therapy in Women With, or at High Risk for Developing Hormone-receptor Positive Breast Cancer"
NCT05601973,	"AMAZE-lung: Amivantamab, Lazertinib and Bevacizumab in Patients With EGFR-mutant Advanced Non-small Cell Lung Cancer With Progression on Previous Third-generation EGFR-TKI"
NCT05611931,	"Selinexor in Maintenance Therapy After Systemic Therapy for Participants with P53 Wild-Type, Advanced or Recurrent Endometrial Carcinoma"
NCT05613088,	"A Study of MORAb-202 Versus Investigator's Choice Chemotherapy in Female Participants With Platinum-resistant High-grade Serous (HGS) Ovarian, Primary Peritoneal, or Fallopian Tube Cancer"
NCT05623020,	"A Study to Learn About the Effects of the Combination of Elranatamab (PF-06863135), Daratumumab, Lenalidomide or Elranatamab and Lenalidomide Compared With Daratumumab, Lenalidomide, and Dexamethasone in Patients With Newly Diagnosed Multiple Myeloma Who Are Not Candidates for Transplant"
NCT05644561,	"Evaluation of Pharmacokinetics, Pharmacodynamics, Efficacy, Safety, and Immunogenicity of Ravulizumab Administered Intravenously in Pediatric Participants With Generalized Myasthenia Gravis (gMG)"
NCT05645692,	"A Study Evaluating Different Immunotherapies (LAG-3 and PD-1 With or Without TIGIT, Compared to PD-L1 Alone) in Participants With Untreated Locally Advanced Metastatic Urothelial Cancer"
NCT05646862,	"A Study Evaluating the Efficacy and Safety of Inavolisib Plus Fulvestrant Compared With Alpelisib Plus Fulvestrant in Participants With HR-Positive, HER2-Negative, PIK3CA Mutated, Locally Advanced or Metastatic Breast Cancer Post CDK4/6i and Endocrine Combination Therapy"
NCT05681715,	"A Phase 3, Open-label, Crossover Study to Evaluate Self-administration of Rozanolixizumab by Study Participants With Generalized Myasthenia Gravis (gMG)"
NCT05687266,	"Phase III, Open-label, First-line Study of Dato-DXd in Combination With Durvalumab and Carboplatin for Advanced NSCLC Without Actionable Genomic Alterations"
NCT05718323,	"Niraparib Added to Anti-PD-L1 Antibody Maintenance in SLFN11-positive, Extensive-disease SCLC"
NCT05730036,	"A Trial to Learn How Well Linvoseltamab Works Compared to the Combination of Elotuzumab, Pomalidomide and Dexamethasone for Adult Participants With Relapsed/Refractory Multiple Myeloma"
NCT05789082,	"A Study Evaluating the Safety, Activity, and Pharmacokinetics of Divarasib in Combination With Other Anti-Cancer Therapies in Participants With Previously Untreated Advanced or Metastatic Non-Small Cell Lung Cancer With a KRAS G12C Mutation"
NCT05808816,	"""Lactobacillus Crispatus M247, LSIL and Microbiota"""
NCT05822752,	"Study to Evaluate Adverse Events, and Change in Disease Activity, When Intravenously (IV) Infused With Livmoniplimab in Combination With IV Infused Budigalimab in Adult Participants With Hepatocellular Carcinoma (HCC)"
NCT05850520,	"A Study to Learn How Well a Higher Amount of Aflibercept Given as an Injection Into the Eye Works and How Safe it is in People With Reduced Vision Due to Swelling in the Macula, Central Part of the Retina Caused by a Blocked Vein in the Retina (Macula Edema Secondary to Retinal Vein Occlusion)"
NCT05852691,	"A Study of Tobemstomig + Nab-Paclitaxel Compared With Pembrolizumab + Nab-Paclitaxel in Participants With Previously Untreated, PD-L1-Positive, Locally-Advanced Unresectable or Metastatic Triple-Negative Breast Cancer"
NCT05867121,	"A Study to Evaluate the Safety, Pharmacokinetics, and Activity of RO7496353 in Combination With a Checkpoint Inhibitor With or Without Standard-of-Care Chemotherapy in Participants With Locally Advanced or Metastatic Solid Tumors"
NCT05904886,	"A Study Evaluating Atezolizumab and Bevacizumab, With or Without Tiragolumab, in Participants With Untreated Locally Advanced or Metastatic Hepatocellular Carcinoma (IMbrave152)"
NCT05909397,	"A Study of ARV-471 (PF-07850327) Plus Palbociclib Versus Letrozole Plus Palbociclib in Participants With Estrogen Receptor Positive, Human Epidermal Growth Factor Negative Advanced Breast Cancer"
NCT05924100,	"Efficacy and Safety of Luspatercept for the Treatment of Anemia Due to MDS With del5q, Refractory/Resistant/Intolerant to Prior Treatments, RBC-TD"
NCT05925530,	"Study to Assess Neoadjuvant Durvalumab (D) and Platinum-Based Chemotherapy (CT), Followed by Either Surgery and Adjuvant D or CRT and Consolidation D, in Resectable or Borderline Resectable Stage IIB-IIIB NSCLC (MDT-BRIDGE)"
NCT05926960,	"A Study Comparing 3 Study Medicines (Encorafenib, Binimetinib, Pembrolizumab) to 2 Study Medicines (Ipilimumab and Nivolumab) in Patients With Advanced Melanoma"
NCT05929495,	"Phase 2, Open-label, Single-arm Study on the Use of Metformin as Adjunctive Therapy in High-grade Glioma"
NCT05980598,	"TransCon (TC) TLR7/8 Agonist, TC IL-2 Œ≤/Œ≥, Pembrolizumab Prior to Surgery for Advanced Head and Neck Squamous Cell Carcinoma"
NCT06018337,	"A Study of DB-1303/BNT323 vs Investigator's Choice Chemotherapy in HER2-Low, Hormone Receptor Positive Metastatic Breast Cancer (DYNASTY-Breast02)"

NCT06047080,	"An Open-Label Study Comparing Glofitamab and Polatuzumab Vedotin + Rituximab, Cyclophosphamide, Doxorubicin, and Prednisone Versus Pola-R-CHP in Previously Untreated Patients With Large B-Cell Lymphoma"
NCT06055075,	"A Study Evaluating Safety, Tolerability, and Clinical Activity of Forimtamig-Based Treatment Combinations in Participants With Relapsed or Refractory Multiple Myeloma"
NCT06065748,	"A Study to Evaluate Efficacy and Safety of Giredestrant Compared With Fulvestrant (Plus a CDK4/6 Inhibitor), in Participants With ER-Positive, HER2-Negative Advanced Breast Cancer Resistant to Adjuvant Endocrine Therapy (pionERA Breast Cancer)"
NCT06091865,	"A Study to Compare How Well Odronextamab Combined With Chemotherapy Works and How Safe it is Against Rituximab Combined With Chemotherapy, in Patients With Previously Untreated Diffuse Large B-cell Lymphoma"
NCT06095375,	"Regorafenib With Temozolomide With or Without RT in MGMT-Methylated, IDH Wild-type GBM Patients"
NCT06109272,	"A Study to Assess the Dose, Adverse Events, and Change in Disease Activity of Livmoniplimab as an Intravenous (IV) Solution in Combination With Budigalimab as an IV Solution in Adult Participants With Hepatocellular Carcinoma (HCC)"
NCT06161025,	"A Study of Raludotatug Deruxtecan (R-DXd) in Subjects With Platinum-resistant, High-grade Ovarian, Primary Peritoneal, or Fallopian Tube Cancer"
NCT06205290,	"A Study to Compare the Efficacy and Safety of Lisocabtagene Maraleucel vs Investigator's Choice Options in Adult Participants With Relapsed or Refractory Chronic Lymphocytic Leukemia or Small Lymphocytic Lymphoma, Whose Disease Has Failed Treatment With Both BTKi and BCL2i Therapies"
NCT06208150,	"A Study Comparing Talquetamab Plus Pomalidomide, Talquetamab Plus Teclistamab, and Elotuzumab, Pomalidomide, and Dexamethasone or Pomalidomide, Bortezomib, and Dexamethasone in Participants With Relapsed or Refractory Myeloma Who Have Received an Anti-CD38 Antibody and Lenalidomide"
NCT03454750	Radiometabolic Therapy (RMT) With 177Lu PSMA 617 in Advanced Castration Resistant Prostate Cancer (CRPC)
NCT04102020	A Study of Oral Venetoclax Tablets and Oral Azacitidine as Maintenance Therapy in Adult Participants With Acute Myeloid Leukemia in First Remission After Conventional Chemotherapy
NCT04106219	A Study of LY3295668 Erbumine in Participants With Relapsed/Refractory Neuroblastoma
NCT04150029	A Study of MBG453 in Combination With Azacitidine and Venetoclax in AML Patients Unfit for Chemotherapy
NCT04162210	Study of Single Agent Belantamab Mafodotin Versus Pomalidomide Plus Low-dose Dexamethasone (Pom/Dex) in Participants With Relapsed/Refractory Multiple Myeloma (RRMM)
NCT04165083	KEYMAKER-U01 Substudy 2: Efficacy and Safety Study of Pembrolizumab (MK-3475) When Used With Investigational Agents in Treatment-naÃ¯ve Participants With Anti-programmed Cell Death Receptor Ligand 1 (PD-L1) Positive Advanced Non-small Cell Lung Cancer (NSCLC) (MK-3475-01B/KEYMAKER-U01B)
NCT04189445	Futibatinib in Patients With Specific FGFR Aberrations
NCT04208178	Study of Alpelisib (BYL719) in Combination With Trastuzumab and Pertuzumab as Maintenance Therapy in Patients With HER2-positive Advanced Breast Cancer With a PIK3CA Mutation
NCT04214288	A Study to Investigate Efficacy and Safety With Oral AZD9833 Compared With Intramuscular Fulvestrant in Post-menopausal Women at Least 18 Years of Age With Advanced ER-positive HER2 Negative Breast Cancer
NCT04221945	Study of Chemoradiotherapy With or Without Pembrolizumab (MK-3475) For The Treatment of Locally Advanced Cervical Cancer (MK-3475-A18/KEYNOTE-A18/ENGOT-cx11/GOG-3047)
NCT04222972	A Study of Pralsetinib Versus Standard of Care for First-Line Treatment of Advanced Non-Small Cell Lung Cancer (NSCLC)
NCT04223856	Enfortumab Vedotin and Pembrolizumab vs. Chemotherapy Alone in Untreated Locally Advanced or Metastatic Urothelial Cancer
NCT04245839	A Study to Evaluate the Efficacy and Safety of JCAR017 in Adult Subjects With Relapsed or Refractory Indolent B-cell Non-Hodgkin Lymphoma (NHL)
NCT04246177	Safety and Efficacy of Lenvatinib (E7080/MK-7902) With Pembrolizumab (MK-3475) in Combination With Transarterial Chemoembolization (TACE) in Participants With Incurable/Non-metastatic Hepatocellular Carcinoma (MK-7902-012/E7080-G000-318/LEAP-012)
NCT04248998	Calorie Restriction With or Without Metformin in Triple Negative Breast Cancer
NCT04251533	Study Assessing the Efficacy and Safety of Alpelisib + Nab-paclitaxel in Subjects With Advanced TNBC Who Carry Either a PIK3CA Mutation or Have PTEN Loss
NCT04259944	Post-surgical Liquid Biopsy-guided Treatment of Stage III and High-risk Stage II Colon Cancer Patients: the PEGASUS Trial
NCT04266795	A Study of Pevonedistat and Venetoclax Combined With Azacitidine to Treat Acute Myeloid Leukemia (AML) in Adults Unable to Receive Intensive Chemotherapy
NCT04294810	A Study of Tiragolumab in Combination With Atezolizumab Compared With Placebo in Combination With Atezolizumab in Patients With Previously Untreated Locally Advanced Unresectable or Metastatic PD-L1-Selected Non-Small Cell Lung Cancer
NCT04300647	A Study of Tiragolumab Plus Atezolizumab and Atezolizumab Monotherapy in Participants With Metastatic and/or Recurrent PD-L1-Positive Cervical Cancer
NCT04303169	Substudy 02C: Safety and Efficacy of Pembrolizumab in Combination With Investigational Agents or Pembrolizumab Alone in Participants With Stage III Melanoma Who Are Candidates for Neoadjuvant Therapy (MK-3475-02C/KEYMAKER-U02)
NCT04303780	""Study to Compare AMG 510 """"Proposed INN Sotorasib"""" With Docetaxel in Non Small Cell Lung Cancer (NSCLC) (CodeBreak 200).""
NCT04305041	Substudy 02A: Safety and Efficacy of Pembrolizumab in Combination With Investigational Agents in Participants With Programmed Cell-death 1 (PD-1) Refractory Melanoma (MK-3475-02A/KEYMAKER-U02)
NCT04305496	Capivasertib+Fulvestrant vs Placebo+Fulvestrant as Treatment for Locally Advanced (Inoperable) or Metastatic HR+/HER2- Breast Cancer
NCT04308590	Efficacy and Safety of Relacorilant in Patients With Cortisol-Secreting Adrenal Adenomas
NCT04322539	A Study of Efficacy and Safety of Fruquintinib (HMPL-013) in Participants With Metastatic Colorectal Cancer
NCT04338269	A Study of Atezolizumab in Combination With Cabozantinib Compared to Cabozantinib Alone in Participants With Advanced Renal Cell Carcinoma After Immune Checkpoint Inhibitor Treatment
NCT04338399	The BURAN Study of Buparlisib in Patients With Recurrent or Metastatic HNSCC
NCT04351555	A Study of Osimertinib With or Without Chemotherapy Versus Chemotherapy Alone as Neoadjuvant Therapy for Patients With EGFRm Positive Resectable Non-Small Cell Lung Cancer
NCT04362072	Study of Lorlatinib In People With ALK-positive Non-small Cell Lung Cancer
NCT04380636	Study of Pembrolizumab With Concurrent Chemoradiation Therapy Followed by Pembrolizumab With or Without Olaparib in Stage III Non-Small Cell Lung Cancer (NSCLC) (MK-7339-012/KEYLYNK-012)
NCT04398524	A Phase II Study of Cemiplimab and ISA101b in Patients With Recurrent/Metastatic HPV16 Positive OPC
NCT04401748	Study Of Venetoclax Tablet With Intravenous or Subcutaneous Azacitidine to Assess Change in Disease Activity In Adult Participants With Newly Diagnosed Higher-Risk Myelodysplastic Syndrome
NCT04402073	Personalized Risk-Adapted Therapy in Post-Pubertal Patients With Newly-Diagnosed Medulloblastoma
NCT04404283	Brentuximab Vedotin Plus Lenalidomide and Rituximab for the Treatment of Relapsed/Refractory DLBCL
NCT04411641	Nonrelapsing Secondary Progressive Multiple Sclerosis (NRSPMS) Study of Bruton's Tyrosine Kinase (BTK) Inhibitor Tolebrutinib (SAR442168)
NCT04417621	Study of Efficacy and Safety of LXH254 Combinations in Patients With Previously Treated Unresectable or Metastatic Melanoma
NCT04433182	Copanlisib With Rituximab-Bendamustine in Patients With Relapsed-Refractory Diffuse Large B-cell Lymphoma
NCT04440358	Exablate Blood-Brain Barrier Disruption With Carboplatin for the Treatment of rGBM
NCT04446117	Study of Cabozantinib in Combination With Atezolizumab Versus Second NHT in Subjects With mCRPC
NCT04447118	Phase 3 Study of Pyrotinib Versus Docetaxel in Patients With Advanced Non-squamous NSCLC Harboring a HER2 Exon 20 Mutation Who Failed Platinum Based Chemotherapy
NCT04447755	A Study of Lenvatinib (MK-7902) in Pediatric Participants With Relapsed or Refractory Solid Malignancies (MK-7902-013/E7080)
NCT04454658	Safety and Tolerability Study of Oral ABBV-744 Tablet Alone or in Combination With Oral Ruxolitinib Tablet or Oral Navitoclax Tablet in Adult Participants With Myelofibrosis
NCT04458259	Study of PF-07265807 in Participants With Metastatic Solid Tumors.
NCT04468984	Study of Oral Navitoclax Tablet in Combination With Oral Ruxolitinib Tablet to Assess Change in Spleen Volume in Adult Participants With Relapsed/Refractory Myelofibrosis
NCT04471428	Study of Atezolizumab in Combination With Cabozantinib Versus Docetaxel in Patients With Metastatic Non-Small Cell Lung Cancer Previously Treated With an Anti-PD-L1/PD-1 Antibody and Platinum-Containing Chemotherapy
NCT04472429	Carboplatin-paclitaxel With Retifanlimab or Placebo in Participants With Locally Advanced or Metastatic Squamous Cell Anal Carcinoma (POD1UM-303/InterAACT 2).
NCT04472598	Study of Oral Navitoclax Tablet In Combination With Oral Ruxolitinib Tablet When Compared With Oral Ruxolitinib Tablet To Assess Change In Spleen Volume In Adult Participants With Myelofibrosis
NCT04475939	Placebo-controlled Study Comparing Niraparib Plus Pembrolizumab Versus Placebo Plus Pembrolizumab as Maintenance Therapy in Participants With Advanced/Metastatic Non-small Cell Lung Cancer
NCT04483739	Isa-KRd vs KRd in Newly Diagnosed Multiple Myeloma Patients Eligible for Autologous Stem Cell Transplantation (IsKia TRIAL)
NCT04484142	Study of DS-1062a in Advanced or Metastatic Non-small Cell Lung Cancer With Actionable Genomic Alterations (TROPION-Lung05)
NCT04487080	A Study of Amivantamab and Lazertinib Combination Therapy Versus Osimertinib in Locally Advanced or Metastatic Non-Small Cell Lung Cancer
NCT04497844	A Study of Niraparib in Combination With Abiraterone Acetate and Prednisone Versus Abiraterone Acetate and Prednisone for the Treatment of Participants With Deleterious Germline or Somatic Homologous Recombination Repair (HRR) Gene-Mutated Metastatic Castration-Sensitive Prostate Cancer (mCSPC)
NCT04498117	Oregovomab Plus Chemo in Newly Diagnosed Patients With Advanced Epithelial Ovarian Cancer Following Optimal Debulking Surgery
NCT04504825	A Study to Evaluate the Efficacy and Safety of CAEL-101 in Patients With Mayo Stage IIIb AL Amyloidosis

NCT04512235	A Study to Evaluate the Efficacy and Safety of CAEL-101 in Patients With Mayo Stage IIIa AL Amyloidosis
NCT04513951	AVELUMAB and CETUXIMAB and mFOLFOXIRI as Initial Therapy for Unresectable Metastatic Colorectal Cancer Patients
NCT04521361	A Study to Assess How Radium-223 Distributes in the Body of Patients With Prostate Cancer Which Spread to the Bones
NCT04523493	Phase III Study of Toripalimabï¼ˆJS001ï¼‰ Combined With Lenvatinib for Advanced HCC
NCT04524273	Myasthenia Gravis Inebilizumab Trial
NCT04526691	Datopotamab Deruxtecan (Dato-DXd) in Combination With Pembrolizumab With or Without Platinum Chemotherapy in Subjects With Advanced or Metastatic Non-Small Cell Lung Cancer (TROPION-Lung02)
NCT04527991	Study of Sacituzumab Govitecan-hziy (IMMU-132) Versus Treatment of Physician's Choice in Participants With Metastatic or Locally Advanced Unresectable Urothelial Cancer
NCT04529772	A Combination of Acalabrutinib With R-CHOP in Subjects With Previously Untreated Non-GCB DLBCL (ACE-LY-312)
NCT04538742	A Phase 1b/2 Study of T-DXd Combinations in HER2-positive Metastatic Breast Cancer
NCT04543617	A Study of Atezolizumab With or Without Tiragolumab in Participants With Unresectable Esophageal Squamous Cell Carcinoma Whose Cancers Have Not Progressed Following Definitive Concurrent Chemoradiotherapy
NCT04551066	To Evaluate the Efficacy and Safety of Parsaclisib and Ruxolitinib in Participants With Myelofibrosis (LIMBER-313)
NCT04557059	A Study of Adding Apalutamide to Radiotherapy and LHRH Agonist in High-Risk Patients With Hormone-Sensitive Prostate Cancer
NCT04557098	A Study of Teclistamab in Participants With Relapsed or Refractory Multiple Myeloma
NCT04564703	Iberdomide (Cc220) Maintenance After Asct in Newly Diagnosed MM Patients
NCT04569032	A Study of Brentuximab Vedotin and CHP in Frontline Treatment of PTCL With Less Than 10% CD30 Expression
NCT04570631	Study to Determine Recommended Phase 2 Dose of Intravenous (IV) Eftozanermin Alfa in Combination With IV or Subcutaneous (SC) Bortezomib and Oral Dexamethasone Tablet and to Assess Change in Disease Symptoms in Adult Participants With Relapsed or Refractory Multiple Myeloma
NCT04579380	Basket Study of Tucatinib and Trastuzumab in Solid Tumors With HER2 Alterations
NCT04579679	Open-Label Surufatinib in European Patients With NET
NCT04581824	Efficacy Comparison of Dostarlimab Plus Chemotherapy Versus Pembrolizumab Plus Chemotherapy in Participants With Metastatic Non-squamous Non-small Cell Lung Cancer (NSCLC)
NCT04586231	A Study of Belzutifan (MK-6482) in Combination With Lenvatinib Versus Cabozantinib for Treatment of Renal Cell Carcinoma (MK-6482-011)
NCT04590963	Assessment of Efficacy and Safety of Monalizumab Plus Cetuximab Compared to Placebo Plus Cetuximab in Recurrent or Metastatic Head and Neck Cancer
NCT04591431	The Rome Trial From Histology to Target: the Road to Personalize Target Therapy and Immunotherapy
NCT04603495	Phase 3 Study of Pelabresib (CPI-0610) in Myelofibrosis (MF) (MANIFEST-2)
NCT04607421	A Study of Encorafenib Plus Cetuximab With or Without Chemotherapy in People With Previously Untreated Metastatic Colorectal Cancer
NCT04608318	Ibrutinib Monotherapy Versus Fixed-duration Venetoclax Plus Obinutuzumab Versus Fixed-duration Ibrutinib Plus Venetoclax in Patients With Previously Untreated Chronic Lymphocytic Leukaemia (CLL)
NCT04619004	HERTHENA-Lung01: Patritumab Deruxtecan in Subjects With Metastatic or Locally Advanced EGFR-mutated Non-Small Cell Lung Cancer
NCT04619797	A Study of Tiragolumab in Combination With Atezolizumab Plus Pemetrexed and Carboplatin/Cisplatin Versus Pembrolizumab Plus Pemetrexed and Carboplatin/Cisplatin in Participants With Previously Untreated Advanced Non-Squamous Non-Small Cell Lung Cancer
NCT04622319	A Study of Trastuzumab Deruxtecan (T-DXd) Versus Trastuzumab Emtansine (T-DM1) in High-risk HER2-positive Participants With Residual Invasive Breast Cancer Following Neoadjuvant Therapy (DESTINY-Breast05)
NCT04623216	Sabatolimab as a Treatment for Patients With Acute Myeloid Leukemia and Presence of Measurable Residual Disease After Allogeneic Stem Cell Transplantation.
NCT04623775	A Study of Relatlimab Plus Nivolumab in Combination With Chemotherapy vs. Nivolumab in Combination With Chemotherapy as First Line Treatment for Participants With Stage IV or Recurrent Non-small Cell Lung Cancer (NSCLC)
NCT04625270	A Study of Avutometinib (VS-6766) v. Avutometinib (VS-6766) + Defactinib in Recurrent Low-Grade Serous Ovarian Cancer With and Without a KRAS Mutation
NCT04628494	A Phase 3 Trial of Epcoritamab vs Investigator's Choice Chemotherapy in R/R DLBCL
NCT04632433	Neoadjuvant Plus Adjuvant Treatment With Cemiplimab in Cutaneaous Squamous Cell Carcinoma
NCT04634877	Study of Pembrolizumab (MK-3475) in Combination With Adjuvant Chemotherapy With or Without Radiotherapy in Participants With Newly Diagnosed Endometrial Cancer After Surgery With Curative Intent (MK-3475-B21 / KEYNOTE-B21 / ENGOT-en11 / GOG-3053)
NCT04639219	A Study of T-DXd for the Treatment of Solid Tumors Harboring HER2 Activating Mutations
NCT04641247	A Long-term Treatment Extension Study of Niraparib in Participants Who Completed a Prior GlaxoSmithKline/TESARO-sponsored Niraparib Study
NCT04644237	Trastuzumab Deruxtecan in Participants With HER2-mutated Metastatic Non-small Cell Lung Cancer (NSCLC)
NCT04646005	Cemiplimab and ISA101b Vaccine in Adult Participants With Recurrent/Metastatic Human Papillomavirus (HPV)16 Cervical Cancer Who Have Experienced Disease Progression After First Line Chemotherapy
NCT04646395	Study of Acalabrutinib and Tafasitamab in MZL Patients
NCT04655976	Efficacy Comparison of Cobolimab + Dostarlimab + Docetaxel to Dostarlimab + Docetaxel to Docetaxel Alone in Participants With Advanced Non-Small Cell Lung Cancer Who Have Progressed on Prior Anti- Programmed Death-ligand 1 (PD-[L]1) Therapy and Chemotherapy
NCT04656652	Study of DS-1062a Versus Docetaxel in Previously Treated Advanced or Metastatic Non-small Cell Lung Cancer With or Without Actionable Genomic Alterations (TROPION-LUNG01)
NCT04660812	An Open Label Study Evaluating the Efficacy and Safety of Etrumadenant (AB928) Based Treatment Combinations in Participants With Metastatic Colorectal Cancer.
NCT04662255	Study of BTK Inhibitor LOXO-305 Versus Approved BTK Inhibitor Drugs in Patients With Mantle Cell Lymphoma (MCL)
NCT04662710	Efficacy and Safety of Lenvatinib (E7080/MK-7902) Plus Pembrolizumab (MK-3475) Plus Chemotherapy in Participants With Advanced/Metastatic Gastroesophageal Adenocarcinoma (MK-7902-015/E7080-G000-321/LEAP-015)
NCT04665843	A Study of Atezolizumab Plus Tiragolumab and Atezolizumab Plus Placebo as First-Line Treatment in Participants With Recurrent/Metastatic PD-L1 Positive Squamous Cell Carcinoma of the Head and Neck
NCT04666038	Study of LOXO-305 Versus Investigator's Choice (IdelaR or BR) in Patients With Previously Treated Chronic Lymphocytic Leukemia (CLL)/Small Lymphocytic Lymphoma (SLL)
NCT04669067	TL-895 and KRT-232 Study in Acute Myeloid Leukemia
NCT04674683	Study Comparing Investigational Drug HBI-8000 + Nivolumab vs. Placebo + Nivolumab in Patients With Advanced Melanoma
NCT04680052	A Phase 3 Study to Assess Efficacy and Safety of Tafasitamab Plus Lenalidomide and Rituximab Compared to Placebo Plus Lenalidomide and Rituximab in Patients With Relapsed/Refractory (R/R) Follicular Lymphoma or Marginal Zone Lymphoma.
NCT04685135	Phase 3 Study of MRTX849 (Adagrasib) vs Docetaxel in Patients With Advanced Non-Small Cell Lung Cancer With KRAS G12C Mutation
NCT04692740	Chlorambucil in Metastatic PDAC Patients Bearing a Germ Line DNA Defects Repair Mutations (SALE Trial)
NCT04697628	Tisotumab Vedotin vs Chemotherapy in Recurrent or Metastatic Cervical Cancer
NCT04700072	Substudy 02D: Safety and Efficacy of Pembrolizumab in Combination With Investigational Agents or Pembrolizumab Alone in Participants With Melanoma Brain Metastasis (MK-3475-02D/KEYMAKER-U02)
NCT04700124	Perioperative Enfortumab Vedotin (EV) Plus Pembrolizumab (MK-3475) Versus Neoadjuvant Chemotherapy for Cisplatin-eligible Muscle Invasive Bladder Cancer (MIBC) (MK-3475-B15/ KEYNOTE-B15 / EV-304)
NCT04704219	Pembrolizumab Plus Lenvatinib for First-line Advanced/Metastatic Non-clear Cell Renal Cell Carcinoma (1L nccRCC) (MK-3475-B61)
NCT04711252	A Comparative Study of AZD9833 Plus Palbociclib Versus Anastrozole Plus Palbociclib in Patients With ER-Positive HER2 Negative Breast Cancer Who Have Not Received Any Systemic Treatment for Advanced Disease
NCT04717414	An Efficacy and Safety Study of Luspatercept (ACE-536) Versus Placebo in Subjects With Myeloproliferative Neoplasm-Associated Myelofibrosis on Concomitant JAK2 Inhibitor Therapy and Who Require Red Blood Cell Transfusions
NCT04733118	Chemotherapy-Free pCR-Guided Strategy With Trastuzumab-pertuzumab and T-DM1 in HER2-positive Early Breast Cancer
NCT04739761	A Study of T-DXd in Participants With or Without Brain Metastasis Who Have Previously Treated Advanced or Metastatic HER2 Positive Breast Cancer
NCT04740307	Safety and Efficacy of Coformulated Pembrolizumab/Quavonlimab (MK-1308A) in Combination With Lenvatinib (E7080/MK-7902) in Advanced Hepatocellular Carcinoma (MK-1308A-004)
NCT04744831	Trastuzumab Deruxtecan in Participants With HER2-overexpressing Advanced or Metastatic Colorectal Cancer
NCT04745234	Mogamulizumab Q4week Dosing in Participants With R/R CTCL
NCT04758507	Fecal Microbiota Transplantation to Improve Efficacy of Immune Checkpoint Inhibitors in Renal Cell Carcinoma
NCT04762069	A Study of Berubicin in Adult Subjects With Recurrent Glioblastoma Multiforme
NCT04765059	A Study to Evaluate Chemotherapy Plus Osimertinib Against Chemotherapy Plus Placebo in Patients With Non-small Cell Lung Cancer (NSCLC)
NCT04765709	Durvalumab and Chemotherapy Induction Followed by Durvalumab and Radiotherapy in Large Volume Stage III NSCLC
NCT04770545	An Extension Study to Evaluate the Long-term Safety and Efficacy of Pegcetacoplan (APL-2) in Subjects With Geographic Atrophy Secondary to AMD
NCT04770896	A Study of Atezolizumab With Lenvatinib or Sorafenib Versus Lenvatinib or Sorafenib Alone in Hepatocellular Carcinoma Previously Treated With Atezolizumab and Bevacizumab
NCT04774380	Study of Durvalumab in Combination With Platinum and Etoposide for the First Line Treatment of Patients With Extensive-stage Small Cell Lung Cancer
NCT04785820	A Study of Lomvastomig (RO7121661) and Tobemstomig (RO7247669) Compared With Nivolumab in Participants With Advanced or Metastatic Squamous Cell Carcinoma of the Esophagus
NCT04793958	Phase 3 Study of MRTX849 With Cetuximab vs Chemotherapy in Patients With Advanced Colorectal Cancer With KRAS G12C Mutation (KRYSTAL-10)

NCT04817007	A Study to Assess the Safety and Tolerability of BMS-986158 Alone and in Combination With Either Ruxolitinib or Fedratinib in Participants With Blood Cancer (Myelofibrosis)
NCT04818671	Evaluating the Long-Term Safety and Tolerability of Efgartigimod PH20 SC Administered Subcutaneously in Patients With Generalized Myasthenia Gravis
NCT04821622	Study of Talazoparib With Enzalutamide in Men With DDR Gene Mutated mCSPC
NCT04824092	Tafasitamab + Lenalidomide + R-CHOP Versus R-CHOP in Newly Diagnosed High-intermediate and High Risk DLBCL Patients
NCT04844866	Efficacy and Safety of MB-CART2019.1 vs. SoC in Lymphoma Patients
NCT04854499	Study of Magrolimab Combination Therapy in Patients With Head and Neck Squamous Cell Carcinoma
NCT04871282	A Study of AL102 in Patients With Progressing Desmoid Tumors
NCT04875195	A Study of Pembrolizumab (MK-3475) in Relapsed or Refractory Classical Hodgkin's Lymphoma (rrcHL) or Relapsed or Refractory Primary Mediastinal Large B-cell Lymphoma (rrPMBCL) (MK-3475-B68)
NCT04879368	RegoNivo vs Standard of Care Chemotherapy in AGOC
NCT04895358	Study of Pembrolizumab (MK-3475) Plus Chemotherapy Versus Placebo Plus Chemotherapy for HR+/HER2- Locally Recurrent Inoperable or Metastatic Breast Cancer (MK-3475-B49/KEYNOTE-B49)
NCT04895722	Evaluation of Co-formulated Pembrolizumab/Quavonlimab (MK-1308A) Versus Other Treatments in Participants With Microsatellite Instability-High (MSI-H) or Mismatch Repair Deficient (dMMR) Stage IV Colorectal Cancer (CRC) (MK-1308A-008/KEYSTEP-008)
NCT04895748	DFF332 as a Single Agent and in Combination With Everolimus & Immuno-Oncology Agents in Advanced/Relapsed Renal Cancer & Other Malignancies
NCT04895917	Daratumumab and Pomalidomide in Previously Treated Patients With AL Amyloidosis
NCT04913220	A Study of SAR444245 Combined With Cemiplimab for the Treatment of Participants With Various Advanced Skin Cancers (Pegathor Skin 201)
NCT04914897	A Study of SAR444245 Combined With Other Anticancer Therapies for the Treatment of Participants With Lung Cancer or Mesothelioma (Pegathor Lung 202)
NCT04915755	Efficacy and Safety Comparison of Niraparib to Placebo in Participants With Human Epidermal Growth Factor 2 Negative (HER2-) Breast Cancer Susceptibility Gene Mutation (BRCAmut) or Triple-Negative Breast Cancer (TNBC) With Molecular Disease
NCT04924101	Efficacy and Safety of Pembrolizumab Plus Investigational Agents in Combination With Chemotherapy as First-Line Treatment in Extensive-Stage Small Cell Lung Cancer (ES-SCLC) (MK-3475-B99/ KEYNOTE-B99)
NCT04925284	Study of XB002 in Subjects With Solid Tumors (JEWEL-101)
NCT04929223	A Study Evaluating the Safety and Efficacy of Targeted Therapies in Subpopulations of Patients With Metastatic Colorectal Cancer (INTRINSIC)
NCT04931342	A Study Evaluating the Efficacy and Safety of Biomarker-Driven Therapies in Patients With Persistent or Recurrent Rare Epithelial Ovarian Tumors
NCT04933695	A Study of Sotorasib (AMG 510) in Participants With Stage IV NSCLC Whose Tumors Harbor a KRAS p.G12C Mutation in Need of First-line Treatment
NCT04935359	Study of Efficacy and Safety of NIS793 in Combination With Standard of Care (SOC) Chemotherapy in First-line Metastatic Pancreatic Ductal Adenocarcinoma (mPDAC) - daNIS-2
NCT04938817	Safety and Efficacy Study of Pembrolizumab (MK-3475) in Combination With Investigational Agents for the Treatment of Extensive-Stage Small Cell Lung Cancer (ES-SCLC) in Need of Second-Line Therapy (MK-3475-B98/KEYNOTE-B98)
NCT04948333	Asciminib Treatment Optimization in â‰¥ 3rd Line CML-CP.
NCT04952753	Study of NIS793 and Other Novel Investigational Combinations With SOC Anti-cancer Therapy for the 2L Treatment of mCRC
NCT04965155	A Trial for Relapsed Multiple Myeloma Patients (Isatuximab-dexamethasone)
NCT04971226	A Study of Oral Asciminib Versus Other TKIs in Adult Patients With Newly Diagnosed Ph+ CML-CP
NCT04980495	An Open-label Study to Investigate the Clinical Efficacy of Different Dosing Regimens of Efgartigimod IV in Patients With Generalized Myasthenia Gravis
NCT04980872	A Study of the Safety and Tolerability in Participants With PIK3CA-related Overgrowth Spectrum or Proteus Syndrome Who Are Being Treated With Miransertib (MK-7075) in Other Studies (MK-7075-006)
NCT04987203	Study to Compare Tivozanib in Combination With Nivolumab to Tivozanib Monotherapy in Subjects With Renal Cell Carcinoma
NCT04988295	A Study of Amivantamab and Lazertinib in Combination With Platinum-Based Chemotherapy Compared With Platinum-Based Chemotherapy in Patients With Epidermal Growth Factor Receptor (EGFR)-Mutated Locally Advanced or Metastatic Non- Small Cell Lung Cancer After Osimertinib Failure
NCT05002569	A Study to Assess Adjuvant Immunotherapy With Nivolumab Plus Relatlimab Versus Nivolumab Alone After Complete Resection of Stage III-IV Melanoma
NCT05007106	MK-7684A With or Without Other Anticancer Therapies in Participants With Selected Solid Tumors (MK-7684A-005) (KEYVIBE-005)
NCT05015010	Alectinib in Neo-adjuvant Treatment of Stage III NSCLC
NCT05023980	A Study of Pirtobrutinib (LOXO-305) Versus Bendamustine Plus Rituximab (BR) in Untreated Patients With Chronic Lymphocytic Leukemia (CLL)/Small Lymphocytic Lymphoma (SLL)
NCT05024045	Study of Oral LOXO-338 in Patients With Advanced Blood Cancers
NCT05050942	A Trial to Assess Efficacy and Safety of Octreotide Subcutaneous Depot in Patients With GEP-NET
NCT05052801	Bemarituzumab or Placebo Plus Chemotherapy in Gastric Cancers With Fibroblast Growth Factor Receptor 2b (FGFR2b) Overexpression
NCT05054725	Combination Study of RMC-4630 and Sotorasib for NSCLC Subjects With KRASG12C Mutation After Failure of Prior Standard Therapies
NCT05059262	Study of Vimseltinib for Tenosynovial Giant Cell Tumor
NCT05059522	Continued Access Study for Participants Deriving Benefit in Pfizer-Sponsored Avelumab Parent Studies That Are Closing
NCT05060016	A Phase 2 Study of Tarlatamab in Patients With Small Cell Lung Cancer (SCLC)
NCT05060432	Study of EOS-448 With Standard of Care and/or Investigational Therapies in Participants With Advanced Solid Tumors
NCT05061134	A Study of Ceralasertib Monotherapy and Ceralasertib Plus Durvalumab in Patients With Melanoma and Resistance to PD-(L)1 Inhibition
NCT05061420	A Study of SAR444245 Combined With Other Anticancer Therapies for the Treatment of Participants With HNSCC (Master Protocol) (Pegathor Head and Neck 204)
NCT05061823	Bintrafusp Alfa Program Rollover Study
NCT05063786	Trastuzumab + Alpelisib +/- Fulvestrant vs Trastuzumab + CT in Patients With PIK3CA Mutated Previously Treated HER2+ Advanced BrEasT Cancer (ALPHABET)
NCT05064059	A Study of Coformulated Favezelimab/Pembrolizumab (MK-4280A) Versus Standard of Care in Subjects With Previously Treated Metastatic PD-L1 Positive Colorectal Cancer (MK-4280A-007)
NCT05064358	Study to Investigate Alternative Dosing Regimens of Belantamab Mafodotin in Participants With Relapsed or Refractory Multiple Myeloma
NCT05089734	Study of Sacituzumab Govitecan (SG) Versus Docetaxel in Participants With Advanced or Metastatic Non-Small Cell Lung Cancer (NSCLC)
NCT05092360	Phase 3 Study of Nemvaleukin Alfa in Combination With Pembrolizumab in Patients With Platinum-Resistant Epithelial Ovarian Cancer (ARTISTRY-7)
NCT05104567	A Study of SAR444245 Combined With Other Anticancer Therapies for the Treatment of Participants With Gastrointestinal Cancer (Master Protocol) (Pegathor Gastrointestinal 203)
NCT05116189	Pembrolizumab/Placebo Plus Paclitaxel With or Without Bevacizumab for Platinum-resistant Recurrent Ovarian Cancer (MK-3475-B96/KEYNOTE-B96/ENGOT-ov65)
NCT05117242	Safety and Efficacy Study of GEN1046 as a Single Agent or in Combination With Pembrolizumab for Treatment of Recurrent (Non-small Cell) Lung Cancer
NCT05132075	Study of JDQ443 in Comparison With Docetaxel in Participants With Locally Advanced or Metastatic KRAS G12C Mutant Non-small Cell Lung Cancer
NCT05132582	A Study of Tucatinib or Placebo With Trastuzumab and Pertuzumab for Metastatic HER2+ Breast Cancer
NCT05144841	A Study to Evaluate Zilovertamab Vedotin (MK-2140) for Relapsed or Refractory Diffuse Large B-Cell Lymphoma (DLBCL) (MK-2140-004)
NCT05155254	IO102-IO103 in Combination With Pembrolizumab Versus Pembrolizumab Alone in Advanced Melanoma (IOB-013 / KN-D18)
NCT05160922	Crizotinib Continuation Clinical Study
NCT05168202	A Study to Assess the Effect of CC-95251 in Participants With Acute Myeloid Leukemia and Myelodysplastic Syndromes
NCT05169567	Abemaciclib (LY2835219) Plus Fulvestrant Compared to Placebo Plus Fulvestrant in Previously Treated Breast Cancer
NCT05171777	A Study to Evaluate Participant and Healthcare Professional Reported Preference for Subcutaneous Atezolizumab Compared With Intravenous Atezolizumab Formulation in Participants With Non-Small Cell Lung Cancer
NCT05172596	PHE885 CAR-T Therapy in Adult Participants With Relapsed and Refractory Multiple Myeloma
NCT05173987	Study of Pembrolizumab (MK-3475) Versus Chemotherapy in Mismatch Repair Deficient (dMMR) Advanced or Recurrent Endometrial Carcinoma (MK-3475-C93/KEYNOTE-C93/GOG-3064/ENGOT-en15)
NCT05186974	Study of Sacituzumab Govitecan Combinations in First-line Treatment of Participants With Advanced or Metastatic Non-Small-Cell Lung Cancer (NSCLC)
NCT05198934	Sotorasib and Panitumumab Versus Investigator's Choice for Participants With Kirsten Rat Sarcoma (KRAS) p.G12C Mutation
NCT05199337	Phase 1/2 Study of ZN-d5 for the Treatment of Relapsed or Refractory Light Chain (AL) Amyloidosis
NCT05204927	177Lu-PSMA-I&T for Metastatic Castration-Resistant Prostate Cancer
NCT05210790	A Phase 3 Study of Rusfertide in Patients With Polycythemia Vera
NCT05216835	Safety and Preliminary Efficacy Assessment of AZD7789 in Patients With Relapsed or Refractory Classical Hodgkin Lymphoma
NCT05218499	Brightline-1: A Study to Compare Brigimadlin (BI 907828) With Doxorubicin in People With a Type of Cancer Called Dedifferentiated Liposarcoma
NCT05222555	Safety and Pharmacokinetics Study of a Modified Tafasitamab IV Dosing Regimen Combined With Lenalidomide in R-R DLBCL Patients
NCT05223920	Extension Study of Bomedemstat (IMG-7289/MK-3543) in Participants With Myeloproliferative Neoplasms (IMG-7289-CTP-202/MK-3543-005)
NCT05239728	A Study of Belzutifan (MK-6482) Plus Pembrolizumab (MK-3475) Versus Placebo Plus Pembrolizumab in Participants With Clear Cell Renal Cell Carcinoma Post Nephrectomy (MK-6482-022)
NCT05245071	Tusamitamab Ravtansine in NSQ NSCLC Participants With Negative or Moderate CEACAM5 Expression Tumors and High Circulating CEA
NCT05253846	Short-course Radiotherapy Followed by Consolidation Chemotherapy. 2021-001206-29

NCT05254743	A Study of Pirtobrutinib (LOXO-305) Versus Ibrutinib in Participants With Chronic Lymphocytic Leukemia (CLL)/Small Lymphocytic Lymphoma (SLL)
NCT05256381	A Study of SOT101 in Combination With Pembrolizumab to Evaluate the Efficacy and Safety in Patients With Selected Advanced Solid Tumors
NCT05266937	Atezolizumab Plus CArboplatin Plus Nab-paclitaxel
NCT05267106	Study to Evaluate the Efficacy and Safety of Pemigatinib in Participants With Previously Treated Glioblastoma or Other Primary Central Nervous System Tumors Harboring Activating FGFR1-3 Alterations
NCT05269355	A Study of Unesbulin in Participants With Advanced Leiomyosarcoma (LMS)
NCT05270044	Adjuvant Encorafenib and Binimetinib in High-risk Stage II Melanoma With a BRAF Mutation.
NCT05287113	Study of Retinfanlimab in Combination With INCAGN02385 and INCAGN02390 as First-Line Treatment in Participants With PD-L1-Positive (CPS â‰¥ 1) Recurrent/Metastatic Squamous Cell Carcinoma of the Head and Neck
NCT05288166	A Study of Abemaciclib (LY2835219) With Abiraterone in Men With Prostate Cancer That Has Spread to Other Parts of the Body and is Expected to Respond to Hormonal Treatment (Metastatic Hormone-Sensitive Prostate Cancer)
NCT05312398	CAPRI 2 GOIM Study: Investigate the Efficacy and Safety of a Bio-marker Driven Cetuximab-based Treatment Regimen
NCT05323045	A First-in-human Dose-escalation and Expansion Study With the Antibody-drug Conjugate BYON3521
NCT05323656	A Study of Setanaxib Co-Administered With Pembrolizumab in Patients With Recurrent or Metastatic Squamous Cell Carcinoma of Head and Neck (SCCHN)
NCT05323734	Adjunctive GNX Treatment Compared With Placebo in Children and Adults With TSC-related Epilepsy
NCT05327530	A Study of the Safety and Efficacy of Various Combinations of Avelumab as Therapy in Locally Advanced or Metastatic Urothelial Carcinoma (JAVELIN Bladder Medley)
NCT05328908	A Study of Nivolumab-relatlimab Fixed-dose Combination Versus Regorafenib or TAS-102 in Participants With Later-lines of Metastatic Colorectal Cancer
NCT05338970	HERTHENA-Lung02: A Study of Patritumab Deruxtecan Versus Platinum-based Chemotherapy in Metastatic or Locally Advanced EGFRm NSCLC After Failure of EGFR TKI Therapy
NCT05342636	A Study of Combination Therapies With or Without Pembrolizumab (MK-3475) and/or Chemotherapy in Participants With Advanced Esophageal Cancer (MK-3475-06A)
NCT05358249	Platform Study of JDQ443 in Combinations in Patients With Advanced Solid Tumors Harboring the KRAS G12C Mutation
NCT05383170	A Study to Evaluate the Safety and Efficacy of CyPep-1 in Combination With Pembrolizumab for the Treatment of Advanced or Metastatic Cancers
NCT05383352	A Study to Compare Onivyde Manufactured at Two Different Production Sites in Adult Participants With Advanced Cancer in the Pancreas
NCT05388669	A Study of Lazertinib With Subcutaneous Amivantamab Compared With Intravenous Amivantamab in Participants With Epidermal Growth Factor Receptor (EGFR)-Mutated Advanced or Metastatic Non-small Cell Lung Cancer
NCT05403450	A Study of Tolinapant in Combination With Oral Decitabine/Cedazuridine and Oral Decitabine/Cedazuridine Alone in Participants With Relapsed/Refractory Peripheral T-cell Lymphoma (R/R PTCL)
NCT05403541	Phase 3 Study to Assess the Efficacy and Safety of Batoclimab as Induction and Maintenance Therapy in Adult Participants With Generalized Myasthenia Gravis
NCT05405166	SC Versus IV Isatuximab in Combination With Pomalidomide and Dexamethasone in RRMM
NCT05445843	Study of Efficacy and Safety of JDQ443 Single-agent as First-line Treatment for Patients With Locally Advanced or Metastatic KRAS G12C- Mutated Non-small Cell Lung Cancer With a PD-L1 Expression < 1% or a PD-L1 Expression â‰¥ 1% and an STK11 Co-mutation.
NCT05446870	Pembrolizumab With Chemotherapy and MK-4830 for Treating Participants With Ovarian Cancer (MK-4830-002)
NCT05450692	A Phase III Study of Ceralasertib Plus Durvalumab Versus Docetaxel in Patients With Non Small Cell Lung Cancer (NSCLC) Whose Disease Progressed On or After Prior Anti PD (L)1 Therapy And Platinum Based Chemotherapy
NCT05462717	Dose Escalation and Dose Expansion Study of RMC-6291 Monotherapy in Subjects With Advanced KRASG12C Mutant Solid Tumors
NCT05469737	A Study to Compare the Efficacy and Safety of Oral Azacitidine Plus Best Supportive Care (BSC) Versus Placebo Plus BSC in Participants With International Prognostic Scoring System Revised (IPSS-R) Low- or Intermediate-risk Myelodysplastic Syndrome (MDS)
NCT05488314	A Study of Amivantamab and Capmatinib Combination Therapy in Unresectable Metastatic Non-small Cell Lung Cancer
NCT05490446	A Study of Tebapivat (AG-946) in Participants With Anemia Due to Lower-Risk Myelodysplastic Syndromes (LR-MDS)
NCT05498155	Study of Neoadjuvant Olaparib Monotherapy and Olaparib and Durvalumab Combination in HER2 Negative BRCAm Breast Cancer
NCT05502237	Zimberelimab and Domvanalimab in Combination With Chemotherapy Versus Pembrolizumab With Chemotherapy in Patients With Untreated Metastatic Non-Small Cell Lung Cancer
NCT05513703	A Study to Assess Disease Activity of Intravenously (IV) Infused Telisotuzumab Vedotin in Adult Participants With Advanced/Metastatic Non-Squamous Non-Small Cell Lung Cancer (NSCLC)
NCT05537766	Study of Brexucabtagene Autoleucel in Adults With Rare B-cell Malignancies
NCT05543629	A Study of BMS-986442 With Nivolumab With or Without Chemotherapy in Solid Tumors and Non-small Cell Lung Cancer
NCT05551117	A Study of Vobramitamab Duocarmazine in Participants With Metastatic Castration Resistant Prostate Cancer and Other Solid Tumors
NCT05593614	Efficacy and Safety of ATX01 in Adult Patients With CIPN (Chemotherapy-induced Peripheral Neuropathy)
NCT05608044	A Study of Botensilimab and Balstilimab for the Treatment of Colorectal Cancer
NCT05622162	Prospective Comparative Study for Patients With Biochemical Recurrence Prostate Cancer Detecting by 18F-JK-PSMA-7
NCT05665595	A Study of Adjuvant Pembrolizumab/Vibostolimab (MK-7684A) Versus Pembrolizumab for Resected High-Risk Melanoma in Participants With High-Risk Stage II-IV Melanoma (MK-7684A-010/KEYVIBE-010)
NCT05678257	A Study of NUC-3373 in Combination With Other Agents in Patients With Colorectal Cancer
NCT05740566	Study Comparing Tarlatamab With Standard of Care Chemotherapy in Relapsed Small Cell Lung Cancer
NCT05775289	A Study of Tobemstomig Plus Platinum-Based Chemotherapy vs Pembrolizumab Plus Platinum-Based Chemotherapy in Participants With Previously Untreated Non-Small Cell Lung Cancer
NCT05804045	Study of Pimicotinib (ABSK021) for Tenosynovial Giant Cell Tumor (MANEUVER)
NCT05827016	A Study to Compare Iberdomide Maintenance Versus Lenalidomide Maintenance Therapy Following Autologous Stem Cell Transplant in Participants With Newly Diagnosed Multiple Myeloma
NCT05840211	Study of Sacituzumab Govitecan Versus Treatment of Physician's Choice in Patients With Hormone Receptor-positive/Human Epidermal Growth Factor Receptor 2 Negative (HR+/HER2-) Metastatic Breast Cancer Who Have Received Endocrine Therapy
NCT05845814	A Study of Efficacy and Safety of Pembrolizumab Plus Enfortumab Vedotin (EV) +/- Investigational Agents in First-Line Metastatic Urothelial Carcinoma (mUC) (MK-3475-04B/KEYMAKER-U04)
NCT05846594	A Study to Evaluate the Impact of Liquid Biopsy in Participants With a Clinical Diagnosis of Advanced Cancer
NCT06039384	A Study of INCB099280 in Combination With Adagrasib in Adults With Advanced Solid Tumors Harboring a KRASG12C Mutation
NCT04249362	Study of Durvalumab Following Radiation Therapy in Patients With Stage 3 Unresectable NSCLC Ineligible for Chemotherapy
NCT04293094	Study of AMG 650 in Adult Participants With Advanced Solid Tumors
NCT04350463	A Safety and Efficacy Study of CC-90011 in Combination With Nivolumab in Subjects With Advanced Cancers
NCT04385368	Phase III Study to Determine the Efficacy of Durvalumab in Combination With Chemotherapy in Completely Resected Stage II-III Non-small Cell Lung Cancer (NSCLC)
NCT04385992	Neoadjuvant PRRT With 177Lu-DOTATATE Followed by Surgery for Resectable PanNET
NCT04393753	Domatinostat in Combination With Avelumab in Patients With Advanced Merkel Cell Carcinoma Progressing on Anti-PD-(L)1
NCT04427072	Study of Capmatinib Efficacy in Comparison With Docetaxel in Previously Treated Participants With Non-small Cell Lung Cancer Harboring MET Exon 14 Skipping Mutation
NCT04466891	A Study of ZW25 (Zanidatamab) in Subjects With Advanced or Metastatic HER2-Amplified Biliary Tract Cancers
NCT04487067	A Study of Atezolizumab (Tecentriq) in Combination With Bevacizumab to Investigate Safety and Efficacy in Patients With Unresectable Hepatocellular Carcinoma Not Previously Treated With Systemic Therapy-Amethista
NCT04495621	MEN1611 With Cetuximab in Metastatic Colorectal Cancer (C-PRECISE-01)
NCT04524442	Post-Authorization Safety Study (PASS) of LysaKareÂ® in Adult Gastroenteropancreatic Neuroendocrine Tumor (GEP-NET) Patients
NCT04524455	Blinatumomab in Combination With AMG 404 for the Treatment of Adults With Relapsed or Refractory B Cell Precursor ALL
NCT04526704	Study to Evaluate Discontinuation and Re-Treatment in Participants With Tenosynovial Giant Cell Tumor (TGCT) Previously Treated With Pexidartinib
NCT04580485	INCB106385 Alone or in Combination With Immunotherapy in Advanced Solid Tumors
NCT04582539	To Assess the Safety and Tolerability of INCB000928 in Participants With Myelodysplastic Syndromes or Multiple Myeloma
NCT04590248	A Study of Adavosertib as Treatment for Uterine Serous Carcinoma
NCT04622774	First-in-Human Study of IMGC936 in Patients With Advanced Solid Tumors
NCT04627896	Targeted Microwave Focal Therapy
NCT04642469	Phase III Study to Determine Efficacy of Durvalumab in Stage II-III Non-small Cell Lung Cancer (NSCLC) After Curative Intent Therapy.
NCT04650854	A Study to Evaluate Rozanolixizumab in Study Participants With Generalized Myasthenia Gravis
NCT04676334	CATCH-R: A Rollover Study to Provide Continued Access to Rucaparib
NCT04696055	Regorafenib Plus Pembrolizumab in Patients With Advanced or Spreading Liver Cancer Who Have Been Previously Treated With PD-1/PD-L1 Immune Checkpoint Inhibitors
NCT04704154	A Trial to Learn Whether Regorafenib in Combination With Nivolumab Can Improve Tumor Responses and How Safe it is for Participants With Solid Tumors
NCT04730999	Efficacy and Safety Study of a New Therapeutic Strategy in the Treatment of Extended-Disease Small-Cell Lung Cancer
NCT04735432	Evaluating the Pharmacodynamic Noninferiority of Efgartigimod PH20 SC Administered Subcutaneously as Compared to Efgartigimod Administered Intravenously in Patients With Generalized Myasthenia Gravis

NCT04737187	Phase III Study of Trifluridine/Tipiracil With and Without Bevacizumab in Refractory Metastatic Colorectal Cancer Patients
NCT04740918	A Study of Trastuzumab Emtansine in Combination With Atezolizumab or Placebo as a Treatment for Participants With Human Epidermal Growth Factor 2 (HER2)-Positive and Programmed Death-ligand 1 (PD-L1)-Positive Locally Advanced (LABC) or Metastatic Breast Cancer (MBC)
NCT04768296	Berzosertib + Topotecan in Relapsed Platinum-Resistant Small-Cell Lung Cancer (DDRiver SCLC 250)
NCT04802070	Study of Adoptive Immunotherapy in Relapsed and Non-resectable Sarcomas After Multimodal Treatment.
NCT04838626	Study of Diagnostic Performance of [18F]CTT1057 for PSMA-positive Tumors Detection
NCT04854993	Recovery From dNMB Using Different Sugammadex Doses in Elderly Patients Undergoing Robot-assisted Prostatectomy
NCT05005273	A Study to Assess BMS-986207 in Combination With Nivolumab and Ipilimumab as First-line Treatment for Participants With Stage IV Non-Small Cell Lung Cancer
NCT05008224	Study of Safety and Efficacy of Pembrolizumab and Chemotherapy in Participants With Newly Diagnosed Classical Hodgkin Lymphoma (cHL) (MK-3475-C11/KEYNOTE-C11)
NCT05116202	A Study Evaluating the Efficacy and Safety of Multiple Treatment Combinations in Patients With Melanoma (Morpheus-Melanoma)
NCT05140382	AZD4573 as Monotherapy or in Combinations With Anti-cancer Agents in Patients With r/r PTCL or r/r cHL
NCT05144009	A Study of Loncastuximab Tesirine and Rituximab (Lonca-R) in Previously Untreated Unfit/Frail Participants With Diffuse Large B-cell Lymphoma (DLBCL)
NCT05155709	A Study of Siremadlin in Combination With Venetoclax Plus Azacitidine in Adult Participants With Acute Myeloid Leukemia (AML) Who Are Ineligible for Chemotherapy.
NCT05169684	A Study of BMS-986218 or BMS-986218 Plus Nivolumab in Combination With Docetaxel in Participants With Metastatic Castration-resistant Prostate Cancer
NCT05253807	Study to Evaluate the Efficacy and Safety of Pemigatinib in Participants With Relapsed or Refractory Advanced Non-Small Cell Lung Cancer With an FGFR Alteration
NCT05265988	Multiparametric Assessment of Bone Response in mCRPC Patients Treated With Cabozantinib
NCT05297565	A Study to Compare Nivolumab Administered Subcutaneously vs Intravenous in Melanoma Participants Following Complete Resection
NCT05630937	Study on Safety and Efficacy of NMS-01940153E in Adult Patients With Unresectable Hepatocellular Carcinoma (HCC) Previously Treated With Systemic Therapy
NCT05741294	A Study of Tirbanibulin on the Wellbeing of Participants With Actinic Keratoses
NCT05806853	PET/MR for Prostate Cancer Restaging: a Phase II Prospective Monocentric Study
NCT06098456	Epigallocatechin Gallate and Other Antural Compounds in HPV Infections
NCT04421963	Roll Over StudY for Patients Who Have Completed a Previous Oncology Study With Olaparib
NCT05303532	Roll Over StudY for Patients Who Have Completed a Previous Oncology Study With Durvalumab
NCT05604170	Open-label Study of Adjunctive GNX Treatment in Children and Adults With TSC-related Epilepsy
NCT02974556	Proactive Management of Endoperitoneal Spread in Colonic Cancer
NCT04250246	A Study of NIVO Plus IPI and Guadecitabine or NIVO Plus IPI in Melanoma and NSCLC Resistant to Anti-PD1/PDL1
NCT04709458	Safety and Early Efficacy Study of TBX-2400 in Patients With AML or Myelofibrosis
NCT04709731	Cardiovascular Assessment of Ponatinib as Third Line Treatment in Chronic Phase Chronic Myeloid Leukemia
NCT05703997	FASTing-like Approach and Maintenance IMMunotherapy in ES-SCLC Patients Not Progressing on Chemoimmunotherapy Induction
NCT05717660	APalutamiAPalutamide and stEReotactic Body Radiation Therapy for Metastatic Prostate Cancer
NCT06086288	Study of PembrolizumAb combiNeD With Cisplatin or carbOplatin and Etoposide in Treatment naÃ¯ve Advanced meRkel Cell cArcinoma (MCC)
NCT06115629	Surveillance After Resection of Oesophageal aNd Gastric Cancer (SARONG-II) Trial
NCT03448666	ECT-Pembrolizumab in Patients With Unresectable Melanoma With Superficial or Superficial and Visceral Metastases
NCT04031677	Surgery With or Without Neoadjuvant Chemotherapy in High Risk RetroPeritoneal Sarcoma
NCT04073706	Sentinel Node Biopsy in Endometrial Cancer
NCT04128072	Anti-CCR4 Monoclonal Antibody (Mogamulizumab) and Total Skin Electron Beam Therapy (TSEB) in Patients With Stage IB-IIB Cutaneous T-Cell Lymphoma
NCT04134598	ExclUsive endocRine Therapy Or Radiation theraPy for Women Aged â‰¥70 Years Early Stage Breast Cancer
NCT04241185	Efficacy and Safety of Pembrolizumab (MK-3475) in Combination With Chemoradiotherapy (CRT) Versus CRT Alone in Muscle-invasive Bladder Cancer (MIBC) (MK-3475-992/KEYNOTE-992)
NCT04250155	An Open-Label Dose-Escalation Study to Evaluate XmAb24306 as a Single Agent and in Combination With Atezolizumab in Participants With Locally Advanced or Metastatic Solid Tumors
NCT04277637	Study of Bcl-2 Inhibitor BGB-11417 in Participants With Mature B-Cell Malignancies
NCT04278768	Dose Escalation/ Expansion Study of CA-4948 as Monotherapy in Patients With Acute Myelogenous Leukemia (AML) or Myelodysplastic Syndrome (MDS)
NCT04279847	Safety and Tolerability Study of INCB057643 in Participants With Myelofibrosis and Other Advanced Myeloid Neoplasms
NCT04305054	Substudy 02B: Safety and Efficacy of Pembrolizumab in Combination With Investigational Agents or Pembrolizumab Alone in Participants With First Line (1L) Advanced Melanoma (MK-3475-02B/KEYMAKER-U02)
NCT04305548	Study on Trabectedin in Advanced Rearranged Mesenchymal Chondrosarcoma
NCT04342962	Tagraxofusp in Patients With CD123+ or With BPDCN-IPh-like Acute Myeloid Leukemia
NCT04373564	Effect on Body Movement and Mental Skills in Patients Who Received Gadolinium-based Contrast Media for Magnetic Resonance Examination Multiple Times Within 5 Years
NCT04379596	Ph1b/2 Study of the Safety and Efficacy of T-DXd Combinations in Advanced HER2-expressing Gastric Cancer (DESTINY-Gastric03)
NCT04383119	Trial in Patients With Metastatic or Locally Advanced Leiomyosarcoma
NCT04384484	Study to Evaluate Loncastuximab Tesirine With Rituximab Versus Immunochemotherapy in Participants With Relapsed or Refractory Diffuse Large B-Cell Lymphoma
NCT04442022	A Study of Rituximab-Gemcitabine-Dexamethasone-Platinum (R-GDP) With or Without Selinexor in Patients With Relapsed/Refractory Diffuse Large B-cell Lymphoma
NCT04442412	Prephase Treatment With Prednisone +/- Vitamin D Supplementation Followed by Immunochemotherapy
NCT04455841	INCB000928 Administered as a Monotherapy or in Combination With Ruxolitinib in Participants With Anemia Due to Myeloproliferative Disorders
NCT04463771	Safety and Efficacy of Retifanlimab (INCMGA00012) Alone or in Combination With Other Therapies in Participants With Advanced or Metastatic Endometrial Cancer Who Have Progressed on or After Platinum-based Chemotherapy.
NCT04464226	Study to Continue Treatment With Darolutamide in Patients Who Have Been Participating in Previous Darolutamide Studies Supported by Bayer
NCT04471987	Safety and Early Signs of Efficacy of IL12-L19L19.
NCT04475731	Ponatinib in Adult Ph+ ALL Patients With MRD Positivity or Hematological Relapse
NCT04480268	PAXG Out in the Country
NCT04482309	A Phase 2 Study of T-DXd in Patients With Selected HER2 Expressing Tumors
NCT04484623	Belantamab Mafodotin Plus Pomalidomide and Dexamethasone (Pd) Versus Bortezomib Plus Pd in Relapsed/Refractory Multiple Myeloma
NCT04509700	Rollover Study to Provide Continued Treatment for Participants With B-Cell Malignancies Previously Enrolled in Studies of Parsaclisib (INCB050465)
NCT04521231	A Study of Subcutaneous Blinatumomab Administration in Acute Lymphoblastic Leukemia (ALL) Patients
NCT04523688	Vaccination With Autologous Dendritic Cells Loaded With Autologous Tumour Homogenate in Glioblastoma
NCT04554914	A Study to Evaluate Tabelecleucel in Participants With Epstein-barr Virus-associated Diseases
NCT04557150	A Study Evaluating the Safety and Pharmacokinetics of Escalating Doses of Forimtamig in Participants With Relapsed or Refractory Multiple Myeloma (r/r MM)
NCT04561362	Study BT8009-100 in Subjects With Nectin-4 Expressing Advanced Malignancies
NCT04562389	Study of Selinexor in Combination With Ruxolitinib in Myelofibrosis
NCT04564898	Trifluridine/Tipiracil in Combination With Capecitabine and Bevacizumab in Metastatic Colorectal Cancer Patients.
NCT04573192	A Study to Evaluate Safety and Efficacy of L19TNF Plus Lomustine in Patients With Glioblastoma at First Progression
NCT04576156	A Study Comparing Imetelstat Versus Best Available Therapy for the Treatment of Intermediate-2 or High-risk Myelofibrosis (MF) Who Have Not Responded to Janus Kinase (JAK)-Inhibitor Treatment
NCT04585750	The Evaluation of PC14586 in Patients With Advanced Solid Tumors Harboring a TP53 Y220C Mutation (PYNNACLE)
NCT04589845	Tumor-Agnostic Precision Immuno-Oncology and Somatic Targeting Rational for You (TAPISTRY) Platform Study
NCT04603807	A Study to Compare the Efficacy and Safety of Entrectinib and Crizotinib in Participants With Advanced or Metastatic ROS1 Non-small Cell Lung Cancer (NSCLC) With and Without Central Nervous System (CNS) Metastases
NCT04612751	Phase 1b Study of Dato-DXd in Combination With Immunotherapy With or Without Carboplatin in Advanced or Metastatic Non-Small Cell Lung Cancer
NCT04613596	Phase 2 Trial of Adagrasib Monotherapy and in Combination With Pembrolizumab and a Phase 3 Trial of Adagrasib in Combination in Patients With a KRAS G12C Mutation KRYSTAL-7
NCT04617925	A Study of Belantamab Mafodotin in Patients With Relapsed or Refractory AL Amyloidosis
NCT04620239	ENdoluminal LIGHT ActivatED Treatment of Upper Tract Urothelial Cancer (ENLIGHTED) Study
NCT04623541	Safety and Efficacy Study of Epcoritamab in Subjects With Relapsed/Refractory Chronic Lymphocytic Leukemia and Richter's Syndrome
NCT04625907	FaR-RMS: An Overarching Study for Children and Adults With Frontline and Relapsed RhabdoMyoSarcoma
NCT04643002	Isatuximab in Combination With Novel Agents in RRMM - Master Protocol
NCT04644068	Study of AZD5305 as Monotherapy and in Combination With Anti-cancer Agents in Patients With Advanced Solid Malignancies
NCT04655118	Study of TL-895 in Subjects With Myelofibrosis or Indolent Systemic Mastocytosis
NCT04658862	A Study of TAR-200 in Combination With Cetrelimab Versus Concurrent Chemoradiotherapy in Participants With Muscle-invasive Bladder Cancer (MIBC) of the Bladder
NCT04660344	A Study of Atezolizumab Versus Placebo as Adjuvant Therapy in Patients With High-Risk Muscle-Invasive Bladder Cancer Who Are ctDNA Positive Following Cystectomy
NCT04663347	Safety and Efficacy Trial of Epcoritamab Combinations in Subjects With B-cell Non-Hodgkin Lymphoma (B-NHL)

NCT04681131	CAB-AXL-ADC Safety and Efficacy Study in Adults With NSCLC
NCT04681469	Induction and Maintenance Treatment With PARP Inhibitor and Immunotherapy in HPV-negative HNSCC
NCT04698213	Avelumab Plus Intermittent Axitinib in Previously Untreated Patients With Metastatic Renal Cell Carcinoma
NCT04699188	Study of JDQ443 in Patients With Advanced Solid Tumors Harboring the KRAS G12C Mutation
NCT04704934	Trastuzumab Deruxtecan for Subjects With HER2-Positive Gastric Cancer or Gastro-Esophageal Junction Adenocarcinoma After Progression on or After a Trastuzumab-Containing Regimen (DESTINY-Gastric04)
NCT04712097	A Study Evaluating the Efficacy and Safety of Mosunetuzumab in Combination With Lenalidomide in Comparison to Rituximab in Combination With Lenalidomide With a US Extension of Mosunetuzumab in Combination With Lenalidomide in Participants With Follicular Lymphoma
NCT04722848	Sequential Treatment With Ponatinib and Blinatumomab vs Chemotherapy and Imatinib in Newly Diagnosed Adult Ph+ ALL
NCT04728893	Efficacy and Safety of Nemtabrutinib (MK-1026) in Participants With Hematologic Malignancies (MK-1026-003)
NCT04733183	Efficacy and Safety of L19TNF in Previously Treated Patients With Advanced Stage or Metastatic Soft-tissue Sarcoma
NCT04758000	Metformin as Maintenance Therapy in Patients With Bone Sarcoma and High Risk of Relapse
NCT04773782	A Study of Avapritinib in Pediatric Patients With Solid Tumors Dependent on KIT or PDGFRA Signaling
NCT04777851	Regorafenib-pembrolizumab vs. TACE/TARE in Intermediate Stage HCC Beyond Up-to-7
NCT04787263	CD19-CAR_Lenti T Cells in Pediatric Patients Affected by Relapsed/Refractory CD19+ ALL and DLBCL or PML
NCT04787341	PAnitumumab REchallenge Followed by REgorafenib Versus the Reverse Sequence
NCT04794127	Study on Trabectedin in Combination With Pioglitazone in Patients Myxoid Liposarcomas With Stable Disease After T Alone.
NCT04796324	Anti-tumor Effect of Ixabepilone in Metastatic Breast Cancer (mBC) Selected by the Ixabepilone DRP.
NCT04797780	Tamibarotene Plus Azacitidine in Participants With Newly Diagnosed RARA-positive Higher-Risk Myelodysplastic Syndrome
NCT04803994	The ABC-HCC Trial: Atezolizumab Plus Bevacizumab vs. Transarterial Chemoembolization (TACE) in Intermediate-stage HepatoCellular Carcinoma
NCT04806646	Tailored Sonidegib Schedule After Complete Response in BCC
NCT04810078	A Study of Subcutaneous Nivolumab Versus Intravenous Nivolumab in Participants With Previously Treated Clear Cell Renal Cell Carcinoma That is Advanced or Has Spread
NCT04811001	Best EGFR-TKI Sequence in NSCLC Harboring EGFR Mutations
NCT04817826	TremelImumab aNd Durvalumab For the Non-operatIve Management (NOM) of MSI-high Resectable GC/GEJC.
NCT04819100	A Study of Selpercatinib After Surgery or Radiation in Participants With Non-Small Cell Lung Cancer (NSCLC)
NCT04830124	Nemvaleukin Alfa Monotherapy and in Combination With Pembrolizumab in Patients With Advanced Cutaneous or Mucosal Melanoma - ARTISTRY-6
NCT04832958	Imaging Guided Surgery to Improve the Detection of Lymph Node Metastases in Prostate Cancer Patients
NCT04835584	KRT-232 and TKI Study in Chronic Myeloid Leukemia
NCT04857372	A Phase I Study of IAG933 in Patients With Advanced Mesothelioma and Other Solid Tumors
NCT04862663	Capivasertib + CDK4/6i + Fulvestrant for Advanced/Metastatic HR+/HER2- Breast Cancer (CAPItello-292)
NCT04866654	Radiation Free Chemotherapy for Early Hodgkin Lymphoma
NCT04867928	Venetoclax and Azacitidine for the Management of Molecular Relapse/Progression in Adult NPM1-mutated Acute Myeloid Leukemia
NCT04877522	Asciminib Roll-over Study
NCT04884282	Efficacy of Tedopi Plus Docetaxel or Tedopi Plus Nivolumab as Second-line Therapy in Metastatic Non-small-cell Lung Cancer Progressing After First-line Chemo-immunotherapy (Combi-TED)
NCT04886804	A Study to Test Different Doses of Zongertinib in People With Different Types of Advanced Cancer (Solid Tumours With Changes in the HER2 Gene)
NCT04895436	Study to Assess Change in Disease Activity and Adverse Events of Oral Venetoclax With Intravenous (IV) Obinutuzumab in Adult Participants With Recurring Chronic Lymphocytic Leukemia (CLL)
NCT04895709	A Study of BMS-986340 as Monotherapy and in Combination With Nivolumab or Docetaxel in Participants With Advanced Solid Tumors
NCT04903197	Study of VAY736 as Single Agent and in Combination With Select Antineoplastic Agents in Patients With Non-Hodgkin Lymphoma
NCT04908228	Fixed-duration Therapy With Ibrutinib and Obinutuzumab (GA-101) in Treatment-naÃ¯ve Patients With CLL
NCT04910022	Ph I/II Study of NMS-03305293+TMZ in Adult Patients With Recurrent Glioblastoma
NCT04910685	(HARBOR) Study to Evaluate Efficacy and Safety of BLU-263 Versus Placebo in Patients With Indolent Systemic Mastocytosis
NCT04919226	Lutetium 177Lu-Edotreotide Versus Best Standard of Care in Well-differentiated Aggressive Grade-2 and Grade-3 GastroEnteroPancreatic NeuroEndocrine Tumors (GEP-NETs) - COMPOSE
NCT04919512	A Study of TAR-200 in Combination With Cetrelimab and Cetrelimab Alone in Participants With Muscle-Invasive Urothelial Carcinoma of the Bladder
NCT04919811	Taletrectinib Phase 2 Global Study in ROS1 Positive NSCLC
NCT04920149	Mesalamine for Colorectal Cancer Prevention Program in Lynch Syndrome
NCT04925479	Study to Determine the Dose and Safety of Asciminib in Pediatric Patients With Chronic Myeloid Leukemia
NCT04928846	A Study to Assess Disease Activity and Adverse Events of Intravenous (IV) Telisotuzumab Vedotin Compared to IV Docetaxel in Adult Participants With Previously Treated Non-Squamous Non-Small Cell Lung Cancer (NSCLC)
NCT04940637	UNITO-001- Study in HRR/PDL1 Positive MPM/NSCLC
NCT04949256	Efficacy and Safety of Pembrolizumab (MK-3475) Plus Lenvatinib (E7080/MK-7902) Plus Chemotherapy in Participants With Metastatic Esophageal Carcinoma (MK-7902-014/E7080-G000-320/LEAP-014)
NCT04950075	Study of INBRX-109 in Conventional Chondrosarcoma
NCT04951622	A Study of Nipocalimab Administered to Adults With Generalized Myasthenia Gravis
NCT04958239	A Study to Test BI 765179 Alone and in Combination With Ezabenlimab in Patients With Advanced Cancer (Solid Tumors) and BI 765179 in Combination With Pembrolizumab in Patients With Advanced Head and Neck Cancer
NCT04964934	Phase III Study to Assess AZD9833+ CDK4/6 Inhibitor in HR+/HER2-MBC With Detectable ESR1m Before Progression (SERENA-6)
NCT04965493	A Trial of Pirtobrutinib (LOXO-305) Plus Venetoclax and Rituximab (PVR) Versus Venetoclax and Rituximab (VR) in Previously Treated Chronic Lymphocytic Leukemia/Small Lymphocytic Lymphoma (CLL/SLL)
NCT04970901	A Study to Evaluate the Safety and Anti-cancer Activity of Loncastuximab Tesirine in Combination With Other Anti-cancer Agents in Participants With Relapsed or Refractory B-cell Non-Hodgkin Lymphoma (LOTIS-7)
NCT04973605	A Phase 1b/2 Study of BGB-11417in Monotherapy and in Various Combinations With Dexamethasone and Carfilzomib in Multiple Myeloma
NCT04988555	A Study of DSP-5336 in Relapsed/Refractory AML/ ALL With or Without MLL Rearrangement or NPM1 Mutation
NCT04994717	Study Comparing Blinatumomab Alternating With Low-intensity Chemotherapy Versus Standard of Care Chemotherapy for Older Adults With Newly Diagnosed Philadelphia-negative B-cell Precursor Acute Lymphoblastic Leukemia
NCT04996875	(Apex) Bezuclastinib in Patients With Advanced Systemic Mastocytosis
NCT05002127	A Study of Evorpacept (ALX148) in Patients With Advanced HER2+ Gastric Cancer (ASPEN-06)
NCT05006716	A Dose-Escalation and Expansion Study of BGB-16673 in Participants With B-Cell Malignancies
NCT05019846	SRT Versus SRT+ADT in Prostate Cancer
NCT05020236	MagnetisMM-5: Study of Elranatamab (PF-06863135) Monotherapy and Elranatamab + Daratumumab Versus Daratumumab + Pomalidomide + Dexamethasone in Participants With Relapsed/Refractory Multiple Myeloma
NCT05023967	Metformin and Nightly Fasting in Women With Early Breast Cancer
NCT05024773	Study of ONCOFID-P-B (PACLITAXEL-HYALURONIC ACID)
NCT05027594	Ph I Study in Adult Patients With Relapsed or Refractory Multiple Myeloma
NCT05051735	PARASTOP - Paracetamol With Strong Opioids
NCT05058404	Shortened vs Standard Chemotherapy Combined With Immunotherapy for the Initial Treatment of Patients With High Tumor Burden Follicular Lymphoma
NCT05061550	Neoadjuvant and Adjuvant Treatment in Resectable Non-small Cell Lung Cancer
NCT05062889	Exploiting Circulating Tumour DNA to Intensify the Postoperative Treatment Resected Colon Cancer Patients
NCT05067283	A Study of MK-1084 in KRAS Mutant Advanced Solid Tumors (MK-1084-001)
NCT05070858	A Study to Test How Safe Pozelimab and Cemdisiran Combination Therapy and Cemdisiran Alone Are and How Well They Work in Adult Patients With Generalized Myasthenia Gravis
NCT05081609	A Study to Investigate Safety and Tolerability of TransCon IL-2 Î²/Î³ Alone or in Combination With Pembrolizumab and/or Chemotherapy or TransCon TLR7/8 Agonist in Adult Participants With Locally Advanced or Metastatic Solid Tumor Malignancies
NCT05099172	First in Human Study of BAY2927088 in Participants Who Have Advanced Non-small Cell Lung Cancer (NSCLC) With Mutations in the Genes of Epidermal Growth Factor Receptor (EGFR) and/or Human Epidermal Growth Factor Receptor 2 (HER2)
NCT05100862	A Study of Zanubrutinib Plus Anti-CD20 Versus Lenalidomide Plus Rituximab in Participants With Relapsed/Refractory Follicular or Marginal Zone Lymphoma
NCT05111626	Bemarituzumab Plus Chemotherapy and Nivolumab Versus Chemotherapy and Nivolumab for FGFR2b Overexpressed Untreated Advanced Gastric and Gastroesophageal Junction Cancer.
NCT05118789	A Study of NVL-520 in Patients With Advanced NSCLC and Other Solid Tumors Harboring ROS1 Rearrangement (ARROS-1)
NCT05120349	A Global Study to Assess the Effects of Osimertinib in Participants With EGFRm Stage IA2-IA3 NSCLC Following Complete Tumour Resection
NCT05139017	A Study of Zilovertamab Vedotin (MK-2140) in Combination With Standard of Care in Participants With Relapsed or Refractory Diffuse Large B-Cell Lymphoma (rrDLBCL) (MK-2140-003)
NCT05152147	A Study of Zanidatamab in Combination With Chemotherapy Plus or Minus Tislelizumab in Patients With HER2-positive Advanced or Metastatic Gastric and Esophageal Cancers
NCT05153239	Clinical Trial of Lurbinectedin as Single-agent or in Combination With Irinotecan Versus Topotecan or Irinotecan in Patients With Relapsed Small-cell Lung Cancer (LAGOON)

NCT05161195	Roll-over Study to Allow Continued Access to Ribociclib
NCT05171049	A Study Comparing Abelacimab to Apixaban in the Treatment of Cancer-associated VTE
NCT05171075	A Study Comparing Abelacimab to Dalteparin in the Treatment of Gastrointestinal/Genitourinary Cancer and Associated VTE
NCT05176483	Study of XL092 in Combination With Immuno-Oncology Agents in Subjects With Solid Tumors
NCT05180474	GEN1047 for Solid Tumors - First in Human (FIH) Trial
NCT05183035	Venetoclax in Children With Relapsed Acute Myeloid Leukemia (AML)
NCT05186753	(Summit) A Study to Evaluate the Efficacy and Safety of CGT9486 Versus Placebo in Patients With Indolent or Smoldering Systemic Mastocytosis
NCT05194072	A Study of SGN-B7H4V in Advanced Solid Tumors
NCT05201066	Roll-over Study for Patients Who Have Completed a Prior Novartis-sponsored Sabatolimab (MBG453) Study and Are Judged by the Investigator to Benefit From Continued Treatment With Sabatolimab.
NCT05201547	Endometrial Cancer Patientes MMR Deficient Comparing Chemotherapy vs Dostarlimab in First Line
NCT05203172	The FLOTILLA Study: Providing Continued Access to The Study Medicines Encorafenib and Binimetinib for Participants in Prior Clinical Trials
NCT05206357	Study of the Adverse Events and Change in Disease State of Pediatric Participants (and Young Adults Between the Ages of 18-25) With Relapsed/Refractory Aggressive Mature B-cell Neoplasms Receiving Subcutaneous (SC) Injections of Epcoritamab
NCT05208047	(Peak) A Phase 3 Randomized Trial of CGT9486+Sunitinib vs. Sunitinib in Subjects With Gastrointestinal Stromal Tumors
NCT05208762	A Study of PF-08046054/SGN-PDL1V in Advanced Solid Tumors
NCT05211895	A Global Study to Assess the Effects of Durvalumab + Domvanalimab Following Concurrent Chemoradiation in Participants With Stage III Unresectable NSCLC
NCT05215340	Study of Dato-DXd Plus Pembrolizumab vs Pembrolizumab Alone in the First-line Treatment of Subjects With Advanced or Metastatic NSCLC Without Actionable Genomic Alterations
NCT05217446	A Study of Encorafenib Plus Cetuximab Taken Together With Pembrolizumab Compared to Pembrolizumab Alone in People With Previously Untreated Metastatic Colorectal Cancer
NCT05221840	A Global Study to Assess the Effects of Durvalumab With Oleclumab or Durvalumab With Monalizumab Following Concurrent Chemoradiation in Patients With Stage III Unresectable Non-Small Cell Lung Cancer
NCT05229614	Immunotherapy and Carbon Ion Radiotherapy In Solid Cancers With Stable Disease
NCT05232916	Phase 3 Study to Evaluate the Efficacy and Safety of HER2/Neu Peptide GLSI-100 (GP2 + GM-CSF) in HER2/Neu Positive Subjects
NCT05238922	Study of INCB123667 in Subjects With Advanced Solid Tumors
NCT05243797	Phase 3 Study of Teclistamab in Combination With Lenalidomide and Teclistamab Alone Versus Lenalidomide Alone in Participants With Newly Diagnosed Multiple Myeloma as Maintenance Therapy Following Autologous Stem Cell Transplantation
NCT05249959	Consolidation With Loncastuximab Tesirine After a Short Course of Immunochemotherapy in BTKi-treated (or Intolerant) Relapsed/Refractory Mantle Cell Lymphoma Patients.
NCT05252403	Residual Disease Driven Strategy for CARCIK (CD19) in Adults/Pediatric BCP-ALL
NCT05252416	(VELA) Study of BLU-222 in Advanced Solid Tumors
NCT05253651	A Study of Tucatinib With Trastuzumab and mFOLFOX6 Versus Standard of Care Treatment in First-line HER2+ Metastatic Colorectal Cancer
NCT05254171	Study of Nab-Paclitaxel and Gemcitabine With or Without SBP-101 in Pancreatic Cancer
NCT05255471	MITO 35B: Olaparib Beyond Progression Compared to Platinum Chemotherapy After Secondary Cytoreductive Surgery in Recurrent Ovarian Cancer Patients.
NCT05259839	A Study to Assess Adverse Events and Change in Disease Activity of Intravenously (IV) Infused ABBV-383 in Combination With Anti-Cancer Regimens for the Treatment of Adult Participants With Relapsed/Refractory Multiple Myeloma
NCT05261399	Savolitinib Plus Osimertinib Versus Platinum-based Doublet Chemotherapy in Participants With Non-Small Cell Lung Cancer Who Have Progressed on Osimertinib Treatment
NCT05291156	CAVE-2 GOIM Study: a Clinical Study of the Combination of Avelumab Plus Cetuximab as Rechallenge Strategy
NCT05298423	Study of Pembrolizumab/Vibostolimab (MK-7684A) in Combination With Concurrent Chemoradiotherapy Followed by Pembrolizumab/Vibostolimab Versus Concurrent Chemoradiotherapy Followed by Durvalumab in Participants With Stage III Non-small Cell Lung Cancer (MK-7684A-006/KEYVIBE-006)
NCT05298995	GD2-CAR T Cells for Pediatric Brain Tumours
NCT05299437	Metastatic Thyroid Cancer Therapy Optimization With 124I PET Dosimetry
NCT05300282	Study of Atezolizumab Plus BEGEV Regimen in Relapsed or Refractory Hodgkin's Lymphoma Patients
NCT05301842	Evaluate Durvalumab and Tremelimumab +/- Lenvatinib in Combination With TACE in Patients With Locoregional HCC
NCT05303714	PIPAC in Multimodal Therapy for Patients With Oligometastatic Peritoneal Gastric Cancer
NCT05306301	Ponatinib Plus Chemotherapy in Acute Lymphoblastic Leukemia Patients
NCT05307705	A Study of LOXO-783 in Patients With Breast Cancer/Other Solid Tumors
NCT05316922	TSH Suppression During Radiotherapy on Thyroid Site to Prevent Iatrogenic Hypothyroidism in Pediatric Cancer Patients
NCT05317416	Study With Elranatamab Versus Lenalidomide in Patients With Newly Diagnosed Multiple Myeloma After Transplant
NCT05319587	Study of Liposomal Annamycin in Combination With Cytarabine for the Treatment of Subjects With Acute Myeloid Leukemia (AML)
NCT05319730	A Study to Evaluate Investigational Agents With or Without Pembrolizumab (MK-3475) in Participants With Advanced Esophageal Cancer Previously Exposed to Programmed Cell Death 1 Protein (PD-1)/ Programmed Cell Death Ligand 1 (PD-L1) Treatment (MK-3475-06B)
NCT05325866	A Study Evaluating Bemarituzumab in Solid Tumors With Fibroblast Growth Factor Receptor 2b (FGFR2b) Overexpression
NCT05327894	Interfant-21 Treatment Protocol for Infants Under 1 Year With KMT2A-rearranged ALL or Mixed Phenotype Acute Leukemia
NCT05329792	L19IL2/L19TNF in Skin Cancer Patients
NCT05337137	A Study of Nivolumab and Relatlimab in Combination With Bevacizumab in Advanced Liver Cancer
NCT05352672	Clinical Study of Fianlimab in Combination With Cemiplimab Versus Pembrolizumab in Adolescent and Adult Patients With Previously Untreated Unresectable Locally Advanced or Metastatic Melanoma
NCT05360264	tailOred dRug repurposIng of dEcitabine in KRAS-dependeNt refracTory pAncreaTic cancEr
NCT05367440	Study of AZD5305 When Given in Combination With New Hormonal Agents in Patients With Metastatic Prostate Cancer
NCT05371093	Study of Axicabtagene Ciloleucel Versus Standard of Care Therapy in Participants With Relapsed/Refractory Follicular Lymphoma
NCT05371223	Combined Nabpaclitaxel Pressurized IntraPeritoneal Aerosol Chemotherapy With Systemic Nabpaclitaxel-Gemcitabine Chemotherapy for Pancreatic Cancer Peritoneal Metastases
NCT05375903	A Phase 1 Dose-escalation Study of UGN-301 in Patients With Recurrent Non-muscle Invasive Bladder Cancer (NMIBC)
NCT05379595	A Study of Amivantamab Monotherapy and in Addition to Standard-of-Care Chemotherapy in Participants With Advanced or Metastatic Colorectal Cancer
NCT05382299	Study of Sacituzumab Govitecan-hziy Versus Treatment of Physician's Choice in Patients With Previously Untreated Locally Advanced Inoperable or Metastatic Triple-Negative Breast Cancer
NCT05382741	Adjuvant Durvalumab Plus Regorafenib vs Untreated Control in Stage IV Colorectal Cancer Patients With no Evidence of Disease (NED): VIVA Trial
NCT05384626	A Study of NVL-655 in Patients With Advanced NSCLC and Other Solid Tumors Harboring ALK Rearrangement or Activating ALK Mutation (ALKOVE-1)
NCT05386108	Study of Abemaciclib and Elacestrant in Patients With Brain Metastasis Due to ER+/HER-2- Breast Cancer
NCT05387525	A Study of Tirbanibulin Ointment and Diclofenac Sodium Gel for the Treatment of Adult Participants With Actinic Keratosis on the Face or Scalp
NCT05395962	Electrochemotherapy With Carboplatinum Plus Bleomycin Versus Bleomycin Alone in Vulvar Cancer
NCT05397496	Study of PIT565 in Relapsed and/or Refractory B-cell Malignancies
NCT05403385	Study of Inupadenant (EOS100850) With Chemotherapy as Second Line Treatment for Nonsquamous Non-small Cell Lung Cancer
NCT05403554	A Study of NI-1801 in Patients With Mesothelin Expressing Solid Cancers
NCT05409066	Study of Subcutaneous Epcoritamab in Combination With Intravenous Rituximab and Oral Lenalidomide (R2) to Assess Adverse Events and Change in Disease Activity in Adult Participants With Follicular Lymphoma
NCT05416307	Evaluation of the Safety and Efficacy of ELA026 in Participants With Secondary Hemophagocytic Lymphohistiocytosis
NCT05419375	Screening Study for Participants With Malignant Tumors
NCT05429502	Study of Efficacy and Safety of Ribociclib (LEE011) in Combination With Topotecan and Temozolomide (TOTEM) in Pediatric Patients With Relapsed or Refractory Neuroblastoma and Other Solid Tumors
NCT05438043	A Study of Daratumumab
NCT05440786	CAMPFIRE: A Study of Abemaciclib (LY2835219) in Participants With Ewing's Sarcoma
NCT05440864	Durvalumab and Tremelimumab in Resectable HCC
NCT05453903	A Study of Bleximenib in Combination With Acute Myeloid Leukemia (AML) Directed Therapies
NCT05455320	A Study Comparing Talquetamab in Combination With Daratumumab or in Combination With Daratumumab and Pomalidomide Versus Daratumumab in Combination With Pomalidomide and Dexamethasone in Participants With Multiple Myeloma That Returns After Treatment or is Resistant to Treatment
NCT05456191	A Study to Investigate Tolerability and Efficacy of Asciminib (Oral) Versus Nilotinib (Oral) in Adult Participants (â‰¥18 Years of Age) With Newly Diagnosed Philadelphia Chromosome Positive Chronic Myelogenous Leukemia in Chronic Phase (Ph+ CML-CP)
NCT05458297	A Study of Zilovertamab Vedotin (MK-2140) as Monotherapy and in Combination in Participants With Aggressive and Indolent B-cell Malignancies (MK-2140-006)
NCT05462873	A Study to Investigate the Safety and Tolerability of Intravenous QEQ278 in Patients With Advanced Solid Tumors
NCT05466799	FOLFIRINOX Versus OncoSilâ„¢ in Addition to FOLFIRINOX in Patients With Locally Advanced Pancreatic Adenocarcinoma
NCT05475925	A Study of DR-01 in Subjects With Large Granular Lymphocytic Leukemia or Cytotoxic Lymphomas
NCT05478512	Front-line VenObi Combination Followed by Ven or VenZan Combination in Patients With Residual Disease: a MRD Tailored Treatment for Young Patients With High-risk CLL
NCT05480865	SHP2 Inhibitor BBP-398 in Combination With Sotorasib in Patients With Advanced Solid Tumors and a KRAS-G12C Mutation
NCT05489211	Study of Dato-Dxd as Monotherapy and in Combination With Anti-cancer Agents in Patients With Advanced Solid Tumours (TROPION-PanTumor03)

NCT05498428	A Study of Amivantamab in Participants With Advanced or Metastatic Solid Tumors Including Epidermal Growth Factor Receptor (EGFR)-Mutated Non-Small Cell Lung Cancer
NCT05501886	Gedatolisib Plus Fulvestrant With or Without Palbociclib vs Standard-of-Care for the Treatment of Patients With Advanced or Metastatic HR+/HER2- Breast Cancer (VIKTORIA-1)
NCT05503797	A Study to Assess the Efficacy and Safety of FORE8394 in Participants With Cancer Harboring BRAF Alterations
NCT05512364	Elacestrant for Treating ER+/HER2- Breast Cancer Patients With ctDNA Relapse (TREAT ctDNA)
NCT05514054	A Study of Imlunestrant Versus Standard Endocrine Therapy in Participants With Early Breast Cancer
NCT05522660	Immunotherapy or Targeted Therapy With or Without Stereotactic Radiosurgery for Patients With Brain Metastases From Melanoma or Non-small Cell Lung Cancer
NCT05526755	A Study of 5 Years of Adjuvant Osimertinib in Completely Resected Epidermal Growth Factor Receptor Mutation (EGFRm) Non-small Cell Lung Carcinoma (NSCLC)
NCT05533775	A Study to Evaluate Glofitamab Monotherapy and Glofitamab + Chemoimmunotherapy in Pediatric and Young Adult Participants With Relapsed/Refractory Mature B-Cell Non-Hodgkin Lymphoma
NCT05535244	A Study Evaluating the Efficacy and Safety of Cevostamab in Prior B Cell Maturation Antigen (BCMA)-Exposed Participants With Relapsed/Refractory Multiple Myeloma
NCT05544929	A Study of Safety and Efficacy of KFA115 Alone and in Combination With Pembrolizumab in Patients With Select Advanced Cancers
NCT05548127	TACTIVE-U: A Study to Learn About the Study Medicine (Vepdegestrant) When Given With Other Medicines in People With Advanced or Metastatic Breast Cancer (Sub-Study A)
NCT05549297	Tebentafusp Regimen Versus Investigator's Choice in Previously Treated Advanced Melanoma (TEBE-AM)
NCT05552222	A Study of Teclistamab in Combination With Daratumumab and Lenalidomide (Tec-DR) and Talquetamab in Combination With Daratumumab and Lenalidomide (Tal-DR) in Participants With Newly Diagnosed Multiple Myeloma
NCT05552976	A Study to Evaluate Mezigdomide in Combination With Carfilzomib and Dexamethasone (MeziKD) Versus Carfilzomib and Dexamethasone (Kd) in Participants With Relapsed or Refractory Multiple Myeloma (SUCCESSOR-2)
NCT05554003	Metronomic Temozolomide in Unfit NENs Patients Metronomic Temozolomide in Unfit Patients With Advanced Neuroendocrine Neoplasms (NENs): MeTe Study
NCT05555732	Datopotamab Deruxtecan (Dato-DXd) and Pembrolizumab With or Without Platinum Chemotherapy in 1L Non-Small Cell Lung Cancer (TROPION-Lung07)
NCT05556096	Safety and Efficacy of ALXN1720 in Adults With Generalized Myasthenia Gravis
NCT05557604	SbrtÃ«sTad for Unfavorable iNtermediate rIsk/High Risk Prostate caNcer
NCT05566795	DAY101 vs. Standard of Care Chemotherapy in Pediatric Patients With Low-Grade Glioma Requiring First-Line Systemic Therapy (LOGGIC/FIREFLY-2)
NCT05568017	Neoadjuvant PRRT With Y-90-DOTATOC in pNET
NCT05568212	Randomized Trial Comparing Standard of Care Versus Immune- Based Combination in Relapsed Stage III Non-small-cell Lung Cancer (NSCLC) Pretreated With Chemoradiotherapy and Durvalumab
NCT05573555	TACTIVE-U: A Study to Learn About the Study Medicine (Vepdegestrant) When Given With Other Medicines in People With Advanced or Metastatic Breast Cancer (Sub-Study B)
NCT05577182	Study of INCA32459 a LAG-3 and PD-1 Bispecific Antibody in Participants With Select Advanced Malignancies
NCT05578664	Efficacy of PErioperative PEmbrolizumab Treatment in Patients With Resectable Metastases From Kidney Cancer
NCT05578872	A Study of ANV419 Alone or in Combination With Approved Treatment in Patients With Cutaneous Melanoma (OMNIA-1).
NCT05580562	ONC201 in H3 K27M-mutant Diffuse Glioma Following Radiotherapy (the ACTION Study)
NCT05581121	PARa-aOrtic LymphAdenectomy in Locally Advanced Cervical Cancer
NCT05582538	Restoring Sensitivity To Immunotherapy In Advanced Triple Negative Breast Cancer Exploiting Ceralasertib Priming Followed By Combined Durvalumab/Nab-Paclitaxel
NCT05586516	A Study to Assess an ATX Inhibitor (IOA-289) in Patients With Metastatic Pancreatic Cancer
NCT05594290	Chemo-immunotherapy in Patients With Resectable Merkel Cell Carcinoma Prior to Surgery
NCT05601700	Letrozole for Estrogen/Progesterone Receptor Positive Low-grade Serous Epithelial Ovarian Cancer (LEPRE Trial)
NCT05605899	Study to Compare Axicabtagene Ciloleucel With Standard of Care Therapy as First-line Treatment in Participants With High-risk Large B-cell Lymphoma
NCT05607550	Study to Compare Furmonertinib to Platinum-Based Chemotherapy for Patients With Locally Advanced or Metastatic Non-Small Cell Lung Cancer (NSCLC) With Epidermal Growth Factor Receptor (EGFR) Exon 20 Insertion Mutations (FURVENT)
NCT05608291	A Trial to See if the Combination of Fianlimab With Cemiplimab Works Better Than Pembrolizumab for Preventing or Delaying Melanoma From Coming Back After it Has Been Removed With Surgery
NCT05609578	Combination Therapies With Adagrasib in Patients With Advanced NSCLC With KRAS G12C Mutation
NCT05609968	Study of Pembrolizumab (MK-3475) Monotherapy Versus Sacituzumab Govitecan in Combination With Pembrolizumab for Participants With Metastatic Non-small Cell Lung Cancer (NSCLC) With Programmed Cell Death Ligand 1 (PD-L1) Tumor Proportion Score (TPS) â‰¥50% (MK-3475-D46)
NCT05614739	A Study of LOXO-435 in Participants With Cancer With a Change in a Gene Called FGFR3
NCT05623319	Pembrolizumab and Olaparib Treatment of Extensive Small Cell Lung Cancer (ES-SCLC)
NCT05625399	A Study of Subcutaneous Nivolumab + Relatlimab Fixed-dose Combination (FDC) in Previously Untreated Metastatic or Unresectable Melanoma
NCT05625412	A Study of BMS-986360/CC-90001 Alone and in Combination With Chemotherapy or Nivolumab in Advanced Solid Tumors
NCT05629585	A Study of Dato-DXd With or Without Durvalumab Versus Investigator's Choice of Therapy in Patients With Stage I-III Triple-negative Breast Cancer Without Pathological Complete Response Following Neoadjuvant Therapy (TROPION-Breast03)
NCT05634499	A Study of Giredestrant in Participants With Grade 1 Endometrial Cancer
NCT05635708	A Study of Tislelizumab in Combination With Investigational Agents in Participants With Non-Small Cell Lung Cancer
NCT05647122	First in Human Study of AZD9592 in Solid Tumors
NCT05650879	ELVN-002 in HER2 Mutant Non-Small Cell Lung Cancer
NCT05654623	A Study to Learn About a New Medicine Called ARV-471 (PF-07850327) in People Who Have Advanced Metastatic Breast Cancer.
NCT05660967	Subcutaneous Epcoritamab With or Without Lenalidomide as First Line Therapy for Diffuse Large B-Cell Lymphoma
NCT05665530	A Study of PRT2527 as Monotherapy and in Combination With Zanubrutinib or Venetoclax in Participants With R/R Hematologic Malignancies
NCT05667636	Early Salvage Stereotactic Radiotherapy for Biochemical Failure After RP
NCT05668988	A Study of DZD9008 Versus Platinum-Based Doublet Chemotherapy in Local Advanced or Metastatic Non-small Cell Lung Cancer (WU-KONG28)
NCT05669989	International Treatment-extension Study in Adult Participants With Multiple Myeloma and Who Have Derived Clinical Benefit From Isatuximab
NCT05671510	ONC-392 Versus Docetaxel in Metastatic NSCLC That Progressed on PD-1/PD-L1 Inhibitors
NCT05673187	Adagrasib in Patients With KRASG12C-mutant NSCLC Who Are Elderly or Have Poor Performance Status
NCT05678673	Study of XL092 + Nivolumab vs Sunitinib in Subjects With Advanced or Metastatic Non-Clear Cell Renal Cell Carcinoma
NCT05694312	Ibrutinib for the Treatment of AIHA in Patients With CLL/SLL or CLL-like MBL
NCT05696626	Evaluation of Lasofoxifene Combined With Abemaciclib Compared With Fulvestrant Combined With Abemaciclib in Locally Advanced or Metastatic ER+/HER2- Breast Cancer With an ESR1 Mutation
NCT05714202	A Study of TAR-200 in Combination With Cetrelimab or TAR-200 Alone Versus Intravesical Bacillus Calmette-GuÃ©rin (BCG) in Participants With BCG-naÃ¯ve High-risk Non-muscle Invasive Bladder Cancer (HR-NMIBC)
NCT05723562	A Study of Dostarlimab in Untreated dMMR/MSI-H Locally Advanced Rectal Cancer
NCT05727176	Study of Futibatinib in Patients With Advanced Cholangiocarcinoma With FGFR2 Fusion or Rearrangement
NCT05727904	Study to Investigate Lifileucel Regimen Plus Pembrolizumab Compared With Pembrolizumab Alone in Participants With Untreated Advanced Melanoma.
NCT05733572	Safety and Efficacy of the PAINLESS Nerve Growth Factor CHF6467 in Optic Pathway Glioma (OPG)
NCT05734105	A Study of Ripretinib vs Sunitinib in Patients With Advanced GIST With Specific KIT Exon Mutations Who Were Previously Treated With Imatinib
NCT05743036	ZN-c3 in Adult Participants With Metastatic Colorectal Cancer
NCT05748171	A Study to Learn More About the Study Medicine Called Inotuzumab Ozogamicin (InO) in Children (1 to <18 Years) With First Relapse ALL
NCT05756153	A Study of GFH925 in Combination With Cetuximab in Previously Untreated Advanced NSCLC Harboring KRAS G12C Mutation
NCT05763992	Targeting Triple Negative BREAst Cancer Metabolism With a Combination of Chemoimmunotherapy and a FASTing-like Approach in the Preoperative Setting: the BREAKFAST 2 Trial
NCT05765734	A Study of TAS3351 in NSCLC Patients With EGFRmt
NCT05768139	First-in-Human Study of STX-478 as Monotherapy and in Combination With Other Antineoplastic Agents in Participants With Advanced Solid Tumors
NCT05771480	Durvalumab With Chemotherapy as First Line Treatment in Patients With Advanced Biliary Tract Cancers (aBTCs)
NCT05772390	Partial Breast Re-irradiation in Women in Women With Locally Recurrent Breast Cancer Previously Treated With Conservative Surgery and Whole Breast Irradiation
NCT05774951	A Study of Camizestrant in ER+/HER2- Early Breast Cancer After at Least 2 Years of Standard Adjuvant Endocrine Therapy
NCT05775159	Study of Novel Immunomodulators as Monotherapy and in Combination With Anticancer Agents in Participants With Advanced Hepatobiliary Cancer
NCT05784597	A Study to Evaluate the Safety and Dosimetry of 68Ga-labelled OncoFAP Derivatives in Solid Tumors
NCT05785208	Efficacy Study of Osimertinib in Treatment-naÃ¯ve Patients With EGFR Mutant NSCLC According to TP53 Mutational Status.
NCT05789069	A Study of HFB200603 as a Single Agent and in Combination With Tislelizumab in Adult Patients With Advanced Solid Tumors
NCT05794906	A Study to Compare Darolutamide Given With Androgen Deprivation Therapy (ADT) With ADT in Men With Hormone Sensitive Prostate Cancer and Raise of Prostate Specific Antigen (PSA) Levels After Local Therapies
NCT05797831	Study of Navtemadlin as Maintenance Therapy in TP53WT Advanced or Recurrent Endometrial Cancer

NCT05817903	Axitinib Intensification Plus Nivolumab or Nivolumab Alone After Nivolumab Plus Ipilimumab in mRCC Patients
NCT05821556	Valproic Acid/Simvastatin Plus Gemcitabine/Nab-paclitaxel Based Regimens in Untreated Metastatic Pancreatic Adenocarcinoma Patients
NCT05836324	A Study to Evaluate the Safety of INCA33890 in Participants With Advanced or Metastatic Solid Tumors
NCT05838768	Study of HRO761 Alone or in Combination in Cancer Patients With Specific DNA Alterations Called Microsatellite Instability or Mismatch Repair Deficiency.
NCT05839626	A Study to Investigate Safety and Efficacy With SAR445514 in Participants With Relapsed/Refractory Multiple Myeloma (RRMM) and Relapsed/Refractory Light-chain Amyloidosis (RRLCA)
NCT05845450	Pre-operative Targeted Treatments in Molecularly Selected Resectable Colorectal Cancer (UNICORN)
NCT05849298	A Phase II Study of AAA617 Alone and AAA617 in Combination With ARPI in Patients With PSMA PET Scan Positive CRPC
NCT05853575	Trial of Two Adagrasib Dosing Regimens in NSCLC With KRAS G12C Mutation (KRYSTAL 21)
NCT05855200	Study of Perioperative Dostarlimab in Participants With Untreated T4N0 or Stage III dMMR/MSI-H Resectable Colon Cancer
NCT05861336	GEM+Nab-Paclitaxel Plus Losartan Followed by Stereotactic Radiotherapy for Locally Advanced Pancreatic Cancer
NCT05867615	Radiometabolic Therapy With 177Lu PSMA in PSMA PET/CT Positive Advanced/Metastatic Tumours:
NCT05868837	Rituximab EfFicacy IN MyasthEnia Gravis (REFINE)
NCT05876754	An Early Access Study of Ivosidenib in Patients With a Pretreated Locally Advanced or Metastatic Cholangiocarcinoma
NCT05882734	Tuvusertib (M1774) in Combination With Cemiplimab in Participants With Non-Squamous NSCLC (DDRiver NSCLC 322)
NCT05883644	Durvalumab and Tremelimumab as First Line Treatment in Participants With Advanced Hepatocellular Carcinoma (HCC)
NCT05888831	A Study of BMS-986449 With and Without Nivolumab in Participants With Advanced Solid Tumors
NCT05893381	Lu-PSMA and Stereotactic Radiotherapy Versus Radiotherapy Alone for Prostate Cancer (LUST)
NCT05894239	A Study to Evaluate the Efficacy and Safety of Inavolisib in Combination With Phesgo Versus Placebo in Combination With Phesgo in Participants With PIK3CA-Mutated HER2-Positive Locally Advanced or Metastatic Breast Cancer
NCT05896566	A Window-of-Opportunity Trial of Giredestrant +/- Triptorelin vs. Anastrozole + Triptorelin in Premenopausal Patients With ER-positive/HER2-negative Early Breast Cancer
NCT05902169	Sonocloud-9 in Association With Carboplatin Versus Standard-of-Care Chemotherapies (CCNU or TMZ) in Recurrent GBM
NCT05907057	An Open-label Phase 3b Study of Ivosidenib in Combination With Azacitidine in Adult Patients Newly Diagnosed With IDH1m Acute Myeloid Leukemia (AML) Ineligible for Intensive Induction Chemotherapy.
NCT05907122	A Study to Evaluate Similarity of ABP 206 Compared With OPDIVOÂ® (Nivolumab) in Subjects With Resected Melanoma
NCT05907954	(Neo)Adjuvant IDE196 (Darovasertib) in Patients With Localized Ocular Melanoma
NCT05908734	A Study of Combination Therapy With Amivantamab and Cetrelimab in Participants With Metastatic Non-small Cell Lung Cancer
NCT05913427	Evaluation of the Efficacy of Addition of Progesterone to Standard Chemotherapy in Adrenocortical Carcinoma (ACC)
NCT05918302	Efficacy and Safety of Radiotherapy Compared to Everolimus in Somatostatin Receptor Positive Neuroendocrine Tumors of the Lung and Thymus.
NCT05920356	A Study Evaluating Sotorasib Platinum Doublet Combination Versus Pembrolizumab Platinum Doublet Combination as a Front-Line Therapy in Participants With Stage IV or Advanced Stage IIIB/C Nonsquamous Non-Small Cell Lung Cancers (CodeBreaK 202)
NCT05929222	Comparison Between Local Radiotherapy Alone or Combined With Obinutuzumab in Early Stage Follicular Lymphoma: the GAZEBO Trial From the Fondazione Italiana Linfomi
NCT05933577	A Clinical Study of V940 Plus Pembrolizumab in People With High-Risk Melanoma (V940-001)
NCT05936359	A Study to Evaluate INCA033989 Administered as a Monotherapy or in Combination With Ruxolitinib in Participants With Myeloproliferative Neoplasms
NCT05947851	A Study of Nemtabrutinib Plus Venetoclax vs Venetoclax + Rituximab (VR) in Second-line (2L) + Relapsed/Refractory (R/R) Chronic Lymphocytic Leukemia/Small Lymphocytic Lymphoma (CLL/SLL) (MK-1026-010/BELLWAVE-010)
NCT05948475	Study of Tinengotinib VS. Physician's Choice a Treatment of Subjects With FGFR-altered in Cholangiocarcinoma
NCT05948943	Alpelisib in Pediatric and Adult Patients With Lymphatic Malformations Associated With a PIK3CA Mutation.
NCT05949684	ELEMENT-MDS: A Study to Compare the Efficacy and Safety of Luspatercept in Participants With Myelodysplastic Syndrome (MDS) and Anemia Not Receiving Blood Transfusions
NCT05952037	Study to Evaluate the Efficacy and Safety of Sonrotoclax in Participants With WaldenstrÃ¶m's Macroglobulinemia
NCT05952557	An Adjuvant Endocrine-based Therapy Study of Camizestrant (AZD9833) in ER+/HER2- Early Breast Cancer (CAMBRIA-2)
NCT05963074	A Study to Customize Ibrutinib Treatment Regimens for Participants With Previously Untreated Chronic Lymphocytic Leukemia
NCT05967689	A Study of Zipalertinib in Patients With Advanced Non-Small Cell Lung Cancer With Epidermal Growth Factor Receptor (EGFR) Exon 20 Insertions or Other Uncommon Mutation.
NCT05973773	REZILIENT3 (REsearching ZIpaLertinib In Egfr Non-small Cell Lung Cancer Tumors)
NCT05984277	A Global Study of Volrustomig (MEDI5752) Plus Chemotherapy Versus Pembrolizumab Plus Chemotherapy for Participants With Metastatic Non-small Cell Lung Cancer.
NCT05987332	IDE196 (Darovasertib) in Combination With Crizotinib as First-line Therapy in Metastatic Uveal Melanoma
NCT06012435	A Study of SGN-B6A Versus Docetaxel in Previously Treated Non-small Cell Lung Cancer
NCT06016738	OP-1250 (Palazestrant) vs. Standard of Care for the Treatment of ER+/HER2- Advanced Breast Cancer
NCT06036836	Study of Favezelimab Coformulated With Pembrolizumab (MK-4280A) in Participants With Selected Solid Tumors (MK-4280A-010)
NCT06037980	CisPlatin plUs Gemcitabine and Nabpaclitaxel (GAP) as pReoperative Chemotherapy Versus Immediate Resection in patIents With resecTable BiliarY Tract Cancers (BTC) at High Risk for Recurrence
NCT06045260	""""""Receptor Radionuclide Therapy With 177Lu-DOTATOC"""
NCT06045689	A Study to Assess Luspatercept in Lower-risk Myelodysplastic Syndrome Participants
NCT06045806	A Study to Compare the Efficacy and Safety of Idecabtagene Vicleucel With Lenalidomide Maintenance Therapy Versus Lenalidomide Maintenance Therapy Alone in Adult Participants With Newly Diagnosed Multiple Myeloma Who Have Suboptimal Response After Autologous Stem Cell Transplantation
NCT06054477	Study of ALE.C04 in Patients With Head and Neck Cancer
NCT06054555	A Study to Evaluate ABP 206 Compared With OPDIVOÂ® (Nivolumab) in Subjects With Unresectable or Metastatic Melanoma
NCT06057168	Performance of EluciremÂ® in DSC-MRI Perfusion of Brain Gliomas
NCT06059547	Neoadjuvant Immunotherapy Combined With the Anti-GDF-15 Antibody Visugromab to Treat Muscle Invasive Bladder Cancer
NCT06062420	A Platform Study of Novel Immunotherapy Combinations as First-Line Treatment in Participants With PD-L1 Positive Recurrent/Metastatic Squamous Cell Carcinoma of the Head and Neck- GALAXIES H&N-202
NCT06073821	Study of Sonrotoclax (BGB-11417) Plus Zanubrutinib (BGB-3111) Compared With Venetoclax Plus Obinutuzumab in Participants With Chronic Lymphocytic Leukemia (CLL)
NCT06077760	A Study of V940 Plus Pembrolizumab (MK-3475) Versus Placebo Plus Pembrolizumab in Participants With Non-small Cell Lung Cancer (V940-002)
NCT06078787	Olaparib in PALB2 Advanced Pancreatic Cancer
NCT06079671	Study of Volrustomig in Women With High Risk Locally Advanced Cervical Cancer (eVOLVE-Cervical)
NCT06084936	A Study to Evaluate Glofitamab as a Single Agent vs. Investigator's Choice in Participants With Relapsed/Refractory Mantle Cell Lymphoma
NCT06088290	Study of Lurbinectedin in Combination With Doxorubicin Versus Doxorubicin Alone as First-line Treatment in Participants With Metastatic Leiomyosarcoma
NCT06091254	A Trial to Learn if Odronextamab is Safe and Well-Tolerated and How Well it Works Compared to Rituximab Combined With Different Types of Chemotherapy for Participants With Follicular Lymphoma
NCT06095583	Phase 3 Study of Toripalimab Alone or in Combination With Tifcemalimab as Consolidation Therapy in Patients With Limited-stage Small Cell Lung Cancer (LS-SCLC)
NCT06097364	A Trial to Learn if Odronextamab Combined With Chemotherapy is Safe and Well-Tolerated and How Well it Works Compared to Rituximab Combined With Chemotherapy for Adult Participants With Follicular Lymphoma
NCT06097728	MEDI5752 in Combination With Carboplatin Plus Pemetrexed in Unresectable Pleural Mesothelioma
NCT06101134	A Study to Evaluate Whether Participants With Melanoma Prefer Subcutaneous vs Intravenous Administration of Nivolumab and Nivolumab + Relatlimab Fixed-dose Combinations
NCT06101394	Development of a Fluorescent Visualization System for Non-visible Lung Cancer Nodules
NCT06103864	A Phase III Study of Dato-DXd With or Without Durvalumab Compared With Investigator's Choice of Chemotherapy in Combination With Pembrolizumab in Patients With PD-L1 Positive Locally Recurrent Inoperable or Metastatic Triple-negative Breast Cancer
NCT06109779	Rilvegostomig + Chemotherapy as Adjuvant Therapy for Biliary Tract Cancer After Resection (ARTEMIDE-Biliary01)
NCT06112314	IMC-F106C Regimen Versus Nivolumab Regimens in Previously Untreated Advanced Melanoma (PRISM-MEL-301)
NCT06112379	A Phase III Randomised Study to Evaluate Dato-DXd and Durvalumab for Neoadjuvant/Adjuvant Treatment of Triple-Negative or Hormone Receptor-low/HER2-negative Breast Cancer
NCT06119581	A Study of LY3537982 Plus Immunotherapy With or Without Chemotherapy in Participants With Non-Small Cell Lung Cancer (NSCLC) With a Change in a Gene Called KRAS G12C
NCT06120491	Saruparib (AZD5305) vs Placebo in Men With Metastatic Castration-Sensitive Prostate Cancer Receiving Physician's Choice New Hormonal Agents
NCT06121401	First Line Treatment With Olaparib in Combination With Bevacizumab in HRD Positive Patients
NCT06125522	TACTIVE-U: A Study to Learn About the Study Medicine (Vepdegestrant) When Given With Other Medicines in People With Advanced or Metastatic Breast Cancer. (Sub-Study C)
NCT06129864	A Global Study of Volrustomig (MEDI5752) for Participants With Unresected Locally Advanced Head and Neck Squamous Cell Carcinoma Following Definitive Concurrent Chemoradiotherapy

NCT06132958	Sacituzumab Tirumotecan (MK-2870) in Post Platinum and Post Immunotherapy Endometrial Cancer (MK-2870-005)
NCT06135415	A Study to Evaluate the Efficacy and Safety of Tirbanibulin Ointment in Adult Participants With Actinic Keratosis
NCT06136065	68 Gallium-Fibroblast Activating Protein Inhibitors-46 Positron Emission Tomography - Computerized Tomography for Molecular Assessment of Fibroblast Activation and Risk Assessment in Solid Tumors
NCT06136624	Study of Opevesostat (MK-5684) Versus Alternative NHA in mCRPC (MK-5684-003)
NCT06136650	A Study of Opevesostat (MK-5684) Versus Alternative Next-generation Hormonal Agent (NHA) in Metastatic Castration-resistant Prostate Cancer (mCRPC) Post One NHA (MK-5684-004)
NCT06137118	AZD0486 as Monotherapy in B-cell Acute Lymphoblastic Leukaemia
NCT06137144	AZD3470 as Monotherapy and in Combination With Anticancer Agents in Participants With Relapsed/Refractory Haematologic Malignancies.
NCT06140836	A Study of Repotrectinib Versus Crizotinib in Participants With Locally Advanced or Metastatic Tyrosine Kinase Inhibitor (TKI)-naÃ¯ve ROS1-positive Non-Small Cell Lung Cancer (NSCLC) (TRIDENT-3)
NCT06149286	A Trial to Learn if Odronextamab Combined With Lenalidomide is Safe and Works Better Than Rituximab Combined With Lenalidomide in Adult Participants With Follicular Lymphoma and Marginal Zone Lymphoma
NCT06152575	MagnetisMM-32: A Study to Learn About the Study Medicine Called Elranatamab in People With Multiple Myeloma (MM) That Has Come Back After Taking Other Treatments (Including Prior Treatment With an Anti-CD38 Antibody and Lenalidomide)
NCT06162221	Study of RAS(ON) Inhibitor Combinations in Patients With Advanced RAS-mutated NSCLC
NCT06170788	Sacituzumab Tirumotecan (MK-2870) in Combination With Pembrolizumab Versus Pembrolizumab Alone in Metastatic Non-small Cell Lung Cancer (NSCLC) With Programmed Cell Death Ligand 1 (PD-L1) Tumor Proportion Score (TPS) â‰¥ 50% (MK-2870-007)
NCT06174987	A Study to Provide Continued Access to and Assess Long-Term Safety of the Study Drug(s)
NCT06179160	A Study to Evaluate INCB161734 in Participants With Advanced or Metastatic Solid Tumors With KRAS G12D Mutation
NCT06179511	Study of AZD9829 in CD123+ Hematological Malignancies
NCT06189833	Phase 2 Study Applying MRD Techniques for Participants With Previously Untreated Multiple Myeloma Treated With D-VRd Prior To and After High-dose Therapy Followed by ASCT - TAURUS
NCT06191263	Safety and Efficacy of RVU120 Combined With Venetoclax for Treatment of Relapsed/Refractory AML
NCT06191744	Study of Subcutaneous Epcoritamab in Combination With Intravenous Rituximab and Oral Lenalidomide (R2) to Assess Adverse Events and Change in Disease Activity in Adult Participants With Previously Untreated Follicular Lymphoma
NCT06218511	A Phase I Trial of IMA970A Plus Montanide in Combination With Durvalumab (Anti-PD-L1)
NCT06224244	External Hypofractionated Radiotherapy With Simultaneous Integrated Boost in Early Breast Cancer Patients
NCT06230224	A Trial to Learn How Effective and Safe Odronextamab is Compared to Standard of Care for Adult Participants With Previously Treated Aggressive B-cell Non-Hodgkin Lymphoma
NCT06253650	Adjuvant TRastuzumab Deruxtecan for HER2-positive Gastroesophageal Cancer With Persistence of miNImal Residual Disease
NCT06268574	Safety and Efficacy of RVU120 for Treatment of Relapsed/Refractory AML
NCT06297512	Evaluate the Role of Anthracycline After Radio Therapy in Patients With Glioblastoma (pGBM).
NCT06311695	Contrast-Enhanced Spectral Mammography (CESM)
NCT06341764	Neo-adjuvant Chemo and Immunotherapy in The Pre-operAtive Treatment of Locally Advanced cholangIOcarciNoma
NCT06341842	Potential Protective Role of SGLT-2 Inhibitors for Chemotherapy-induced Cardiotoxicity
NCT06396065	Phase III Study of AK112 for NSCLC Patients
NCT06418087	Durvalumab With Carboplatin and Etoposide Chemotherapy in Pulmonary Large-cell Neuroendocrine Carcinoma (LCNEC)
NCT06464068	AGNOSTIC THERAPY IN A PHASE II SINGLE-ARM STUDY IN FIRST-LINE TREATMENT OF DURVALUMAB IN ASSOCIATION WITH CARBOPLATIN OR CISPLATIN AND ETOPOSIDE IN PATIENTS AFFECTED BY EXTENSIVE STAGE - EXTRAPULMONARY SMALL CELL CARCINOMA
NCT06484361	PET/MRI to Stage Prostate Cancer Patients
NCT06511648	Erdafitinib Monotherapy or in Combination With Cetrelimab in Muscle-invasive Bladder Cancer Patients With Fibroblast Growth Factor Receptor (FGFR) Gene Alterations
NCT05529316	A Study of Botensilimab (AGEN1181) for the Treatment of Advanced Melanoma
NCT05558280	Qarziba for Patients in Relapsed/Refractory High-grade Osteosarcoma
NCT05724602	Radiotherapy Plus Xevinapant in Older Patients With Locally Advanced Head and Neck Squamous Cell Carcinoma
NCT03668392	Asparaginase Activity Monitoring (AAM) in Adult Patients With Acute Lymphoblastic Leukemia (ALL)
NCT03763422	Trial in Low Grade Glioma Patients: Wait or Treat
NCT03850795	HC-1119 Versus Enzalutamide in Metastatic Castration-Resistant Prostate Cancer (mCRPC)
NCT03878719	Study of the Combination of Binimetinib and Encorafenib in Adolescent Patients With Unresectable or Metastatic BRAF V600-mutant Melanoma
NCT03978611	A Study to Assess Safety of Relatlimab With Ipilimumab in Participants With Advanced Melanoma Who Progressed on Anti-Programmed Cell Death Protein 1 (Anti-PD-1) Treatment
NCT04003610	Pemigatinib + Pembrolizumab vs Pemigatinib Alone vs Standard of Care for Urothelial Carcinoma (FIGHT-205)
NCT04093362	Futibatinib Versus Gemcitabine-Cisplatin Chemotherapy as First-Line Treatment of Patients With Advanced Cholangiocarcinoma Harboring FGFR2 Gene Rearrangements
NCT04145700	CAMPFIRE: A Study of Ramucirumab (LY3009806) in Children and Young Adults With Synovial Sarcoma
NCT04197986	Oral Infigratinib for the Adjuvant Treatment of Subjects With Invasive Urothelial Carcinoma With Susceptible FGFR3 Genetic Alterations
NCT04254107	A Safety Study of SEA-TGT (SGN-TGT) in Advanced Cancer
NCT04274023	Study on TSR-042 in Advanced Clear Cell Sarcoma
NCT04294277	Safety and Efficacy of Pemigatinib in Patients With High-risk Urothelial Cancer After Radical Surgery
NCT04311710	A Study Evaluating the Drug Levels of Iplimumab Given Under the Skin Alone and in Combination With Nivolumab in Multiple Tumor Types
NCT04313881	Magrolimab + Azacitidine Versus Azacitidine + Placebo in Untreated Participants With Myelodysplastic Syndrome (MDS)
NCT04323436	Study of Capmatinib and Spartalizumab/Placebo in Advanced NSCLC Patients With MET Exon 14 Skipping Mutations
NCT04324840	A Study of CC-90010 in Combination With Temozolomide With or Without Radiation Therapy in Participants With Newly Diagnosed Glioblastoma
NCT04340193	A Study of Nivolumab and Ipilimumab and Nivolumab Alone in Combination With Trans-arterial ChemoEmbolization (TACE) in Participants With Intermediate Stage Liver Cancer
NCT04390763	Study of Efficacy and Safety of NIS793 (With and Without Spartalizumab) in Combination With SOC Chemotherapy in First-line Metastatic Pancreatic Ductal Adenocarcinoma (mPDAC)
NCT04410445	Study to Compare Adjuvant Immunotherapy of Bempegaldesleukin Combined With Nivolumab Versus Nivolumab After Complete Resection of Melanoma in Patients at High Risk for Recurrence
NCT04428333	Study of GSK3359609 With Pembrolizumab and 5-fluorouracil (5-FU)-Platinum Chemotherapy in Participants With Recurrent or Metastatic Head and Neck Squamous Cell Carcinoma
NCT04478266	Amcenestrant (SAR439859) Plus Palbociclib as First Line Therapy for Patients With ER (+) HER2(-) Advanced Breast Cancer
NCT04479436	A Study to Evaluate U3-1402 in Subjects With Advanced or Metastatic Colorectal Cancer
NCT04489940	Bintrafusp Alfa in High Mobility Group AT-Hook 2 (HMGA2) Expressing Triple Negative Breast Cancer
NCT04508140	Study of BO-112 With Pembrolizumab for Colorectal or Gastric/GEJ Cancer With Liver Metastasis
NCT04515394	Study of Tepotinib Combined With Cetuximab in Participants With Left-Sided RAS/BRAF Wild Type Metastatic Colorectal Cancer (PERSPECTIVE)
NCT04521621	A Study of Gebasaxturev (V937) in Combination With Pembrolizumab (MK-3475) in Participants With Advanced/Metastatic Solid Tumors (V937-013)
NCT04551053	To Evaluate Efficacy and Safety of Parsaclisib and Ruxolitinib in Participants With Myelofibrosis Who Have Suboptimal Response to Ruxolitinib (LIMBER-304)
NCT04586244	An Umbrella Study to Determine the Safety and Efficacy of Various Monotherapy or Combination Therapies in Neoadjuvant Urothelial Carcinoma
NCT04665921	A Study of SGN-STNV in Advanced Solid Tumors
NCT04699461	Study to Evaluate the Efficacy and Safety of Loncastuximab Tesirine Versus Idelalisib in Participants With Relapsed or Refractory Follicular Lymphoma
NCT04729608	Batiraxcept (AVB-S6-500)/Placebo in Combination With Paclitaxel in Patients With Platinum-Resistant Recurrent Ovarian Cancer
NCT04745832	Phase 3 Study of Zandelisib (ME-401) in Combination With Rituximab in Patients With iNHL - (COASTAL)
NCT04752332	A Study of Abemaciclib (LY2835219) Plus Hormone Therapy in Participants With Early Breast Cancer
NCT04778397	Study of Magrolimab in Combination With Azacitidine Versus Physician's Choice of Venetoclax in Combination With Azacitidine or Intensive Chemotherapy in Patients With TP53 Mutant Acute Myeloid Leukemia That Have Not Been Treated
NCT04785547	ALL SCTped 2012 FORUM Add-on Study Blina Post HSCT
NCT04810611	Phase Ib Study of Select Drug Combinations in Patients With Lower Risk MDS
NCT04812548	A Study of Sabatolimab in Combination With Azacitidine and Venetoclax in High or Very High Risk MDS Participants
NCT04854096	Study to Assess Efficacy and Safety of NS-018 Compared to BAT in Patients With Myelofibrosis
NCT04865419	Study of AZD0466 Monotherapy or in Combination in Patients With Advanced Haematological Malignancies
NCT04901806	Study of PBI-200 in Subjects With NTRK-Fusion-Positive Solid Tumors
NCT04908956	Osimertinib and Locally Ablative Radiotherapy in Patients With Synchronous Oligo-metastatic EGFR Mutant NSCLC (STEREO)
NCT04912063	Study to Evaluate Adverse Events and Movement of Lemzoparlimab in Body When Used Intravenously (IV) With Azacitidine Subcutaneously or IV and Venetoclax Orally in Participants With Acute Myeloid Leukemia and With Azacitidine With or Without Venetoclax in Participants With Myelodysplastic Syndrome
NCT04949191	The Purpose of the Study is to Continue to Provide Pemigatinib to Patients With Advanced Malignancies.
NCT04965753	FHD-609 in Subjects With Advanced Synovial Sarcoma or Advanced SMARCB1-Loss Tumors
NCT04969861	BEMPEG With Pembrolizumab vs Pembrolizumab Alone in Patients With Metastatic or Recurrent HNSCC (PROPEL-36)
NCT04979442	Treatment of Milademetan Versus Trabectedin in Patient With Dedifferentiated Liposarcoma

NCT05020665	Entospletinib Plus Intensive Induction/Consolidation Chemotherapy in Newly Diagnosed NPM1-mutated AML
NCT05079230	Study of Magrolimab Versus Placebo in Combination With Venetoclax and Azacitidine in Participants With Acute Myeloid Leukemia
NCT05085002	A Study of Lerociclib in Participants With Advanced Breast Cancer
NCT05132569	Efficacy and Safety of Tolebrutinib (SAR442168) Tablets in Adult Participants With Generalized Myasthenia Gravis
NCT05205161	A Phase I/II Study of AZD0466 as Monotherapy or in Combination With Anticancer Agents in Advanced Non-Hodgkin Lymphoma
NCT05218096	Study of ALXN2050 in Adult Participants With Generalized Myasthenia Gravis
NCT05330429	Study of Magrolimab Given Together With FOLFIRI/BEV in Patients With Previously Treated Advanced Inoperable Metastatic Colorectal Cancer (mCRC)
NCT05447663	A Study of Siremadlin Alone and in Combination With Donor Lymphocyte Infusion in Acute Myeloid Leukemia Post-allogeneic Stem Cell Transplant
NCT05725343	A Prevention Trial of Canakinumab in Subjects at High Risk for Lung Cancer
NCT04231981	Efficacy of INCMGA00012 in Penile Squamous Cell Carcinoma (ORPHEUS)
NCT04238988	Carboplatin-Paclitaxel-Pembrolizumab in Neoadjuvant Treatment of Locally Advanced Cervical Cancer
NCT04427787	A Trial Aiming to Assess the Safety and Activity of the Combination of Cabozantinib Plus Lanreotide in GEP and NET
NCT04502394	Safety and Efficacy of KRT-232 in Combination With Acalabrutinib in Subjects With R/R DLBCL or R/R CLL
NCT04504552	Immune Checkpoint Inhibitor In High Risk Oral Premalignant Lesions
NCT04565496	Phase 2 Study of Neoadjuvant PEMbrolizumab Before Radical PROstatectomy in High-risk Prostate Cancer Patients
NCT04775602	Experimental Study to Evaluate the Impact of 18 Fluoro-PSMA (18F-PSMA) PET / CT in the Management of Patients With Prostate Cancer.
NCT04776655	Study in mCRC Patients RAS/BRAF wt Tissue and RAS Mutated LIquid BIopsy to Compare FOLFIRI Plus CetuxiMAb or BevacizumaB
NCT04793932	Short-course Versus Long-course Pre-operative Chemotherapy With mFOLFIRINOX or PAXG (CASSANDRA TRIAL)
NCT04887961	Reprab Study: PLD + Trabectedin Rechallenge
NCT04996017	Atezolizumab Versus Placebo for the Adjuvant Treatment of Malignant Pleural Mesothelioma (Atezomeso)
NCT05031975	Temozolomide and Irinotecan in Patients With MGMT Silenced Colorectal Cancer After Adjuvant Chemotherapy
NCT05505006	Management of Preoperative Anaemia in Surgical Oncology
NCT05305040	""Study of Posoleucel (ALVR105
NCT03782363	Study of Adoptive Immunotherapy in Relapsed and Non-resectable Sarcomas After Multimodal Treatment.
NCT04264806	A Study of Cusatuzumab in Combination With Azacitidine Compared With Azacitidine Alone in Patients With Higher-risk Myelodysplastic Syndrome (MDS) or Chronic Myelomonocytic Leukemia (CMML) and Who Are Not Candidates for Hematopoietic Stem Cell Transplantation (HSCT)
NCT04495010	Neoadjuvant Nivolumab+Ipilimumab Followed by Adjuvant Nivolumab or Neoadjuvant Nivolumab+Ipilimumab Followed by Adjuvant Observation Compared With Adjuvant Nivolumab in Treatment-Naive High-risk Melanoma Participants
NCT04759846	Hepatic Impairment Study of Encorafenib in Combination With Binimetinib in BRAF Melanoma
NCT05176691	HMPL-760 Safety and Tolerability Study in Patients With Previously Treated CLL/SLL or NHL
NCT05212701	To Assess Efficacy and Safety of Oral Reparixin in Patients With Fatigue and Locally Advanced / Metastatic Breast Cancer
NCT05966233	R-DHAP vs POLA-R-DHAP Followed by Autologous Transplant as First Salvage Treatment in Patient With Relapsed or Refractory Diffuse Large B Cell Lymphoma
NCT06046274	GEN1046 in Combination With Anticancer Agents for the Treatment of Advanced Endometrial Cancer
NCT04873362	A Study Evaluating the Efficacy and Safety of Adjuvant Atezolizumab or Placebo and Trastuzumab Emtansine for Participants With HER2-Positive Breast Cancer at High Risk of Recurrence Following Preoperative Therapy,
TOTAL STUDIES 962	

supplemental table S1: list of clinical trials on neoplastic diseases extracted from the 'clinicaltrials.go' database
