# Supplementary material for: Geographic disparities in gastrointestinal oncology research: a focus on trial availability in Italy
Source: Oncologist. 2025 Mar 27;30(3):oyaf011. doi: 10.1093/oncolo/oyaf011 (PMC11950913; doi:10.1093/oncolo/oyaf011)
Supplement: oyaf011_suppl_Supplementary_Tables_S2-S22 [file oyaf011_suppl_supplementary_tables_s2-s22.pdf]

STUDY	CONDITIONS	CONDITION
NCT06341764	Cholangiocarcinoma	CHOLANGIOCARCINOMA
NCT06253650	Gastric Cancer|HER2-positive Gastric Cancer	GASTRIC-ESOPHAGEAL CANCER
NCT06218511	Hepatocellular Carcinoma	HEPATOCELLULAR CARCINOMA (HCC)
NCT06136065	Solid Tumor, Unspecified, Adult	VARIOUS GI CANCER
NCT06115629	Esophageal Cancer|Gastric Cancer	GASTRIC-ESOPHAGEAL CANCER
NCT06109779	Biliary Tract Cancer	BILIARY TRACT CANCER
NCT06078787	Advanced Pancreatic Cancer|Metastatic Pancreatic Cancer	PANCREATIC CANCER
NCT06037980	Biliary Tract Cancer|Cholangiocarcinoma	CHOLANGIOCARCINOMA
NCT05948475	Cholangiocarcinoma	CHOLANGIOCARCINOMA
NCT05883644	Advanced Hepatocellular Carcinoma	HEPATOCELLULAR CARCINOMA (HCC)
NCT05876754	Cholangiocarcinoma	CHOLANGIOCARCINOMA
NCT05867121	Metastatic Solid Tumor|Non-small Cell Lung Cancer|Gastric Cancer|Pancreatic Ductal Adenocarcinoma	PANCREATIC CANCER
NCT05855200	Colonic Neoplasms|Neoplasms, Colon	COLORECTAL CANCER
NCT05845450	Colorectal Cancer|Resectable Colorectal Carcinoma	COLORECTAL CANCER
NCT05836324	Solid Tumors|Advanced Solid Tumors|Metastatic Solid Tumors	COLORECTAL CANCER
NCT05821556	Adenocarcinoma of the Pancreas	PANCREATIC CANCER
NCT05775159	Hepatocellular Carcinoma|Biliary Tract Cancer	HEPATOCELLULAR CARCINOMA (HCC)
NCT05771480	Biliary Tract Cancer	BILIARY TRACT CANCER
NCT05743036	Metastatic Colorectal Cancer	COLORECTAL CANCER
NCT05734105	GIST	VARIOUS GI CANCER
NCT05727176	Advanced Cholangiocarcinoma|FGFR2 Fusions|Gene Rearrangement	CHOLANGIOCARCINOMA
NCT05723562	Neoplasms, Rectal	RECTAL CANCER
NCT05678257	Colorectal Cancer|Colorectal Neoplasms|Colorectal Adenocarcinoma|Colorectal Cancer Metastatic|Neoplasm, Colorectal	COLORECTAL CANCER
NCT05630937	Unresectable Hepatocellular Carcinoma (HCC)	HEPATOCELLULAR CARCINOMA (HCC)
NCT05608044	Metastatic Colorectal Cancer	COLORECTAL CANCER
NCT05586516	Metastatic Pancreatic Cancer	PANCREATIC CANCER
NCT05480865	Solid Tumor, Adult|Metastatic Solid Tumor|Metastatic NSCLC|Non Small Cell Lung Cancer	COLORECTAL CANCER
NCT05466799	Locally Advanced Pancreatic Cancer	PANCREATIC CANCER
NCT05440864	Hepatocellular Carcinoma	HEPATOCELLULAR CARCINOMA (HCC)
NCT05383352	Metastatic Pancreatic Adenocarcinoma	PANCREATIC CANCER
NCT05382741	Colorectal Cancer Stage IV|No Evidence of Disease State	COLORECTAL CANCER
NCT05379595	Advanced or Metastatic Colorectal Cancer	COLORECTAL CANCER
NCT05365581	Gastric Adenocarcinoma|Gastroesophageal Junction (GEJ) Adenocarcinoma|Pancreatic Adenocarcinoma	PANCREATIC CANCER
NCT05358249	KRAS G12C Mutant Solid Tumors|Carcinoma, Non-Small Cell Lung|Carcinoma, Non-Small-Cell Lung|Non-Small Cell Lung Cancer|Non-Small Cell Lung Carcinoma|Nonsmall Cell Lung Cancer|Colorectal Cancer|Colorectal Carcinoma|Colorectal Neoplasms|Colorectal Tumors|Neoplasms, Colorectal	COLORECTAL CANCER
NCT05342636	Esophageal Squamous Cell Carcinoma (ESCC)	GASTRIC-ESOPHAGEAL CANCER
NCT05330429	Metastatic Colorectal Cancer	COLORECTAL CANCER
NCT05328908	Colorectal Neoplasms	COLORECTAL CANCER
NCT05319730	Esophageal Squamous Cell Carcinoma	GASTRIC-ESOPHAGEAL CANCER
NCT05312398	Metastatic Colorectal Adenocarcinoma	COLORECTAL CANCER
NCT05301842	Hepatocellular Carcinoma	HEPATOCELLULAR CARCINOMA (HCC)
NCT05291156	Metastatic Colorectal Cancer	COLORECTAL CANCER
NCT05253846	Locally Advanced Rectal Cancer	COLORECTAL CANCER
NCT05253651	Colorectal Neoplasms	COLORECTAL CANCER
NCT05217446	Metastatic Colorectal Cancer	COLORECTAL CANCER
NCT05208047	Advanced Gastrointestinal Stromal Tumors|Metastatic Cancer	VARIOUS GI CANCER
NCT05198934	Colorectal Cancer (CRC)	COLORECTAL CANCER
NCT05152147	Gastric Neoplasms|Gastroesophageal Adenocarcinoma|Esophageal Adenocarcinoma	GASTRIC-ESOPHAGEAL CANCER
NCT05136326	Rectal Cancer	RECTAL CANCER
NCT05111626	Gastric Cancer|Gastroesophageal Junction Adenocarcinoma	GASTRIC-ESOPHAGEAL CANCER
NCT05064059	Colorectal Cancer	COLORECTAL CANCER
NCT05062889	Stage II Colon Cancer|Stage III Colon Cancer|HER2-positive Colon Cancer|RAS Wild-type Colon Cancer	COLORECTAL CANCER
NCT05059522	Advanced Malignancies|NSCLC|Ovarian Cancer|Urothelial Cancer|Solid Tumors	COLORECTAL CANCER
NCT05052801	Gastric Cancer|Gastroesophageal Junction Adenocarcinoma	GASTRIC-ESOPHAGEAL CANCER
NCT05050942	Gastro-enteropancreatic Neuroendocrine Tumor	PANCREATIC CANCER
NCT05031975	Colorectal Cancer	COLORECTAL CANCER
NCT05002127	Gastric Cancer|Gastroesophageal Junction Adenocarcinoma|Gastric Adenocarcinoma	GASTRIC-ESOPHAGEAL CANCER
NCT04952753	Metastatic Colorectal Cancer	COLORECTAL CANCER
NCT04949256	Metastatic Esophageal Squamous Cell Carcinoma	GASTRIC-ESOPHAGEAL CANCER
NCT04949191	Advanced Malignancies	VARIOUS GI CANCER
NCT04935359	Metastatic Pancreatic Ductal Adenocarcinoma	PANCREATIC CANCER
NCT04929223	Metastatic Colorectal Cancer	COLORECTAL CANCER
NCT04920149	Lynch Syndrome|Colon Cancer|Colon Neoplasm	COLORECTAL CANCER
NCT04901806	Solid Tumor, Adult|Brain Tumor, Primary|Desmoplastic Small Round Cell Tumor	COLORECTAL CANCER
NCT04895722	Colorectal Cancer	COLORECTAL CANCER
NCT04879368	Gastro-Oesophageal Cancer	GASTRIC-ESOPHAGEAL CANCER
NCT04817826	Gastric Cancer|Microsatellite Instability	GASTRIC-ESOPHAGEAL CANCER
NCT04803994	Hepatocellular Carcinoma	HEPATOCELLULAR CARCINOMA (HCC)
NCT04793958	Advanced Colorectal Cancer|Metastatic Colorectal Cancer	COLORECTAL CANCER
NCT04787341	Colorectal Cancer	COLORECTAL CANCER
NCT04785820	Advanced or Metastatic Esophageal Squamous Cell Carcinoma	GASTRIC-ESOPHAGEAL CANCER
NCT04776655	Colorectal Cancer|Metastatic Colorectal Cancer|RAS Mutation	COLORECTAL CANCER
NCT04773782	Solid Tumor, Unspecified, Child|Relapsed Solid Neoplasm|CNS Tumor	COLORECTAL CANCER
NCT04770896	Unresectable Hepatocellular Carcinoma	HEPATOCELLULAR CARCINOMA (HCC)
NCT04744831	Advanced Colorectal Cancer	COLORECTAL CANCER
NCT04740307	Advanced Hepatocellular Carcinoma	HEPATOCELLULAR CARCINOMA (HCC)
NCT04737187	Refractory Metastatic Colorectal Cancer	COLORECTAL CANCER
NCT04704934	Gastric Cancer, Adenocarcinoma|Gastroesophageal Junction Adenocarcinoma	GASTRIC-ESOPHAGEAL CANCER
NCT04699188	KRAS G12C Mutant Solid Tumors|Carcinoma, Non-Small-Cell Lung|Carcinoma, Colorectal|Cancer of Lung|Cancer of the Lung|Lung Cancer|Neoplasms, Lung|Neoplasms, Pulmonary|Pulmonary Cancer|Pulmonary Neoplasms	COLORECTAL CANCER
NCT04696055	Hepatocellular Carcinoma	HEPATOCELLULAR CARCINOMA (HCC)
NCT04660812	Metastatic Colorectal Cancer	COLORECTAL CANCER
NCT04639219	Advanced Solid Tumors With HER2 Mutation,eg:Colorectal,Urothelial,Gastric, Hepatobiliary,Endometrial,Melanoma,Ovarian,Cervical,Salivary Gland,Pancreatic,Breast	VARIOUS GI CANCER
NCT04622774	Advanced Solid Tumor	COLORECTAL CANCER
NCT04564898	Colorectal Cancer Metastatic	COLORECTAL CANCER
NCT04543617	Esophageal Squamous Cell Carcinoma	COLORECTAL CANCER
NCT04524442	Gastroenteropancreatic Neuroendocrine Tumors	COLORECTAL CANCER
NCT04523493	Advanced Hepatocellular Carcinoma (HCC)	COLORECTAL CANCER
NCT04515394	Colorectal Neoplasms	COLORECTAL CANCER
NCT04513951	Metastatic Colorectal Cancer	COLORECTAL CANCER
NCT04508140	Colorectal Cancer|Gastric Cancer|Oesophageal Cancer	COLORECTAL CANCER
NCT04495621	Metastatic Colorectal Cancer	COLORECTAL CANCER
NCT04479436	Metastatic Colorectal Cancer	COLORECTAL CANCER
NCT04472429	Squamous Cell Carcinoma of the Anal Canal	COLORECTAL CANCER
NCT04466891	HER2-amplified Biliary Tract Cancers	COLORECTAL CANCER
NCT04449874	Non-Small Cell Lung Cancer|Colorectal Cancer|Advanced Solid Tumors	COLORECTAL CANCER
NCT04408118	Metastatic Breast Cancer|Advanced Breast Cancer|Triple Negative Breast Cancer	COLORECTAL CANCER
NCT04390763	Metastatic Pancreatic Ductal Adenocarcinoma	COLORECTAL CANCER
NCT04379596	Gastric Cancer	COLORECTAL CANCER
NCT04322539	Metastatic Colorectal Cancer|Metastatic Colon Cancer	COLORECTAL CANCER
NCT04311710	Tumor	HEPATOCELLULAR CARCINOMA (HCC)
NCT04259944	Colon Cancer	COLORECTAL CANCER
NCT04189445	Advanced or Metastatic Solid Tumor|Advanced or Metastatic Gastric or Gastroesophageal Cancer|Myeloid or Lymphoid Neoplasms (MLN)	COLORECTAL CANCER
NCT04093362	Advanced Cholangiocarcinoma|FGFR2 Gene Rearrangements	CHOLANGIOCARCINOMA
NCT02974556	Colon Cancer|Intraperitoneal Rectal Cancer	COLORECTAL CANCER

TOTAL STUDIES 103

supplemental table S2: list of clinical trials on GI cancers active in Italy as extracted from the ‘clinicaltrials.gov’ database
