# Supplementary material for: Geographic disparities in gastrointestinal oncology research: a focus on trial availability in Italy
Source: Oncologist. 2025 Mar 27;30(3):oyaf011. doi: 10.1093/oncolo/oyaf011 (PMC11950913; doi:10.1093/oncolo/oyaf011)
Supplement: oyaf011_suppl_Supplementary_Tables_S3 [file oyaf011_suppl_supplementary_tables_s3.pdf]

Clinical Center	Number of active studies
Fondazione IRCCS Ca' Granda Ospedale Maggiore Policlinico	5
A.O.R.N. San Giuseppe Moscati	3
A.R.N.A.S. Ospedali Civico Di Cristina Benfratelli	1
AO San Giovanni Addolorata	2
AORN – Ospedali dei Colli	1
AOU "Ospedali Riuniti" di Foggia	1
AOU Ferrara Arcispedale Sant'Anna	1
AOU Friuli Centrale	4
AOU Maggiore della Carità	3
AOU Mater Domini	3
AOU Policlinico "Paolo Giaccone"	4
AOU Policlinico di Modena	6
AOU Policlinico Umberto I	1
AOUI Verona Policlinico Rossi	1
ARNAS Garibaldi Catania	7
ASL Biella - Nuovo Ospedale degli Infermi	1
ASL LE-Ospedale Vito Fazi	4
ASL Napoli 1 Centro	1
ASL Napoli 1 Centro - P.O. Ospedale del Mare	1
ASL5 Spezzino - Ospedale Civile Sant'Andrea	1
ASP Ragusa	2
ASST Cremona	1
ASST degli Spedali Civili di Brescia	5
ASST Grande Ospedale Metropolitano Niguarda	25
ASST Nord Milano PO Città di Sesto S. Giovanni	1
ASST Ospedale Papa Giovanni XXIII	2
ASST Valtellina e Alto Lario	1
AULSS Berica 8	3
AUSL di Reggio Emilia - Ospedale Civile di Guastalla	2
AUSL Reggio Emilia	1
AUSL Reggio Emilia Arcispedale Santa Maria Nuova	6
Azieda ULSS 8 Berica - Ospedale San Bortolo	3
Azienda Ospedaliera Cardinale Panico	7
Azienda Ospedaliera di Rilievo Nazionale Antonio Cardarelli	1
Azienda Ospedaliera Ordine Mauriziano di Torino	8
Azienda Ospedaliera Regionale San Carlo	4
Azienda Ospedaliera S. Croce e Carle	3
Azienda Ospedaliera San Camillo Forlanini	5
Azienda Ospedaliera Sant'Andrea	1
Azienda Ospedaliera Universitaria Integrata Verona	10
Azienda Ospedaliera Universitaria Luigi Vanvitelli	18
Azienda Ospedaliera Universitaria Senese Policlinico Santa Maria Alle Scotte	6
Azienda Ospedaliero - Universitaria di Parma	2
Azienda Ospedaliero Universitaria Careggi	16
Azienda Ospedaliero Universitaria Ospedali Riuniti Umberto I	10
Azienda Ospedaliero-Universitaria Città della Salute e della Scienza di Torino	3
Azienda Ospedaliero-Universitaria di Cagliari	10
Azienda ospedaliero-universitaria pisana	27
Azienda Sanitaria universitaria Friuli centrale PO Santa maria della Misericordia	2
Azienda ULSS3 Serenissima	1
Azienda USL della Romagna	2
Azienda USL della Romagna - Ospedale "Santa Maria delle Croci" di Ravenna	1
Azienda USL di Piacenza	1
Azienda USL IRCCS di Reggio Emilia	8
Azienda USL Toscana centro	1
Azienda USL Toscana nord ovest Ospedali Riuniti di Livorno	2
Azienda Usl Toscana sud est - Ospedale Misericordia di Grosseto	1
Azienda Usl Toscana sud est - Ospedale San Donato di Arezzo	1
Centro di Riferimento Oncologico IRCCS di Aviano	3
Centro Ricerche Cliniche di Verona	3
Dip Oncologia Medica ed Ematologia-SC Pediatria oncologica	1
Dip. Medicina e chirurgia Università di Bologna	1
Ente Ospedaliero Ospedali Galliera	1
Fondazione IRCCS Policlinico San Matteo	2
Fondazione Poliambulanza Istituto Ospedaliero	8
Fondazione Policlinico Universitario Agostino Gemelli IRCCS	15
I.R.C.C.S. "Giovanni Paolo II" - Istituto oncologico	1
INT Pascale	1
IRCCS Casa Sollievo della Sofferenza	12
IRCCS Ospedale San Raffaele	14
Istituto Clinico Humanitas	22
Istituto di Candiolo - Fondazione del Piemonte per l'Oncologia - IRCCS	9
Istituto Europeo di Oncologia	20
Istituto Nazionale dei Tumori	37
Istituto Nazionale Tumori IRCCS Fondazione G. Pascale	25
Istituto Nazionale Tumori Regina Elena	3
Istituto Oncologico Veneto (IRCCS)	30
Istituto Romagnolo per lo Studio dei Tumori "Dino Amadori"	17
Nuovo Ospedale di Prato - S. Stefano	1
Oncologia Universitara	1
Ospedale "A. Perrino"	2
Ospedale "degli Infermi" di Faenza	1
Ospedale Borgo Roma	11
Ospedale Campostaggia Poggibonsi	1
Ospedale dell'Aquila - Presidio Ospedaliero "San Salvatore"	1
Ospedale Generale Regionale 'F. Miulli'	1
Ospedale Infermi di Rimini	2
Ospedale IRCCS Saverio De Bellis	4
Ospedale Isola Tiberina - Gemelli Isola	1
Ospedale Policlinico San Martino IRCCS	9
Ospedale Sacro Cuore di Gesù - Fatebenefratelli	1
Ospedale Sacro Cuore Don Calabria	1
Ospedale San Gerardo Monza	1
Ospedale San Giuseppe Moscati	2
Ospedale San Luca di Lucca	1
Ospedale Santa Maria della Misericordia	1
Ospedale Sant'Eugenio	1
Ospedale Versilia - Camaiore	2
Policlinico S.Orsola-Malpighi	10
Policlinico Tor Vergata	2
Policlinico Universitario Campus Biomedico	4
Res site	81
Seconda Università degli Studi di Napoli	1
Università Cattolica del Sacro Cuore	3
Università degli studi di Bari	1
Università degli studi di Genova- Center of Excellence for Biomedical Research CEBR	1
Università degli Studi di Napoli Federico II	1
Università della Sapienza	1
Università di Modena	1
(vuoto)	
**TOTAL**	**630**

supplemental table S3: list of Italian centres where studies under investigation are conducted.
