# Supplementary material for: Geographic disparities in gastrointestinal oncology research: a focus on trial availability in Italy
Source: Oncologist. 2025 Mar 27;30(3):oyaf011. doi: 10.1093/oncolo/oyaf011 (PMC11950913; doi:10.1093/oncolo/oyaf011)
Supplement: oyaf011_suppl_Supplementary_Tables_S6 [file oyaf011_suppl_supplementary_tables_s6.pdf]

ID	Area	Width	CAP	Population
1	120.682053	10	00123	115,138
2	3017.030762	50	00123	3,667,365
3	12067.84668	100	00123	5,147,258
4	120.682266	10	06121	153,304
5	3017.030029	50	06121	741,763
6	12067.8457	100	06121	2,779,175
7	120.682106	10	09021	16,486
8	3017.031494	50	09021	497,991
9	12067.85156	100	09021	1,198,994
10	120.682312	10	09042	330,947
11	3017.030029	50	09042	637,460
12	12067.8457	100	09042	1,003,107
13	120.682121	10	10043	527,567
14	3017.030518	50	10043	2,345,817
15	12067.84473	100	10043	3,908,875

16	120.682373	10	10060	45,918
17	3017.030762	50	10060	2,341,863
18	12067.8457	100	10060	3,736,837
19	120.682495	10	10123	1,161,889
20	3017.029053	50	10123	2,525,876
21	12067.8418	100	10123	4,330,357
22	120.682396	10	12100	82,123
23	3017.030029	50	12100	568,944
24	12067.84473	100	12100	3,476,296
25	120.68219	10	13900	114,347
26	3017.030518	50	13900	974,422
27	12067.84473	100	13900	8,378,814
28	120.682426	10	16121	552,751
29	3017.030029	50	16121	1,155,969
30	12067.8457	100	16121	3,175,787
31	120.682396	10	19121	147,062

32	3017.030518	50	19121	705,116
33	12067.84668	100	19121	3,582,947
34	120.68235	10	20021	1,280,209
35	3017.029541	50	20021	6,556,665
36	12067.84277	100	20021	9,935,858
37	120.682213	10	20089	779,949
38	3017.029541	50	20089	6,093,264
39	12067.84375	100	20089	10,133,614
40	120.682472	10	20900	913,435
41	3017.030518	50	20900	6,601,741
42	12067.8457	100	20900	9,974,726
43	120.682579	10	23100	48,859
44	3017.03125	50	23100	548,144
45	12067.84473	100	23100	7,377,136
46	120.682373	10	24121	388,672
47	3017.030518	50	24121	5,345,026

48	12067.84375	100	24121	9,716,568
49	120.682289	10	25121	326,734
50	3017.029297	50	25121	2,213,488
51	12067.84277	100	25121	10,078,666
52	120.682381	10	26100	98,268
53	3017.029541	50	26100	1,794,890
54	12067.84375	100	26100	9,936,303
55	120.682365	10	27100	118,575
56	3017.030518	50	27100	4,597,063
57	12067.8457	100	27100	10,934,563
58	120.682495	10	28100	151,839
59	3017.030762	50	28100	4,877,667
60	12067.84473	100	28100	10,745,339
61	120.682518	10	29121	124,397
62	3017.03125	50	29121	1,301,950
63	12067.84668	100	29121	10,707,345

64	120.68222	10	30035	226,184
65	3017.030273	50	30035	2,686,064
66	12067.84277	100	30035	5,432,471
67	120.682419	10	31033	121,801
68	3017.029297	50	31033	2,857,351
69	12067.84375	100	31033	5,628,175
70	120.68235	10	33081	25,558
71	3017.031738	50	33081	1,158,702
72	12067.8457	100	33081	3,850,202
73	120.682472	10	33100	163,155
74	3017.030518	50	33100	911,533
75	12067.8457	100	33100	2,400,268
76	120.682343	10	35128	380,010
77	3017.030518	50	35128	2,793,432
78	12067.8457	100	35128	5,827,662
79	120.682274	10	36100	202,016

80	3017.030762	50	36100	2,661,828
81	12067.8457	100	36100	6,231,396
82	120.682381	10	37024	153,112
83	3017.031738	50	37024	1,903,175
84	12067.8457	100	37024	7,145,789
85	120.682152	10	37134	329,059
86	3017.030518	50	37134	1,867,520
87	12067.84473	100	37134	7,464,915
88	120.68232	10	40121	489,735
89	3017.030273	50	40121	1,909,157
90	12067.84473	100	40121	6,005,513
91	120.682503	10	41121	211,673
92	3017.029541	50	41121	2,076,109
93	12067.84473	100	41121	6,367,138
94	120.682182	10	42016	71,439
95	3017.030273	50	42016	1,794,593

96	12067.84473	100	42016	6,323,950
97	120.682381	10	42121	171,659
98	3017.030273	50	42121	1,642,876
99	12067.84375	100	42121	6,272,812
100	120.682297	10	43121	177,805
101	3017.032227	50	43121	1,425,826
102	12067.8457	100	43121	6,242,657
103	120.682274	10	44121	134,907
104	3017.029297	50	44121	1,616,968
105	12067.84277	100	44121	6,583,420
106	120.682205	10	47014	28,145
107	3017.030273	50	47014	1,080,945
108	12067.84375	100	47014	4,394,909
109	120.682106	10	47921	161,439
110	3017.030029	50	47921	1,002,241
111	12067.8457	100	47921	2,513,460

112	120.682289	10	48018	70,334
113	3017.031006	50	48018	1,243,818
114	12067.84766	100	48018	5,009,727
115	120.68222	10	48121	100,779
116	3017.030518	50	48121	1,001,279
117	12067.84473	100	48121	3,301,426
118	120.682114	10	50121	499,724
119	3017.030273	50	50121	1,758,498
120	12067.84668	100	50121	5,021,471
121	120.68235	10	52100	85,966
122	3017.030029	50	52100	648,760
123	12067.84375	100	52100	3,950,540
124	120.682381	10	53100	70,837
125	3017.030762	50	53100	686,990
126	12067.84668	100	53100	3,560,541
127	120.682373	10	55041	124,303

128	3017.029785	50	55041	1,492,035
129	12067.8457	100	55041	4,283,522
130	120.682106	10	55100	126,907
131	3017.031006	50	55100	1,610,103
132	12067.84766	100	55100	4,294,182
133	120.682335	10	56121	131,591
134	3017.029785	50	56121	1,291,274
135	12067.8457	100	56121	3,367,257
136	120.682312	10	57121	168,235
137	3017.030029	50	57121	1,086,539
138	12067.84473	100	57121	3,283,525
139	120.682274	10	58100	65,312
140	3017.030029	50	58100	263,861
141	12067.8457	100	58100	1,342,523
142	120.682373	10	59100	324,484
143	3017.030273	50	59100	1,866,732

144	12067.84473	100	59100	5,389,242
145	120.682388	10	60121	118,418
146	3017.029785	50	60121	797,759
147	12067.84668	100	60121	1,955,775
148	120.682312	10	67100	67,831
149	3017.030518	50	67100	505,863
150	12067.84766	100	67100	5,625,521
151	120.682365	10	70013	70,422
152	3017.031006	50	70013	1,482,178
153	12067.84668	100	70013	2,908,486
154	120.682358	10	70121	398,653
155	3017.031006	50	70121	1,312,100
156	12067.84863	100	70121	2,747,093
157	120.682304	10	70123	368,468
158	3017.03125	50	70123	1,375,528
159	12067.8457	100	70123	2,755,974

160	120.682365	10	71013	26,969
161	3017.032227	50	71013	623,788
162	12067.85059	100	71013	1,829,198
163	120.682663	10	71121	150,440
164	3017.030762	50	71121	683,228
165	12067.84766	100	71121	2,403,411
166	120.682312	10	72100	88,743
167	3017.030762	50	72100	879,312
168	12067.84668	100	72100	2,136,354
169	120.682518	10	73039	72,025
170	3017.031006	50	73039	669,104
171	12067.84961	100	73039	1,169,441
172	120.68222	10	73100	121,186
173	3017.030273	50	73100	961,969
174	12067.84863	100	73100	1,768,551
175	120.68222	10	74100	121,186

176	3017.030273	50	74100	961,969
177	12067.84863	100	74100	1,768,551
178	120.682327	10	80131	1,767,374
179	3017.030029	50	80131	4,609,803
180	12067.84961	100	80131	6,063,010
181	120.682297	10	82100	86,087
182	3017.030518	50	82100	2,193,677
183	12067.84766	100	82100	6,753,555
184	120.682396	10	83100	143,685
185	3017.031738	50	83100	4,413,169
186	12067.84863	100	83100	6,519,213
187	120.682396	10	85100	80,351
188	3017.029785	50	85100	474,212
189	12067.84766	100	85100	3,375,287
190	120.682228	10	88100	108,487
191	3017.031006	50	88100	643,632

192	12067.84766	100	88100	1,584,026
193	120.682297	10	90121	574,729
194	3017.030029	50	90121	1,227,439
195	12067.84766	100	90121	2,259,444
196	120.682335	10	95121	307,790
197	3017.03125	50	95121	1,328,540
198	12067.85156	100	95121	3,062,958
199	120.682419	10	97100	102,949
200	3017.030029	50	97100	693,003
201	12067.84961	100	97100	2,204,136

supplemental table S6: average population within 10, 50 and 100 km, per geographic area, of the trial site (n= 67). the ‘,’ represents the divisor of thousands
