# Supplementary material for: Geographic disparities in gastrointestinal oncology research: a focus on trial availability in Italy
Source: Oncologist. 2025 Mar 27;30(3):oyaf011. doi: 10.1093/oncolo/oyaf011 (PMC11950913; doi:10.1093/oncolo/oyaf011)
Supplement: oyaf011_suppl_Supplementary_Tables_S5 [file oyaf011_suppl_supplementary_tables_s5.pdf]

SPONSOR	N. OF studies	% OF SPONSOR PER TYPOLOGY
INDUSTRY	73	70,87%
NETWORK	1	0,97%
OTHER (NO-Profit)	28	27,18%
OTHER_GOV	1	0,97%
**TOTAL**	103	100,00%

supplemental table S5: Classification of CTs under investigation by Sponsor Typology
