# Supplementary material for: Geographic disparities in gastrointestinal oncology research: a focus on trial availability in Italy
Source: Oncologist. 2025 Mar 27;30(3):oyaf011. doi: 10.1093/oncolo/oyaf011 (PMC11950913; doi:10.1093/oncolo/oyaf011)
Supplement: oyaf011_suppl_Supplementary_Tables_4 [file oyaf011_suppl_supplementary_tables_4.pdf]

PHASE OF CT	*N. OF STUDIES*	*% OF STUDIES PER PHASE*
PHASE1	10	9,71%
PHASE1|PHASE2	13	12,62%
PHASE2	43	41,75%
PHASE2|PHASE3	2	1,94%
PHASE3	34	33,01%
PHASE4	1	0,97%
TOTAL	103	100,00%

supplemental table S4: Classification of CTs under investigation by study phase (1 to 4)
